# Globalization of Perturbative Chern–Simons Theory on the Moduli Space of Flat Connections in the BV Formalism

**DOI:** 10.1007/s00220-026-05727-w

**Published:** 2026-08-28

**Authors:** Pavel Mnev, Konstantin Wernli

**Affiliations:** 1 University of Notre Dame, Notre Dame, IN 46556 USA; 2 Institut für Mathematik, Universität Zürich, Winterthurerstrasse 190, 8057 Zürich, Switzerland; 3 Centre for Quantum Mathematics, IMADA, University of Southern Denmark, Campusvej 55, 5230 Odense M, Denmark

## Abstract

We study the perturbative path integral of Chern–Simons theory (the effective BV action on zero-modes) in Lorenz gauge, expanded around a (possibly non-acyclic) flat connection, as a family over the smooth irreducible stratum $${\mathcal {M}}'\subset {\mathcal {M}}$$ of the moduli space of flat connections. We prove that it is horizontal with respect to the Grothendieck connection up to a BV-exact term. From it, we construct a volume form on $${\mathcal {M}}'$$—the “global partition function”—whose cohomology class is independent of the metric, and so is a 3-manifold invariant. As an element of the construction, we construct an extension of the perturbative partition function to a nonhomogeneous form on the space of triples $\left(A,A^{\prime },g\right)$ consisting of (1) a “kinetic” flat connection *A* around which Chern–Simons action is expanded, (2) a “gauge-fixing” flat connection $A^{\prime }$, (3) a metric *g*. This extension is horizontal with respect to an appropriate Gauss-Manin superconnection (which involves the BV operator as a degree zero component).

## Introduction

The Chern–Simons field theory has been a major focus of interest of the mathematical physics community since the discovery of its close links to invariants of knots and 3-manifolds, both in non-perturbative [Wit89, RT91] and perturbative [FK89, Kon93b] treatments of the theory. An early breakthrough by Axelrod and Singer [AS91, AS94] was the result that the perturbative series at acyclic flat connections is well defined and yields topological invariants of the spacetime three-manifold equipped with framing. In this work, we generalize this result to smooth irreducible components of the moduli space: We show that these carry a volume form (valued in formal power series) whose cohomology class (total volume of the component) is a topological invariant of the (framed) spacetime three-manifold. Somewhat surprisingly, in the construction of this volume form, extra corrections beyond the usual Feynman diagrams are needed.

### Perturbative Chern–Simons partition function at a non-acyclic flat connection

Fix a closed oriented 3-manifold *M* and a compact simply-connected matrix Lie group *G* with Lie algebra ${\mathfrak {g}}$.

We consider Chern–Simons theory, defined classically by the action functional

1 $$\begin{aligned} S_{CS}(A)=\int _M \textrm{tr} \left( \frac{1}{2} A\wedge dA +\frac{1}{6} A\wedge [A,A] \right) \end{aligned}$$

on the space of connections *A* in the trivial *G*-bundle *P* on *M*, which are identified with ${\mathfrak {g}}$-valued 1-forms on *M*. The critical points of $S_{CS}$ are *flat* connections.

In Batalin–Vilkovisky (BV) formalism, one replaces (1) by the “master action” given by the same formula, but with field *A* replaced with a nonhomogeneous ${\mathfrak {g}}$-valued form $\mathcal {A}$ on *M*, see [AKSZ97].

#### Path integral heuristics

We are interested in the Chern–Simons path integral over gauge equivalence classes of connections

2 $$\begin{aligned} \int _{\textrm{Conn}(P)/\textrm{Gauge}} \mathcal {D}A\; e^{\frac{i}{\hbar }S_{CS}(A)}. \end{aligned}$$

The perturbative (stationary phase) contribution of an acyclic flat connection to the $\hbar \rightarrow 0$ asymptotics of (2) was studied in [Wit89] (one-loop approximation) and [AS91, AS94] (higher-loop contributions).

Given a non-acyclic flat connection $A_0$, one can decompose fields in the neighborhood of $A_0$ as $\mathcal {A}=A_0+{\textsf{a}}+\alpha _\textrm{fl}$, with ${\textsf{a}}$ a $d_{A_0}$-cohomology class (represented by a harmonic form) and $\alpha _\textrm{fl}$ a fluctuation. Then, one considers the path integral

3 $$\begin{aligned} Z_{A_0}({\textsf{a}})= \int \mathcal {D}\alpha _\textrm{fl}\; e^{\frac{i}{\hbar }S_\textrm{CS}(A_0+{\textsf{a}}+\alpha _\textrm{fl})} \end{aligned}$$

where the integration is over field fluctuations—$d^*_{A_0}$-exact forms $\alpha _\textrm{fl}\in \Omega ^\bullet (M,{\mathfrak {g}})$.^1^ In the case $A_0=0$, perturbative expansion for the path integral (3) was constructed and studied in [CM08], as an effective BV action in ${\textsf{a}}$. For nonzero $A_0$ the construction is spelled out in [Wer22].

A related idea is that in the path integral (2) one might want to consider a tubular neighborhood of the moduli space of flat connections and integrate over fibers, producing a volume form of the moduli space.

In this paper we will be denoting the perturbative evaluation of (3) $Z_{A_0}({\textsf{a}})$ and the volume form on the moduli space as above $Z^\textrm{glob}$—we will define both objects mathematically, without reference to heuristic path integral expressions. They are linked by

$$\begin{aligned} Z^\textrm{glob}=Z|_{{\textsf{a}}=0}+\mathrm {correction\; terms}, \end{aligned}$$

see Sect. 1.1.5.

#### Mathematical definition of the perturbative partition function

For $A_0$ any flat connection on *M*, adapting the construction of [CM08], one defines the perturbative Chern–Simons partition function as

4 $$\begin{aligned} &  Z_{A_0}({\textsf{a}})=e^{\frac{i}{\hbar }S_{CS}(A_0)} \tau (A_0)^\frac{1}{2} e^{\frac{\pi i}{4}\psi (A_0)} \exp \sum _\Gamma \frac{(-i\hbar )^{-\chi (\Gamma )}}{|\textrm{Aut}(\Gamma )|}\Phi _{\Gamma ,A_0}({\textsf{a}}) \nonumber \\ &  \quad \in \textrm{Dens}^{\frac{1}{2},\textrm{formal}}(H^\bullet _{A_0}[1])=\textrm{Det}^\frac{1}{2}(H^\bullet _{A_0})\otimes \widehat{\textrm{Sym}}(H^\bullet _{A_0}[1])^* \end{aligned}$$

– a formal half-density on de Rham cohomology twisted by $A_0$. Here:

- $H_{A_0}^\bullet$ is the cohomology of the complex of ${\mathfrak {g}}$-valued differential forms on *M* with differential $d_{A_0}=d+\textrm{ad}_{A_0}$. One calls the variable ${\textsf{a}}\in H_{A_0}^\bullet [1]$ the *zero-mode*.
- $\tau (A_0)$ is the Ray-Singer torsion. For $A_0$ non-acyclic, rather than being a number, it is an element of the determinant line of the cohomology $H^\bullet _{A_0}$, see Remark 1.1 below.
- $\psi (A_0)$ is the Atiyah-Patodi-Singer eta-invariant of the operator ${L_-:=*d_{A_0}+d_{A_0}*}$ acting on forms of odd degree.
- The sum ranges over connected 3-valent graphs (“Feynman graphs”) Γ with leaves (loose half-edges) allowed. $\chi (\Gamma )$ is the Euler characteristic of the graph. The weight $\Phi _{\Gamma ,A_0}({\textsf{a}})$ of a graph Γ is a polynomial in ${\textsf{a}}$ with coefficients given by certain integrals over the compactified configuration space of points on *M*, of a form defined in terms of Hodge decomposition data on *M*, defined by a metric on *M* and twisted by the local system $A_0$.

We refer to Sect. 3.1 for full details, in particular for the formula for Feynman weights $\Phi _{\Gamma ,A_0}({\textsf{a}})$.

Some elements of the formula (4) depend on the choice of a Riemann metric on *M* (namely, the eta-invariant and Feynman weights). The dependence of the full object *Z* on metric – with an appropriate renormalization factor included – turns out to be BV-exact, see Sect. 1.4.

#### Aside: BV pushforward perspective

We briefly recall the BV pushforward construction which in particular elucidates:

(i) why one should expect *Z* to be a half-density on the space of zero-modes and
(ii) why one should expect *Z* to change by a BV-exact term when the metric on *M* is deformed.

Recall that in the BV formalism, one has a construction of a BV pushforward, or fiber BV integral^2^: Let

5 $$\begin{aligned} V=V'\times V'' \end{aligned}$$

be a degree (-1)-symplectic manifold (“space of fields”) presented as a product of degree (-1)-symplectic manifolds (“slow/infrared fields” and “fast/ultraviolet fields”) and $\mathcal {L}\subset V''$ be a Lagrangian submanifold. Then one has a BV pushforward map from half-densities on all fields to half-densities on slow fields

6 $$\begin{aligned} P_* {\mathop {=}\limits ^{\textrm{def}}} \textrm{id}_{V'}\otimes \int _{\mathcal {L}}:\quad \textrm{Dens}^{\frac{1}{2}}(V) \rightarrow \textrm{Dens}^{\frac{1}{2}}(V'). \end{aligned}$$

By BV version of Stokes’ theorem [Sch93], one has that

- $P_*$ is a chain map w.r.t. the BV Laplacians on half-densities:
  7 $$\begin{aligned} \Delta ' P_*= P_* \Delta , \end{aligned}$$
  with $$\Delta ,\Delta '$$ the BV Laplacian on the half-densities on *V* and on $V^{\prime }$ respectively.
- Denote the inclusion of $V^{\prime }$ into *V* in the splitting (5) by *i* and the projection of *V* onto $V^{\prime }$ by *p*. Then, for an infinitesimal deformation of *i*, *p* and the Lagrangian $$\mathcal {L}$$, the induced variation of the BV pushforward is $\Delta^{\prime }$-exact,
  8 $$\begin{aligned} \delta _{i,p,\mathcal {L}} \left( P_* \alpha \right) = \Delta 'R, \end{aligned}$$
  for any fixed $\alpha \in \textrm{Dens}^{\frac{1}{2}}(V)$ satisfying $\Delta \alpha =0$, with the generator
  *R* given explicitly in terms of the variation of $i,p,\mathcal {L}$, see [CM08, CMR17].

The two properties of $P_*$ above are a theorem in the finite-dimensional case; for infinite-dimensional BV pushforward (defined via perturbative path integral) they become a heuristic statement—an expectation—that has to be proven independently at the level of Feynman diagrams.

In the example of Chern–Simons theory, restricted to perturbations of a fixed flat connection $$A_0$$, we have $$V=\Omega ^\bullet (M,{\mathfrak {g}})[1]$$ with $V^{\prime }$ being the $$A_0$$-harmonic forms and $V^{\prime \prime }$ their orthogonal complement (w.r.t. the Hodge inner product), with

9 $$\begin{aligned} \mathcal {L}=\textrm{im}(d^*_{A_0}) \end{aligned}$$

being the coexact forms. Then one has a function on *V*,

10 $$\begin{aligned} f(B):= &  S_\textrm{CS}(A_0+B) \nonumber \\= &  S_\textrm{CS}(A_0)+\int _M\textrm{tr}\left( \frac{1}{2} B\wedge d_{A_0} B+\frac{1}{6} B\wedge [B,B]\right) . \end{aligned}$$

As a function of *B* it satisfies the BV classical master equation $\{f,f\}=0$. Denoting by $\mu _0$ the formal translation-invariant half-density on *V*, one has $\Delta (e^{\frac{i}{\hbar }f}\mu _0 )=0$ where the l.h.s. should be appropriately regularized [Cos11]. Then the perturbative partition function (4) is the perturbative evaluation of the BV pushforward

11 $$\begin{aligned} Z= P_*\left( e^{\frac{i}{\hbar }f}\mu _0\right) \end{aligned}$$

for the gauge-fixing Lagrangian $$\mathcal {L} = \textrm{im}(d^*_{A_0})$$—this is the origin of the Chern–Simons path integral (3). In particular, from this viewpoint it is natural that *Z* is a *half-density* (rather than a function) on $V^{\prime }$.

##### Remark 1.1

The free Gaussian part of the BV pushforward (11) is the formal Gaussian integral of $e^{\frac{i}{\hbar } \int _M \textrm{tr}\frac{1}{2} B\wedge d_{A_0} B}$ which is interpreted (as guided by finite-dimensional oscillatory Gaussian integrals) as

12 $$\begin{aligned} ``{({\det }^\textrm{reg} d_{A_0})^\frac{1}{2} e^{\frac{\pi i}{4}\textrm{sign}^\textrm{reg}(d_{A_0})},}" \end{aligned}$$

with “${\det }^\textrm{reg} d_{A_0}$” the gauge-fixed (gauge-fixing can be thought of replacing $d_{A_0}$ with $(d^*_{A_0}d_{A_0})^{\frac{1}{2}}$ and then restricting it to coexact forms) and zeta-regularized determinant of $d_{A_0}$, a.k.a. the Ray-Singer torsion. We refer to [Sch78, Wit89, Mne14, Section 1.7], [CMR20] for details on Ray-Singer torsion $\tau (A_0)$ (or Reidemeister torsion in the combinatorial setting of [CMR20]) and how it appears as a Gaussian integral with kinetic operator given by de Rham operator twisted by a flat connection. The gauge-fixed and zeta-regularized signature of the kinetic operator in (12) is understood as the Atiyah-Patodi-Singer eta-invariant $\psi (A_0)$, see [Wit89].

Ray-Singer torsion is the regularized version of the general algebraic notion of torsion of a cochain complex $C^\bullet$ with inner product. This torsion $\tau (C^\bullet )$ is an element of the determinant line $\textrm{Det}\,C^\bullet \cong \textrm{Det}\,H^\bullet$, with $H^\bullet$ the cohomology of $C^\bullet$, see [Mil66, Mne14]. (In particular, if $C^\bullet$ is acyclic, the torsion is a number.) In the case of $C^\bullet$ being the de Rham complex twisted by a flat connection $A_0$, the torsion becomes the Ray-Singer torsion—an element of the determinant line of twisted de Rham cohomology $H^\bullet _{A_0}$, or, equivalently, a constant density on the graded space $H^\bullet _{A_0}[1]$. Thus, the square root of torsion participating in the perturbative Chern–Simons path integral (4) is a (constant) half-density on the space of zero-modes $H^\bullet _{A_0}[1]$. We refer also to [BZ92, Bra03] for a discussion of Ray-Singer torsion for non-acyclic flat connections.

Note that the property (8) suggests that under a deformation of Riemannian metric on *M*, *Z* should change by a $\Delta^{\prime }$-exact term.

##### Remark 1.2

There is a correction to the expected statement above – a path integral phenomenon, not visible at the level of finite-dimensional integrals: The partition function (4) exhibits anomalous dependence on metric. For an acyclic flat connection $A_0$, at the 1-loop level, as already observed by Witten [Wit89], this is due to the fact that the eta invariant depends on the metric. At higher loop orders this phenomenon is due to contributions from hidden boundary strata of compactified configuration spaces, as observed by Axelrod and Singer [AS91]. One can cancel this dependence on the metric at the cost of “renormalizing” the partition function by multiplying it with a factor that depends on the metric and a framing (trivialization of the tangent bundle of *M*). The resulting renormalized partition function is independent of metric but depends on the framing—this is the well-known *framing anomaly* of Chern–Simons theory.

In the case of non-acyclic $A_0$, as it turns out, one needs to include the same renormalization factor, and then this renormalized *Z* changes under the variation of metric by a BV-exact term. We refer to Sect. 1.4 below for details (see also Appendix B.3).

##### Remark 1.3

Ultimately, the goal of this activity is to compare the perturbative Chern–Simons partition function and the asymptotics of the Reshetikhin-Turaev invariants [RT91]. Experiments in the literature have shown [FG91, Jef92, Roz95] that to this end one needs to do two things:

- Be more careful in the normalization of the path integral measure.^3^
- Refine the framing correction to 2-framings and use the *canonical* 2-framing.^4^

In this paper we will largely ignore these questions and only comment on them briefly in the motivating example in Sect. 1.6.

#### *Z* as a family over the moduli space of flat connections.

Let ${\mathcal {M}}$ be the moduli space of flat *G*-connections on *M* and ${\mathcal {M}}'\subset {\mathcal {M}}$ the smooth irreducible locus.^5^,^6^

The partition function (4) depends only on the gauge equivalence class $[A_0]$ of the flat connection $A_0$, and thus defines a section of the bundle of formal vertical (i.e., fiberwise) half-densities on the graded vector bundle ${\mathbb {T}}{\mathcal {M}}'$ over ${\mathcal {M}}'$, where the fiber of ${\mathbb {T}}{\mathcal {M}}'$ over $[A_0]$ is $H^\bullet _{A_0}[1]$ (in particular, the degree zero part of ${\mathbb {T}}{\mathcal {M}}'$ is the tangent bundle of ${\mathcal {M}}'$):

13 $$\begin{aligned} Z\in \Gamma ({\mathcal {M}}',\textrm{Dens}^{\frac{1}{2},\textrm{formal}}({\mathbb {T}}{\mathcal {M}}')). \end{aligned}$$

We remark that on ${\mathcal {M}}'$:

- The exponential factor in the partition function (4) is 1+O(ħ) (tree Feynman graphs vanish due to smoothness of $[A_0]$); $S_{CS}(A_0)$ and $\psi (A_0)$ are locally constant functions on ${\mathcal {M}}'$.^7^
- By irreducibility of $[A_0]$ and by Poincaré duality, one has $H^0_{A_0}=H^3_{A_0}=0$ and $H^2_{A_0}\cong (H^1_{A_0})^*$. Therefore, vertical half-densities on ${\mathbb {T}}{\mathcal {M}}'$ are naturally identified with vertical 1-densities on the tangent bundle $T{\mathcal {M}}'$ and in turn, using an orientation^8^ on ${\mathcal {M}}'$, with vertical top-degree forms on $T{\mathcal {M}}'$.

#### The global partition function

Restriction of the perturbative partition function (4) to the zero-section of ${\mathbb {T}}{\mathcal {M}}'$ (i.e., setting ${\textsf{a}}=0$) yields the “naive global partition function”

14 $$\begin{aligned} Z^\textrm{glob,naive}_{A_0}=Z_{A_0}({\textsf{a}}=0) \quad \in \textrm{Dens}^{\frac{1}{2}}_{\textrm{base}}(T^*[-1]{\mathcal {M}}')\cong \Omega ^\textrm{top}({\mathcal {M}}') \end{aligned}$$

where $\textrm{Dens}^{\frac{1}{2}}_{\textrm{base}}(T^*[-1]{\mathcal {M}}')$ denotes half-densities on the shifted cotangent bundle that are independent of the fiber coordinates. It is given by the same formula as (4), where the sum over graphs ranges over trivalent graphs without leaves.

One of the main results of this work is that one can modify (14), by adding certain explicit corrections, to

15 $$\begin{aligned} Z^\textrm{glob}_{A_0}= Z^\textrm{glob,naive}_{A_0} (1+O(\hbar )), \end{aligned}$$

in such a way that:

- With the renormalization factor included (as in (26)), $Z^\textrm{glob}$ defines a cohomology class of ${\mathcal {M}}'$, which is *independent of the metric* (Theoremss 1.7/5.17). In particular, if $\{{\mathcal {M}}'_\alpha \}$ are the connected components of ${\mathcal {M}}'$, then the collection $\{\int _{{\mathcal {M}}'_\alpha } Z^\textrm{glob,ren}\}$ of elements of $e^{\frac{ic_\alpha }{\hbar }}{\mathbb {C}}[[\hbar ]]$ is an invariant of a framed 3-manifold, where $c_\alpha =S_{CS}|_{{\mathcal {M}}'_\alpha }$.
- The pullback of $Z^\textrm{glob}$ by the formal exponential map on ${\mathcal {M}}'$ recovers the perturbative partition function (4) up to a BV-exact term (Theorem 1.4 (c)/Corollary 5.15).

The construction of $Z^\textrm{glob}$ is as follows:

- First, one extends the perturbative partition function (4) to a nonhomogeneous form $\widetilde{Z}$ on the moduli space ${\mathcal {M}}'$ with values in $\textrm{Dens}^{\frac{1}{2},\textrm{formal}}(H_{A_0}^\bullet [1])$.
  This extension is constructed by taking the formula (4) and changing the assignment to edges and leaves of a Feynman graph to appropriate objects^9^ valued in $\Omega ^\bullet ({\mathcal {M}}')$.
- Then one constructs $Z^\textrm{glob}$ as
  16 $$\begin{aligned} Z^\textrm{glob}=\left( \sum _{k\ge 0}\frac{(-i\hbar )^k}{k!}\left\langle \frac{\partial }{\partial {\textsf{a}}^2},\frac{\partial }{\partial [\delta A_0]} \right\rangle ^k \widetilde{Z} \right) \Bigg |_{{\textsf{a}}^1=[\delta A_0]=0}. \end{aligned}$$
  Here ${\textsf{a}}^{1,2}$ are the components of ${\textsf{a}}$ in $H^1_{A_0}$, $H^2_{A_0}$.

The term k=0 in (16) is $Z^\textrm{glob,naive}$, and k≥1 terms are the corrections we referred to above. We refer to Sect. 5.4 for details on the construction and properties of $Z^\textrm{glob}$.

### Dependence of the perturbative partition function on the flat connection: horizontality of *Z*, recovering *Z* from $Z^\textrm{glob}$

#### The sum-over-trees formal exponential map on ${\mathcal {M}}'$.

One can define a map

17 $$\begin{aligned} \phi :V \rightarrow {\mathcal {M}}' \end{aligned}$$

where *V* is an open neighborhood of the zero-section of the tangent bundle $T{\mathcal {M}}'$ such that (a) the restriction of ϕ to the zero-section is the identity map ${\mathcal {M}}'\rightarrow {\mathcal {M}}'$ and (b) the vertical component of the differential dϕ on the zero-section is identity. Such a map ϕ is called, in the language of formal geometry, a “formal exponential map.”^10^

One can define a particular formal exponential map ϕ explicitly, as a sum over binary rooted trees (modulo isomorphism) with leaves decorated by ${\textsf{a}}\in T_{[A_0]}{\mathcal {M}}'=H^1_{A_0}$, edges (and the root) decorated by Hodge chain homotopy and vertices decorated by the Lie bracket in $\Omega ^\bullet (M,{\mathfrak {g}})$.

#### Main result 1

##### Theorem 1.4

11

- The perturbative partition function (4) satisfies
  18 $$\begin{aligned} \det (B^\vee )\circ Z_{\phi (A_0,\alpha )}( B({\textsf{a}})) = Z_{A_0}(\alpha +{\textsf{a}})-i\hbar \Delta _{\textsf{a}}R(A_0,\alpha ;{\textsf{a}}) \end{aligned}$$
  for any smooth irreducible flat connection $A_0$.^12^ Here:
  ${\textsf{a}},\alpha \in H^1_{A_0}$ are formal variables;
  ϕ is the sum-over-trees formal exponential map;
  $B:=d^\textrm{vert}\phi |_{(A_0,\alpha )}:H^1_{A_0}\rightarrow H^1_{\phi (A_0,\alpha )}$ is the differential of ϕ in the second argument; the determinant of the dual of *B* is a map between determinant lines $\det (B^\vee )=\wedge ^\textrm{top}B^\vee :\textrm{Det}(H^1_{\phi (A_0,\alpha )})^*\rightarrow \textrm{Det}(H^1_{A_0})^*$;
  $\Delta _{\textsf{a}}= \left\langle \frac{\partial }{\partial {\textsf{a}}^{1}},\frac{\partial }{\partial {\textsf{a}}^{2}}\right\rangle$, with brackets meaning the Poincaré duality pairing, is the BV Laplacian on formal half-densities on the fiber of ${\mathbb {T}}{\mathcal {M}}'$ over $A_0$;
  $R(A_0,\alpha ;{\textsf{a}})$ is some degree -1 formal half-density on the fiber of ${\mathbb {T}}{\mathcal {M}}'$ over $A_0$ (in a family parametrized by α).
- The formal exponential map induces a flat connection $\nabla ^G$ (“Grothendieck connection”) on the bundle of formal fiberwise half-densities on ${\mathbb {T}}{\mathcal {M}}'$, and the perturbative partition function is a horizontal section modulo a $\Delta _{\textsf{a}}$-exact term:
  19 $$\begin{aligned} \nabla ^G Z= -i\hbar \Delta _{\textsf{a}}R_1 \end{aligned}$$
  with some degree -1 generator $R_1\in \Omega ^1({\mathcal {M}}',\textrm{Dens}^{\frac{1}{2},\textrm{formal}}({\mathbb {T}}{\mathcal {M}}'))$.
- Under one can recover *Z* from $Z^\textrm{glob}$ modulo a $\Delta _{\textsf{a}}$-exact term, as
  20 $$\begin{aligned} {\textbf{T}} (\phi ^*)^\textrm{vert} Z^\textrm{glob} = Z+i\hbar \Delta _{\textsf{a}}R_\mathrm {glob-pert}, \end{aligned}$$
  On the left, $(\phi ^*)^\textrm{vert}$ stands for the fiberwise top form on $V\subset T{\mathcal {M}}'$ obtained from the pullback of a top form on ${\mathcal {M}}'$; ${\textbf{T}}$ stands for taking the Taylor expansion in the fiber coordinates on $T{\mathcal {M}}'$. The resulting formal fiberwise top form on $T{\mathcal {M}}'$ is reinterpreted as a formal fiberwise degree zero half-density on ${\mathbb {T}}{\mathcal {M}}'$. $R_\mathrm {glob-pert}$ is some degree -1 formal fiberwise half-density on ${\mathbb {T}}{\mathcal {M}}'$.

Equation (18) expresses the fact (expected from the heuristic formula (3)) that a shift α of the flat connection $A_0$ can be absorbed into a shift of the zero-mode ${\textsf{a}}$, modulo a BV-exact term. Formula (19) expresses the same fact infinitesimally (to first order) in the shift α.

Equation (20) is related to the fact that *Z* can be modified by a BV-exact term to a strictly global object (horizontal w.r.t. $\nabla ^G$), see Theorem 5.10.

The generator $R_1$ in (19) is given explicitly as a sum over graphs with one marked edge or leaf, cf. Corollary 5.9. Generators $R(A_0,\alpha ;{\textsf{a}})$ in (a) and $R_\mathrm {glob-pert}$ in (c) are also given explicitly by (227), (247).

##### Remark 1.5

Cohomology of $\Delta _{\textsf{a}}$ acting on (formal or smooth) half-densities on the odd-symplectic graded vector space $H_{A_0}[1]=T^*[-1]H^1_{A_0}$ is concentrated in ghost number $-\dim H^1_{A_0}$ and has rank one there. This is a consequence of Poincaré lemma, since the odd Fourier transform gives a chain isomorphism $\textrm{Dens}^{\frac{1}{2}}(T^*[-1]V)_{-k},\Delta _{\textsf{a}}\cong \Omega ^{\dim V-k}(V),d$, for $V=H^1_{A_0}$ a vector space. Thus, $H_{\Delta _{\textsf{a}}}^{-k}$ is the de Rham cohomology of a point in degree dimV-k.

Thus, if $\dim H^1_{A_0}>0$ (i.e., $A_0$ is not an isolated point of ${\mathcal {M}}'$), the perturbative partition function *Z* (which is automatically $\Delta _{\textsf{a}}$-closed for degree reason) is in fact $\Delta _{\textsf{a}}$-exact. From this standpoint, statements like (19), saying that something holds for *Z* up to *some* BV-exact term $\Delta _{\textsf{a}}R$ might look trivial, since *Z* is itself BV-exact. What makes these statements nontrivial is that (i) we give a formula for *R*, (ii) the statement holds in a family over ${\mathcal {M}}'$, with a coherent choice of *R*. More precisely, *Z* possesses an extension to a nonhomogeneous form ${\check{Z}}$ on the space of background data, whose 0-from component is *Z* and 1-form component is *R*, satisfying the “differential quantum master equation,” see Sect. 1.5.

### “Desynchronized” Chern–Simons partition function—main result 2

Parts (a), (b) of Theorem 1.4 follow from an auxiliary statement on the “desynchronized” partition function which we explain below.

In the partition function (4), the flat connection $A=A_0$ played two different roles: it was the local system for the kinetic operator $d_{A_0}$ (cf. the quadratic term $B\wedge d_{A_0} B$ in (10)) and it was a parameter in the Lorenz gauge-fixing (9).

One can allow the parameter in the kinetic operator $d_{A}$ and in the gauge-fixing operator $d^*_{A'}$ to be two different (but sufficiently close) flat connections. This leads to the “desynchronized” partition function $Z_{A,A'}({\textsf{a}})$ which is given by the formula (4) with the following modification: weights of Feynman graphs are based on a “desynchronized” analog of Hodge decomposition of forms on *M*, based in turn on the operators $d_{A}$, $d^*_{A'}$.

The desynchronized partition function is still a formal half-density on $$H^\bullet _{A}[1]$$. By construction, it satisfies the “extension property”: the restriction of $$Z_{A,A'}({\textsf{a}})$$ to the diagonal $A=A^{\prime }$ coincides with $$Z_{A}({\textsf{a}})$$,

21 $$\begin{aligned} Z_{A,A}({\textsf{a}}) = Z_{A}({\textsf{a}}). \end{aligned}$$

We denote $\textrm{FC}$ the space of flat connections and $\textrm{FC}'\subset \textrm{FC}$ the subspace of smooth irreducible connections.

Theorem 1.4 above (parts (a) and (b)) is a consequence of the following collection of results on the desynchronized partition function.

#### Theorem 1.6

13 Let $A,A^{\prime }$ be a pair of sufficiently close smooth irreducible flat connections. Then we have:

- *Gauge invariance:* We have that $Z_{A,A'}({\textsf{a}})$ is invariant under “diagonal” gauge transformations $(A,A',{\textsf{a}}) \mapsto ({}^\textrm{g}A,{}^\textrm{g}A',{}^\textrm{g}{\textsf{a}})$.
- *Variation of kinetic operator:* The desynchronized partition function satisfies
  22 $$\begin{aligned} \det (B^\vee ) \circ Z_{\phi (A,A',\alpha ),A'}(B({\textsf{a}})) = Z_{A,A'}(\alpha + {\textsf{a}}) \end{aligned}$$
  with notations as in Theorem 1.4 above; $\phi (A,A',-):H_A^1\rightarrow \textrm{FC}'$ is the desynchronized variant of the sum-over-trees map.
- *Infinitesimal variation of kinetic operator:* The map ϕ induces a partial connection ${\widetilde{\nabla }}_G$ in the direction of harmonic shifts of *A* on the bundle of formal half-densities on $H_{A}[1]$ such that
  23 $$\begin{aligned} {\widetilde{\nabla }}_GZ_{A,A'} = 0. \end{aligned}$$
- *Variation of gauge fixing operator:* We have that, for $A_1'$ sufficiently close to $A'_0$,
  24 $$\begin{aligned} Z_{A,A_1'}({\textsf{a}}) = Z_{A,A'_0}({\textsf{a}}) - i\hbar \Delta _{\textsf{a}}R(A,A'_0,A_1',{\textsf{a}}). \end{aligned}$$
  For an infinitesimal variation of $A'\rightarrow A'+\delta A'$, one has
  25 $$\begin{aligned} \delta _{A'}Z_{A,A'}({\textsf{a}})=-i\hbar \Delta _{\textsf{a}}R_{\delta A'}(A,A',{\textsf{a}}), \end{aligned}$$
  with $R_{\delta A'}$ given by a sum over graphs with one marked edge or one marked leaf (cf. Proposition 4.11).

**Fig. 1:**
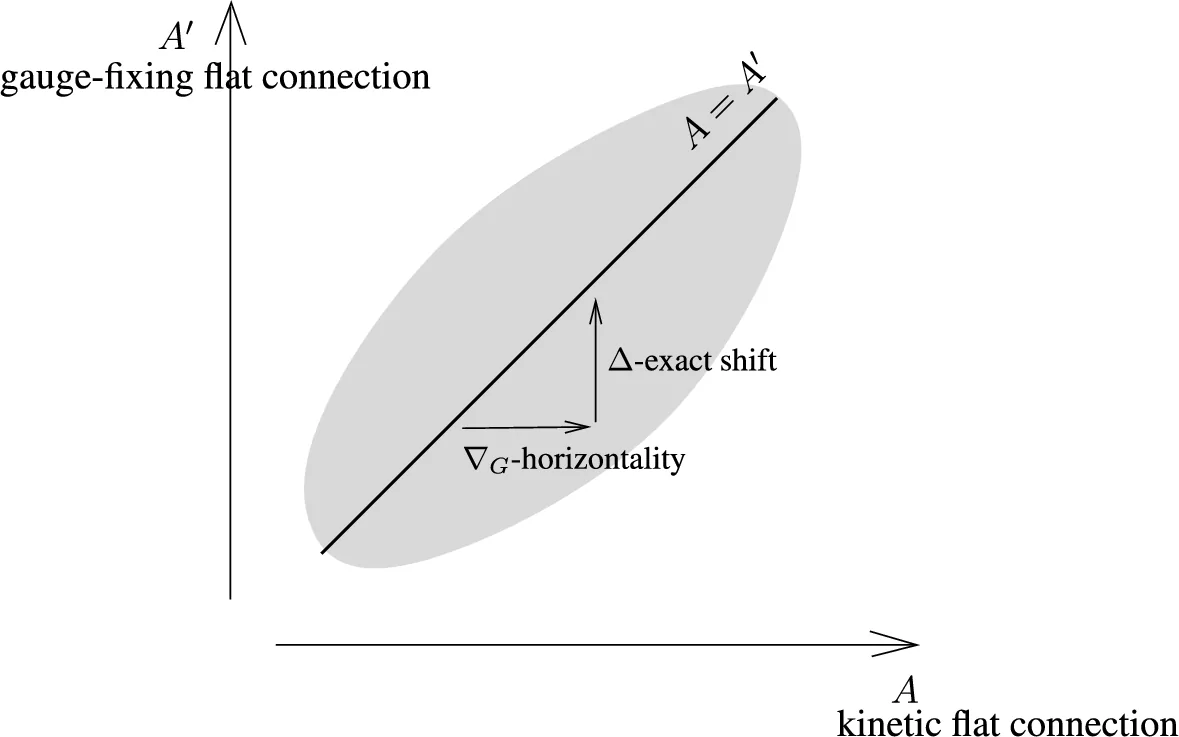
Desynchronized partition function *Z* is a section of the bundle of formal half-densities on $$H_{A}[1]$$ over a neighborhood of the diagonal in $$\textrm{FC}'\times \textrm{FC}'$$. Under harmonic shifts of *A* the section is $$\widetilde{\nabla }^G$$-horizontal, shifts of $A^{\prime }$ change *Z* by a BV-exact term

Parts (a), (b) of Theorem 1.4 follow by setting $A=A^{\prime }$. The original motivation to consider the “desynchronized” partition function was precisely that the shift in the zero mode as on the right hand side of (22) produces a variation in the “kinetic operator” *A* while keeping the gauge fixing operator fixed (Fig. 1).

### Metric (in)dependence of the global partition function—main result 3

For the perturbative partition function, one has the following result [CM08, Mne19, Wer22]:

- The perturbative partition function $Z(A_0,{\textsf{a}})$ is closed with respect to the canonical BV Laplacian on formal half-densities on $H^\bullet _{A_0}[1]$.
- There is a universal power series $c(\hbar ) = \frac{\dim G}{24}\hbar + c'(\hbar ), c'(\hbar ) \in \hbar ^2{\mathbb {R}}[[\hbar ^2]]$ such that for every framing $f:TM \xrightarrow {\cong } M \times {\mathbb {R}}^3$ the “renormalized” partition function
  26 $$\begin{aligned} Z^\textrm{ren}_{A_0}({\textsf{a}}) := e^{\frac{i}{\hbar }c(\hbar )\frac{S_\textrm{grav}(g,f)}{2\pi }}Z_{A_0}({\textsf{a}}) \end{aligned}$$
  is independent of the metric *g*, up to BV-exact terms^14^:
  27 $$\begin{aligned} \delta _g Z^\textrm{ren}_{A_0}({\textsf{a}})=-i\hbar \Delta _{\textsf{a}}R_{\delta g}. \end{aligned}$$
  Here $S_\textrm{grav}$ is the evaluation of the Chern–Simons action on the Levi-Civita connection of *g* via the framing.^15^

For these statements one does not need to assume that $A_0$ is smooth or irreducible. In particular, the BV cohomology class of $Z^\textrm{ren}_{A_0}({\textsf{a}})$ is independent of the metric *g*.^16^

In this paper, we investigate the metric dependence of the “global partition function” $Z^\textrm{glob}$. Since it does not depend on the fiber coordinates, the global partition function $Z^\textrm{glob} \in \textrm{Dens}^{\frac{1}{2}}_\textrm{base}(T^*[-1]{\mathcal {M}}')$ is trivially BV-closed on $T^*[-1]{\mathcal {M}}'$,

28 $$\begin{aligned} \Delta _{{\mathcal {M}}'}Z^\textrm{glob} = 0. \end{aligned}$$

Our main result in this direction is that the BV cohomology class of the renormalized global partition function is independent of the metric used to define the gauge-fixing.

#### Theorem 1.7

17 Suppose $g_t, t\in (-\varepsilon ,\varepsilon )$, is a smooth family of Riemannian metrics on *M*, and denote by $Z_t^\textrm{glob,ren}$ the global partition function defined using the metric $g_t$, renormalized as in (26). Then we have

29 $$\begin{aligned} \frac{d}{dt}\bigg |_{t=0} Z_t^\textrm{glob,ren} = -i\hbar \Delta _{{\mathcal {M}}'}(R^\textrm{glob}), \end{aligned}$$

where $R^\textrm{glob}$ is a degree -1 half-density given explicitly by the 1-form component of (267) along the space of metrics (evaluated on the tangent vector ${\dot{g}}$), see also (273).

Put differently, if we think of the global partition function as a top form on ${\mathcal {M}}'$, then under a change of metric, it changes by an exact form

30 $$\begin{aligned} \frac{d}{dt}\bigg |_{t=0} Z_t^\textrm{glob,ren} = -i\hbar \, d_{{\mathcal {M}}'}R^\textrm{glob}. \end{aligned}$$

Here we interpret the degree -1 half-density $R^{\textrm{glob}}$ as a differential form of degree $\textrm{top} -1$.

### Extension of the partition function to a nonhomogeneous form in $A,A^{\prime },g$—main result 4

In Sect. 4.6 we consider an open set $\underline{\mathcal {U}}=\{(A,A',g)\;|\;A'\mathrm {\;and\;}A\;\textrm{close}\}$ in $\textrm{FC}'\times \textrm{FC}'\times \textrm{Met}$ and construct:

1. A connection^18^$\nabla ^{{\mathbb {H}}}$ on the “cohomology bundle” ${\mathbb {H}}$ over $\underline{\mathcal {U}}$ with fiber $H_A$ and the induced connection $\nabla ^\mathcal {D}$ on the “half-density bundle” $\mathcal {D}$ over $\underline{\mathcal {U}}$ with fiber $\textrm{Dens}^{\frac{1}{2},\textrm{formal}}(H_A[1])$.
2. An “extended” partition function ${\check{Z}}\in \Omega ^{\bullet ,\bullet ,\bullet }(\underline{\mathcal {U}},\mathcal {D})$ (see (188))—a nonhomogeneous form on $\underline{\mathcal {U}}$ valued in $\mathcal {D}$—defined similarly to (4), where the weights of Feynman graphs are extended appropriately to differential forms on $\underline{\mathcal {U}}$; we also include the framing-dependent renormalization factor as in (26).

#### Theorem 1.8

19 The extended partition function satisfies the following “differential quantum master equation” (dQME):

31 $$\begin{aligned} (\nabla ^\mathcal {D}-i\hbar \Delta _{\textsf{a}}-\frac{i}{\hbar }\frac{1}{2} \langle {\textsf{a}}, F {\textsf{a}}\rangle ) {\check{Z}} =0. \end{aligned}$$

Here *F* is the curvature of $\nabla ^{\mathbb {H}}$. The expression in brackets acting on ${\check{Z}}$ above is a *flat* superconnection concentrated in de Rham degrees 0,1,2 along $\underline{\mathcal {U}}$. By abuse of terminology, we call it the *Gauss-Manin superconnection*.^20^

Low-degree components of ${\check{Z}}$ and of the dQME (31) yield various objects and infinitesimal variation statements we have encountered in the earlier subsections:

- Degree (0, 0, 0) component of ${\check{Z}}$ is the desynchronized partition function $Z_{A,A'}({\textsf{a}})$.^21^
- Degree (0, 1, 0) component of ${\check{Z}}$ is the generator $R_{\delta A'}$ appearing in (25). Degree (0, 1, 0) component of the dQME is the equation (25).
- Degree (0, 0, 1) component of $${\check{Z}}$$, evaluated at $A^{\prime }=A$, is the generator $$R_{\delta g}$$ in (27); the corresponding component of the dQME is the Eq. (27).
- (1, 0, 0) component of the dQME, contracted with a tangent vector to $\underline{\mathcal {U}}$ representing a harmonic shift of *A*, is equivalent to horizontality w.r.t. partial Grothendieck connection $\widetilde{\nabla }^G$ (23).
- Restricting dQME to the diagonal $A^{\prime }=A$ and fixing *g* and then taking the degree 1 component in *A* yields (19).

Thus, the dQME (31) is an “omnibus equation” implying as low-degree specializations all the infinitesimal variation statements from before.

The restriction of $${\check{Z}}$$ to the diagonal $A=A^{\prime }$ is the key ingredient in the construction of the global partition function, see Sect. 5.4.

### Motivating example: $\Sigma \times S^1$

As a motivating example where the (leading) asymptotics of the Reshetikhin-Turaev invariants agree with the integral of $Z^\textrm{glob}$ over the moduli space of flat connections, consider the case $M = \Sigma \times S^1$, with Σ a Riemann surface of genus $\gamma \ge 2$. Then the non-perturbative Chern–Simons partition function—the RT invariant—at level *k* for G=SU(2) is given by the Riemann-Roch-Hirzebruch formula as

32 $$\begin{aligned} Z^{(k)}= &  \dim H^0({\mathcal {M}}(\Sigma )),{\mathcal {L}}^{\otimes k}) = \int _{{\mathcal {M}}'(\Sigma )}e^{k\omega _{AB}}\textrm{Td} (T{\mathcal {M}}) \nonumber \\= &  k^N \int _{{\mathcal {M}}'(\Sigma )} \frac{w_{AB}^N}{N!}+O(k^{N-1}) \end{aligned}$$

where $\omega _{AB}$ denotes the Atiyah-Bott symplectic form on ${\mathcal {M}}(\Sigma )$, ${\mathcal {M}}'(\Sigma )$ denotes the subset corresponding to irreducible flat connections,^22^$\textrm{Td}$ the Todd class and $N = \frac{1}{2}\dim {\mathcal {M}}(\Sigma )$. By work of Witten [Wit91], the symplectic volume is related to the torsion as

33 $$\begin{aligned} k^N \int _{{\mathcal {M}}'(\Sigma )} \frac{w_{AB}^N}{N!} = \frac{k^N}{(2\pi )^{2N}}\int _{{\mathcal {M}}'(\Sigma )}\tau _\Sigma . \end{aligned}$$

We want to compare this with the integral

$$\begin{aligned} Z^\textrm{num} = \int _{{\mathcal {M}}'(\Sigma \times S^1)} Z^\textrm{glob} \end{aligned}$$

– the number-valued partition function. Let $A_0$ be an irreducible flat connection on $\Sigma \times S^1$. Then $A_0$ is gauge equivalent to a connection of the form

34 $$\begin{aligned} A_0 = \pi ^*\phi \, dt + \pi ^*\alpha \end{aligned}$$

where α is a flat connection on Σ and $\phi \in \Omega ^0(\Sigma ,{\mathfrak {g}})$ is $d_{A_0}$-closed. In particular, all irreducible connections are smooth and there is a bijection (in fact a diffeomorphism)

35 $$\begin{aligned} {\mathcal {M}}'(\Sigma \times S^1) \rightarrow \bigsqcup _{g \in Z(G)} {\mathcal {M}}'(\Sigma ) \end{aligned}$$

which sends the class of $\pi ^*\phi \, dt + \pi ^*\alpha$ to the pair ([α],g) where g∈G is the holonomy of $A_0$ along the circle direction, if α is irreducible, then *g* is necessarily central.

For flat connections of the form (34), we have that $S_{CS}(A_0)=0$.^23^ This also implies that $\psi (A_0,g) = 0$.^24^ Under the identification (35) we have $\tau _{\Sigma \times S^1} = \tau _\Sigma ^2$ and therefore we get

36 $$\begin{aligned} \int _{{\mathcal {M}}'(\Sigma \times S^1)}Z^\textrm{glob}= &  \int _{{\mathcal {M}}'(\Sigma \times S^1)}\tau _{\Sigma \times S^1}^\frac{1}{2}(1 + O(\hbar )) \nonumber \\= &  |Z(G)|\int _{{\mathcal {M}}'(\Sigma )}\tau _\Sigma (1 + O(\hbar )). \end{aligned}$$

Equations (32) and (36) agree if we identify $k = \frac{2\pi }{\hbar }$ and divide $Z^\textrm{glob}$ by $|Z(G)|(2\pi \hbar )^{N}$. In particular, here the framing correction vanishes in the canonical 2-framing. This is precisely the factor we were alluding to in Remark 1.3 and agrees with the proposals in the literature such as [FG91, Roz95, Res10].

### Comparison to literature and historical remarks

The problem of studying the perturbative (or semiclassical) behavior of the Chern–Simons partition function around non-acyclic flat connections, where the path integral has zero modes, was already observed in Witten’s seminal paper on the subject [Wit89, p.361]. Axelrod and Singer studied the perturbative theory around acylic flat connections in detail [AS91, AS94] but already comment that the assumption on acyclicity should be removed, and state (without proof) that the partition function changes by a total divergence when changing the Riemannian metric. They also identify the problem of defining the integral over the moduli space and proving that it is finite, as well as potential anomalies. Axelrod has a later text in conference proceedings on the subject [Axe95], where he develops the theory of oscillatory integrals of Morse-Bott functions and announces some theorems on their application to Chern–Simons theory, but without proof. Our work is independent from Axelrod’s paper^25^ and draws on a different background - BV pushforwards.

The main body of the literature on perturbative Chern–Simons theory turned to the study of (rational) homology 3-spheres, where one can treat the problem of zero modes either by puncturing [Kon93b, KT99, Les02] (resulting in the Kontsevich-Kuperberg-Thurston-Lescop or KKTL invariant) or by introducing extra vertices as in the works of Bott and Cattaneo [BC98, BC99] to cancel the effect of zero modes.^26^ Cattaneo later showed those constructions agree [Cat99]. Another line of research focused on extracting perturbative invariants of 3-manifolds from the Kontsevich integral [Kon93a], such as the Aarhus integral [BGRT02a, BGRT02b] and the LMO invariant [LMO98].^27^ A full definition of the perturbative Chern–Simons partition function at non-acyclic flat connections only appeared with the introduction of the BV formalism to the problem and the works of Cattaneo and the first author [CM08] (see also [Mne19, Wer22]) and simultaneously Iacovino [Iac08].

In the present paper we show how to use the BV partition function to define a volume form on smooth components of the moduli space whose cohomology class is a topological invariant of the framed 3-manifold. We defer to future work the question of anomalies and convergence of the integral over noncompact smooth components, as well as a more detailed study of the behavior at singular points.

## Formal Geometry on the Moduli Space of Flat Connections

In this section we discuss formal geometry on the moduli space of flat connections on a trivialized principal *G*-bundle P=M×G over a 3-manifold *M*. We will assume that *G* is a compact, simple and simply connected matrix group, such as G=SU(n), and denote ${\mathfrak {g}}$ its Lie algebra. In particular, we discuss two special types of points in the moduli space, *smooth* and *irreducible points*. Roughly speaking, smooth points are the ones where all obstructions to deformations vanish, while irreducible points are those with a minimal stabilizer, so that one can ignore stacky aspects of the moduli space.

### Smooth points

In this subsection we specialize the results and definitions of [CMR14, Appendix C] to the case of Chern–Simons theory and its Euler-Lagrange moduli space, the moduli space of flat connections.

Since *P* is trivialized, we identify connections on *P* with their connection 1-forms $\textrm{Conn}(P) \cong \Omega ^1(M,{\mathfrak {g}})$, and denote

37 $$\begin{aligned} \textrm{FC}\equiv \textrm{FC}(P) = \{A \in \Omega ^1(M,{\mathfrak {g}}) \;|\; dA + \frac{1}{2}[A,A] = 0 \} \subset \Omega ^1(M,{\mathfrak {g}}) \end{aligned}$$

the space of flat connections on *P*. We also identify $\textrm{Aut}\,P \cong C^\infty (M,G)$, its action on $\textrm{Conn}(P)$ is given by

$$\begin{aligned} \textrm{g}\cdot A \equiv ^\textrm{g}A \equiv \textrm{g}A\textrm{g}^{-1} + \textrm{g}d \textrm{g}^{-1}. \end{aligned}$$

The moduli space of flat connections is

38 $$\begin{aligned} {\mathcal {M}}\equiv {\mathcal {M}}(M,P) = \textrm{FC}/\operatorname {Aut}P. \end{aligned}$$

**Fig. 2:**
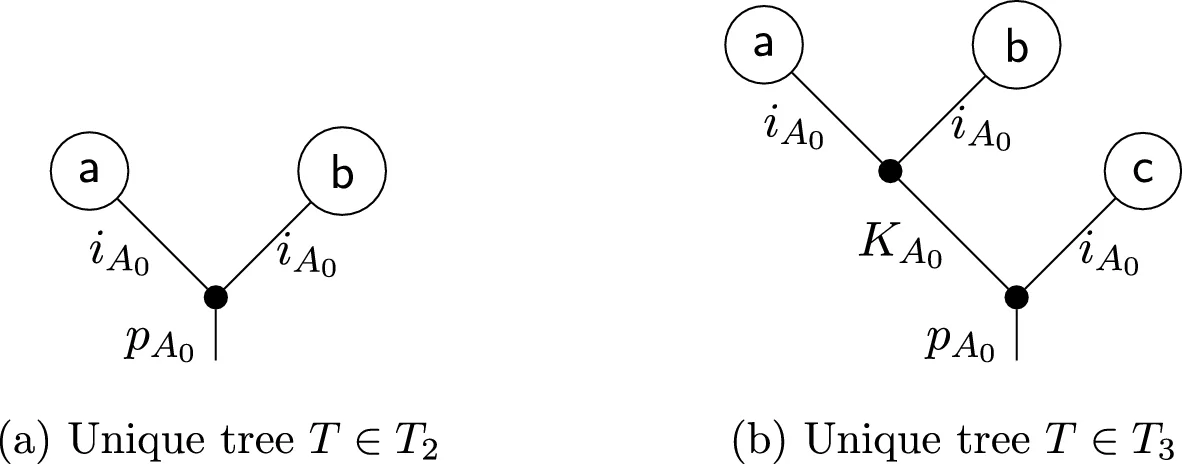
Trees with labeling defining $\lambda _T$

Next, we turn to the definition of smooth points in $\textrm{FC}$ and ${\mathcal {M}}$. Let $A_0 \in \textrm{FC}$ be a flat connection on *P*. Then $\Omega ^\bullet (M,\textrm{Ad}P) \cong \Omega ^\bullet (M,{\mathfrak {g}})$ carries the structure of a differential graded Lie algebra with differential the twisted de Rham differential $d_{A_0} = d+ [A_0,\cdot ]$ and Lie bracket the extension of the Lie bracket on ${\mathfrak {g}}$ to differential forms. We denote this dgla by $\Omega ^\bullet _{A_0} = (\Omega ^\bullet (M,{\mathfrak {g}}), d_{A_0})$ and by

$$\begin{aligned} H^\bullet _{A_0} := H^\bullet _{d_{A_0}}(M,{\mathfrak {g}}) \end{aligned}$$

the cohomology of $d_{A_0}$. By homotopy transfer of $L_\infty$-algebras, $H^\bullet _{A_0}$ is turned into a minimal $L_\infty$-algebra endowed with induced operations $\{l'_{n,A_0}\}_{n\ge 2}$. A choice of SDR data^28^$r_{A_0} = (i_{A_0},p_{A_0},K_{A_0})$ of $\Omega ^\bullet _{A_0}$ onto $H^\bullet _{A_0}$ provides us with explicit representatives of these operations (see Appendix A for our conventions on SDR data). Denote $T_n$ the set of isomorphism classes of binary rooted trees with *n* leaves—here we think of leaves and the root as half-edges emanating from internal vertices. To $T\in T_n$ we can associate an *n*-ary operation $\lambda _T:\wedge ^n H^\bullet _{A_0} \rightarrow H^\bullet _{A_0}$ of degree 2-n as follows: To the *n* leaves we assign the map $i_{A_0}$, to internal vertices we assign the map $l_2$, to internal edges we assign the map $K_{A_0}$, to the root we assign the map $p_{A_0}$ (see Fig. 2); then we skew-symmetrize over the permutations of *n* inputs on the *n* leaves. Then we have

39 $$\begin{aligned} l_{n,A_0}'= (-1)^n\sum _{T \in T_n}{ \frac{ n!}{|\operatorname {Aut} T|}}\lambda _T. \end{aligned}$$

Explicitly, the first two non-vanishing operations are given by

$$\begin{aligned} l'_{2,A_0}({\textsf{a}},{\textsf{b}})&= p_{A_0}[i_{A_0}({\textsf{a}}),i_{A_0}({\textsf{b}})], \\ l'_{3,A_0}({\textsf{a}},{\textsf{b}},{\textsf{c}})&= {\textrm{Sym}_{{\textsf{a}},{\textsf{b}},{\textsf{c}}}}\Big ( p_{A_0}[-K_{A_0}[i_{A_0}({\textsf{a}}),i_{A_0}({\textsf{b}})],i_{A_0}({\textsf{c}})] \Big ). \end{aligned}$$

Here $\textrm{Sym}_{{\textsf{a}},{\textsf{b}},{\textsf{c}}}$ stands for skew-symmetrization in ${\textsf{a}},{\textsf{b}},{\textsf{c}}$.

#### Definition 2.1

*(*[CMR14]*)*. We say that $A_0$ is smooth if, for all n≥0, we have $l'_n = 0$.

We denote by $\textrm{FC}^\textrm{sm} \subset \textrm{FC}$ the subset of all smooth flat connections.

The purpose of this subsection is to prove that both the space of all flat connections and the moduli space of flat connections are smooth manifolds close to a smooth point $A_0$, resp. its class in the moduli space $[A_0]$. It follows that $\textrm{FC}^\textrm{sm}$ and ${\mathcal {M}}^\textrm{sm} = \pi (\textrm{FC}^\textrm{sm})$ are smooth manifolds.^29^

#### Remark 2.2

*(Topologies on* $\Omega ^\bullet$ *and* $\textrm{FC}$*)* The vector space $\Omega ^\bullet$ carries the natural Fréchet topology of uniform convergence, with respect to which it is complete. Moreover, it also carries the coarser Banach topologies induced by the Sobolev norms^30^$||\cdot ||_{l,p}$. The spaces $\textrm{FC}' \subset \textrm{FC}^\textrm{sm} \subset \textrm{FC}\subset \Omega ^1$ carry the corresponding subset topologies. With respect to the $||\cdot ||_{l,p}$ norm none of those spaces is complete and we denote the completion by $\Omega ^\bullet _{l,p}, \textrm{FC}_{l,p},\ldots$. We usually work with p=2 and denote $\Omega ^\bullet _{l,2} = \Omega _{l}$.^31^

Given the different topologies, one can make different assumptions on $K_{A_0}$.

#### Assumption 2.3

*(l-Sobolev boundedness)*. The chain homotopy $K_{A_0}:\Omega ^\bullet \rightarrow \Omega ^{\bullet -1}$ is bounded with respect to the Sobolev norm $||\cdot ||_{l,2}$.

Actually, the main example considered in this text, the SDR coming from Hodge decomposition (Example 2.30) satisfies the following stronger assumption:

#### Assumption 2.4

*(Fréchet continuity)*. The chain homotopy $K_{A_0} :\Omega ^\bullet \rightarrow \Omega ^{\bullet -1}$ is continuous in the Fréchet topology.

The goal of this subsection is to prove the following theorems:

#### Theorem 2.5

For every l≥2, the completion of the set of smooth points $\textrm{FC}^\textrm{sm}_l \subset \textrm{FC}_l$ has the structure of a Banach manifold. For every smooth point $A_0$, there is a neighborhood $V_{A_0}$ of $A_0 \in \textrm{FC}_l$ modeled on a neighborhood $U_{A_0}$ of $0 \in (\Omega ^1_{A_0-\textrm{cl},l},||\cdot ||_l)$. Given SDR data $r_{A_0}$ at $A_0$ satisfying Assumption 2.3, we have a homeomorphism in the Banach topologies

40 $$\begin{aligned} \widetilde{\varphi }_{A_0} :U_{A_0} \rightarrow V_{A_0}, \qquad \alpha \mapsto \widetilde{\varphi }_{A_0}(\alpha ) = A_0 + \sum _{k\ge 0}\alpha ^{(k)} \end{aligned}$$

with $\alpha ^{(1)} = \alpha$ and $\alpha ^{(k)}$ given by

41 $$\begin{aligned} \alpha ^{(k)} = (-1)^{k-1} \sum _{T \in T_k} \widetilde{\mu }_{T}(\alpha ,\ldots ,\alpha ) \end{aligned}$$

where $\widetilde{\mu }_T$ is defined in Eq. (50) below.

The chart $\widetilde{\varphi }$ is constructed using the chain homotopy $K_{A_0}$. If $K_{A_0}$ is Fréchet continuous, then $\widetilde{\varphi }_{A_0}$ is actually a chart for the Fréchet manifold $\textrm{FC}^\textrm{sm}$.

#### Theorem 2.6

The set $\textrm{FC}^\textrm{sm} \subset \textrm{FC}$ has the structure of a Fréchet manifold. If the chain homotopy $K_{A_0}$ satisfies Assumption 2.4, then the map (40), restricted to $U_{A_0} \cap \Omega ^1_{d_{A_0}-\textrm{cl}}$, is a Fréchet homeomorphism.

In the later sections of the text, we consider $\textrm{FC}' \subset \textrm{FC}^\textrm{sm}$, with this Fréchet manifold structure.

#### Outline of the proof

We briefly outline the strategy of the proof. The Banach case was inspired by [HWW].

1. We define the formal power series $\delta _{A_0} = \sum _{k \ge 0} \alpha ^{(k)}$ as a sum over trees, and show that $A_0 + \delta _{A_0}$ is flat for $\alpha ^{(1)}$ *harmonic*—Proposition 2.7.
2. We show that $\widetilde{\delta }_{A_0}$ (defined using the sum over trees for all closed forms) converges in the Banach topology under Assumption 2.3 (Proposition 2.9) or in the Fréchet topology under Assumption 2.4 (Remark 2.10).
3. We define the Kuranishi map (60), show that it is invertible on $\Omega ^1$ and a compositional inverse of $\delta _{A_0}$ on $\Omega ^1$, and show that it maps Maurer-Cartan forms to closed forms (Proposition 2.12).
4. We establish the Banach smoothness of $\textrm{FC}^\textrm{sm}$ by means of the chart $\widetilde{\psi }$, see Proposition 2.13.
5. Now, one can use the inverse function theorem for $\widetilde{\kappa }$ on $\textrm{FC}^\textrm{sm}$ to conclude that $\widetilde{\kappa }$, restricted to the Maurer-Cartan set, surjects onto a neighborhood of $0 \in \Omega ^1_{cl,l}$, this then finally implies that $\widetilde{\delta }$ lands in the Maurer-Cartan set (Proposition 2.14). This proves Theorem 2.5.
6. Since $\widetilde{\kappa }, \widetilde{\delta }$ are both continuous in the Fréchet topology if $K_{A_0}$ is, restricting them to $\textrm{FC}^\textrm{sm}$ proves Theorem 2.6.

#### Formal deformations of flat connections

We recall the following elementary facts about flat connections. If $A_t :(-\epsilon ,\epsilon ) \rightarrow \Omega ^1$ is a smooth curve of flat connections, then from differentiating $F_{A_t} = 0$ we obtain $d_{A_0}{\dot{A}}_0 =0$, i.e., tangent vectors at $A_0$ to curves of flat connections are $d_{A_0}$-closed 1-forms. If $\textrm{g}_t:(-\epsilon ,\epsilon ) \rightarrow C^\infty (M,G)$ is a curve with $\textrm{g}_0 \equiv 1$ and ${\dot{\textrm{g}}}_0 = \gamma \in \Omega ^0(M, {\mathfrak {g}})$, then the tangent vector at 0 to the curve of flat connections $A_t = {}^{\textrm{g}_t}A_0$ is ${\dot{A}}_0 = -d_{A_0}\gamma$, i.e. the tangent vector to a curve along the gauge orbit of a flat connection is a $d_{A_0}$-exact 1-form. This is equivalent to saying that infinitesimal^32^ deformations of flat connections are $d_{A_0}$-closed 1-forms, and two such deformations are equivalent whenever they differ by an exact 1-form, i.e. equivalence classes of infinitesimal deformations are in 1-to-1 correspondence with the first twisted cohomology group $H^1_{{A_0}}$ (sometimes called the *Zariski tangent space* to the moduli space of flat connections at $[A_0]$).

##### Proposition 2.7

Let $A_0$ be a flat connection on *P*, and let $(i_{A_0},p_{A_0},K_{A_0})$ be SDR data at $A_0$. If $A_0$ is smooth, then all infinitesimal deformations of $A_0$ lift to formal deformations of $[A_0]$, i.e., for every ${\textsf{a}}\in H^1_{A_0}$ there exists a formal power series

42 $$\begin{aligned} \delta = \delta _{A_0}(t{\textsf{a}}) = \sum _{n\ge 1}t^n \alpha ^{(n)} \in \Omega ^1[[t]]\end{aligned}$$

with $\alpha ^{(1)} = i_{A_0}{\textsf{a}}$, such that $A_t:=A_0 + \delta _{A_0}(t{\textsf{a}})$ is flat, i.e. it satisfies

$$\begin{aligned} dA_t + \frac{1}{2} [A_t,A_t] =0 \end{aligned}$$

with $d, [\cdot ,\cdot ]$ the induced operations on $\Omega ^1[[t]]$. Moreover, we have $K_{A_0}\delta _{A_0}(t{\textsf{a}}) = 0$.

##### Proof

We can expand the flatness equation $dA_t + \frac{1}{2}[A_t,A_t] = 0$ in powers of *t*, obtaining

43 $$\begin{aligned}&dA_0 + \frac{1}{2}[A_0,A_0] = 0, \end{aligned}$$

44 $$\begin{aligned}&d_{A_0} \alpha ^{(1)} = 0, \end{aligned}$$

45 $$\begin{aligned}&d_{A_0}\alpha ^{(2)} + \frac{1}{2}[\alpha ^{(1)},\alpha ^{(1)}] = 0, \end{aligned}$$

46 $$\begin{aligned}&\vdots \nonumber \\&d_{A_0}\alpha ^{(n)} + \frac{1}{2}\sum _{k=1}^{n-1}[\alpha ^{(k)},\alpha ^{(n-k)}] = 0. \end{aligned}$$

The first two equations are satisfied by our assumptions. It is instructive to look at the third equation in detail. We see that we can solve it for $\alpha ^{(2)}$ if and only $\frac{1}{2}[\alpha ^{(1)},\alpha ^{(1)}]$ is $d_{A_0}$-exact. Because $\alpha ^{(1)} = \alpha$ is closed by assumption, and the bracket is compatible with the differential, $\frac{1}{2}[\alpha ,\alpha ]$ is always $d_{A_0}$ closed. It is exact if and only if $l_2'({\textsf{a}},{\textsf{a}}) = 0$. In this case, we can write down an explicit solution:

$$\begin{aligned} \alpha ^{(2)} = -\frac{1}{2}K_{A_0}[\alpha ,\alpha ]. \end{aligned}$$

Indeed,

$$\begin{aligned} d_{A_0}\alpha ^{(2)} =-\frac{1}{2}d_{A_0}K_{A_0}[\alpha ,\alpha ] = -\frac{1}{2}(\textrm{id} - P_{A_0} - K_{A_0}d_{A_0})[\alpha ,\alpha ] \end{aligned}$$

and $d_{A_0}[\alpha ,\alpha ] = P_{A_0}[\alpha ,\alpha ] = 0$ by $d_{A_0}$-closedness and vanishing of $l_2$ respectively. The rest of the proof now follows by induction. Suppose we are given $\alpha ^{(1)},\ldots ,\alpha ^{(n-1)}$ satisfying

$$\begin{aligned} d_{A_0}\alpha ^{(k)} = -\frac{1}{2}\sum _{j=1}^{k-1}[\alpha ^{(j)},\alpha ^{(k-j)}] \end{aligned}$$

for k=1,…,n-1 and we are looking for $\alpha ^{(n)}$ to solve (46). Then it is a straightforward application of the Jacobi identity that the right hand side is $d_{A_0}$-closed, and is exact if and only if $l_{n}' = 0$, in which case we can define

$$\begin{aligned} \alpha ^{(n)} = -\frac{1}{2}K_{A_0}\sum _{k=1}^{n-1}[\alpha ^{(k)},\alpha ^{(n-k)}]. \end{aligned}$$

Notice that by construction $K_{A_0}\alpha ^{(k)} = 0$ for k≥0. Therefore $K_{A_0}\delta = 0$ if and only if $K_{A_0}\alpha = 0$. □

We denote the corresponding map by

47 $$\begin{aligned} \varphi _{A_0}:H^1_{A_0} \rightarrow \Omega ^1[[t]],\quad (A_0,{\textsf{a}}) \mapsto \varphi _{A_0}(t{\textsf{a}})= A_0 +\delta _{A_0} (t{\textsf{a}}) . \end{aligned}$$

In fact, we can extract from the proof the following slightly more precise fact:

##### Proposition 2.8

**Fig. 3:**
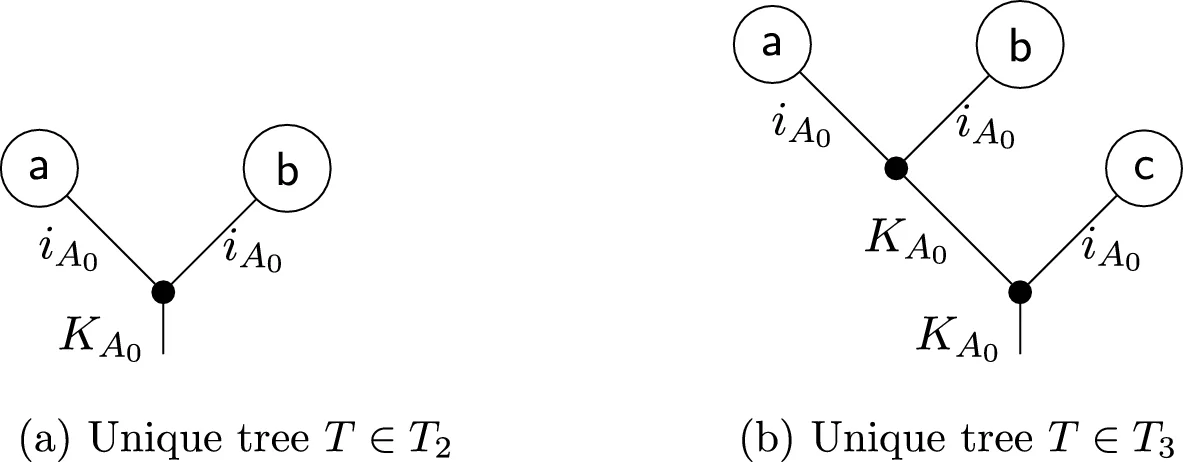
Trees with the labeling defining $\mu _T$

Let $A_0$ be a flat connection (not necessarily smooth), ${\textsf{a}}\in H^1_{{A_0}}$ and k≥2 an integer. Then ${\textsf{a}}$ can be lifted to an order *k* deformation $t\alpha ^{(1)} + \ldots + t^k\alpha ^{(k)}$ if and only if $l_2'({\textsf{a}},{\textsf{a}}) = \ldots =l_k'({\textsf{a}},\ldots ,{\textsf{a}}) = 0$, and in this case

48 $$\begin{aligned} \alpha ^{(n)} ={ (-1)^{n-1}} \sum _{T \in T_n}{ \frac{1}{|\operatorname {Aut} T|}}\mu _T({\textsf{a}},\ldots ,{\textsf{a}})\end{aligned}$$

for $1\le n \le k$. Here the sum is over isomophism classes of rooted binary trees *T* with *n* leaves. The *n*-ary multilinear operation $\mu _T :\textrm{Sym}^n H^\bullet _{A_0}[1] \rightarrow \Omega ^\bullet [1]$ is the evaluation of the tree *T* by putting inputs on the leaves, $l_2$ on internal vertices, $K_{A_0}$ on the internal edges and $K_{A_0}$ on the root, and symmetrizing over inputs, see Fig. 3.

In words, the induced $L_\infty$-operations $l_{n,A_0}'$ on $H^\bullet _{A_0}$ are precisely the obstructions to the lift of infinitesimal deformations to higher-order deformations. At smooth points all those obstructions vanish, so all infinitesimal deformations lift to formal ones.

#### Lifting to forms

For any binary tree *T* with *n* leaves, we can lift the operations $\lambda _T:\textrm{Sym}^n H^\bullet _{A_0}[1] \rightarrow H^\bullet _{A_0}[2]$ and $\mu _T:\textrm{Sym}^n H^\bullet _{A_0}[1] \rightarrow \Omega ^\bullet [1]$ to operations

49 $$\begin{aligned} \widetilde{\lambda }_T&:\textrm{Sym}^n \Omega ^\bullet [1] \rightarrow \Omega ^\bullet [2], \end{aligned}$$

50 $$\begin{aligned} \widetilde{\mu }_T&:\textrm{Sym}^n \Omega ^\bullet [1] \rightarrow \Omega ^\bullet [1] \end{aligned}$$

by replacing $i_{A_0}$ and $p_{A_0}$ with $\textrm{id}_{\Omega ^\bullet }$. Obviously, we have $\lambda _T = p_{A_0} \circ \widetilde{\lambda }_T \circ i_{A_0}^{\otimes n}$ and $\mu _T = \widetilde{\mu }_T \circ i_{A_0}^{\otimes n}$. We have defined the map

$$\delta _{A_0} = t\cdot i_{A_0} + \sum _{k \ge 2} t^k \sum _{T \in T_k} (-1)^{k-1} \mu _T \circ (-)^{\otimes k}:H^1_{A_0} \rightarrow t\Omega ^1[[t]],$$

but it is clear from the definition that it factors through a map

51 $$\begin{aligned} \widetilde{\delta }_{A_0} = t\cdot \textrm{id}_{\Omega ^\bullet } + \sum _{k \ge 2} t^k \sum _{T \in T_k} (-1)^{k-1} \widetilde{\mu }_T \circ (-)^{\otimes k} :\Omega ^1 \rightarrow t \Omega ^1[[t]] \end{aligned}$$

by $$\delta _{A_0} = \widetilde{\delta }_{A_0} \circ i_{A_0}.$$

We now establish by defining another lift of α to a formal deformation. Namely, we split a closed 1-form α as $\alpha = i_{A_0}p_{A_0}\alpha + d_{A_0}K_{A_0}\alpha$. Denoting $\beta = K_{A_0}\alpha$, we can lift α to a (formal) curve of flat connections with tangent vector ${\dot{A}}_0 = \alpha$ by setting $\textrm{g}_{t} = \exp (- t\beta )$ and setting

52 $$\begin{aligned} \widetilde{\psi }_{A_0}(t\alpha ) = {}^{\textrm{g}_t}\varphi _{A_0}(t[\alpha ]) = \textrm{g}_t {\varphi }_{A_0}(t[\alpha ])\textrm{g}_t^{-1} + \textrm{g}_t d \textrm{g}_t^{-1} \in \Omega ^1[[t]]. \end{aligned}$$

Notice that $\varphi _{A_0}(t[\alpha ])$ is flat and therefore $\widetilde{\psi }_{A_0}(t\alpha )$ is also. Expanding $\widetilde{\psi }_{A_0}(t\alpha )$ in powers of *t*, we obtain

53 $$\begin{aligned} \widetilde{\psi }_{A_0}(t\alpha ) = A_0 + \sum _{k\ge 1}t^k\left( \frac{ (-1)^{k-1} }{k!}\textrm{ad}_\beta ^{k-1} d_{A_0}\beta +\sum _{j+l = k, j\ge 0, l\ge 1} \frac{ (-1)^{j} }{j!}\textrm{ad}_{\beta }^j \alpha _h^{(l)}\right) \end{aligned}$$

with $\alpha _h^{(l)}$ as in Proposition 2.7 applied to ${\textsf{a}}=p_{A_0}\alpha$. In this way, we have constructed the map $\widetilde{\psi }_{A_0}(t\alpha ) :\Omega ^1_{A_0-\textrm{cl}} \rightarrow \Omega ^1[[t]]$ lifting any infinitesimal deformation of $A_0$, i.e. a closed form, to a formal deformation.

#### Convergence in Banach norm

It is natural to ask whether the formal deformations defined in the previous sections actually converge. One instance where this happens is the case when $K_{A_0}$ is continuous with respect to a Banach norm on $\Omega ^\bullet$.

##### Proposition 2.9

Suppose $K_{A_0}$ is bounded with respect to $||\cdot ||_{l}$ for some l≥2 on $\Omega ^\bullet$ (Assumption 2.3). Then there is an open interval *I* around zero such that $\widetilde{\delta }_{A_0}$ defined by (51), seen as a formal power series in *t*, converges in $||\cdot ||_l$ for t∈I.

Notice that $i_{A_0}$ and $p_{A_0}$ are automatically bounded, since we are assuming that *M* is compact which implies that $H^1_{A_0}$ is finite-dimensional.

##### Proof

The summand $\alpha ^{(n)}$ is a sum over binary trees with *n* leaves, involving at most *n* applications of $K_{A_0}$. The number of such binary trees is given the (n-1)-th Catalan number, and by assumption $K_{A_0}$ is bounded in $||\cdot ||_l$, say with constant *L*. The Lie bracket $[\alpha ,\alpha ]$ is bounded by

54 $$\begin{aligned} ||[\alpha ,\alpha ]||_l \le F ||\alpha ||^2_l \end{aligned}$$

for some other constant *F*.^33^ We therefore have the simple estimate

$$\begin{aligned} ||\alpha ^{(n)}||_l \le C_{n-1} (1+L)^n (1+F)^n ||\alpha ||^n_l < \widetilde{C}^n ||\alpha ||^n_l \end{aligned}$$

with $\widetilde{C} = 4 (1+L)(1+F)$. Therefore, the sum converges in $||\cdot ||_l$ for $|t|< 1/ (\widetilde{C}||\alpha ||_l)^n$.

□

Equivalently, we can set t=1 by letting $||\alpha ||_l$ small enough, i.e. there exists some open set $\widetilde{U}_{A_0} \subset \Omega ^1$ on which the formal power series $\widetilde{\delta }_{A_0}(\alpha )$ converges, i.e. there is a map

55 $$\begin{aligned} \widetilde{\delta }_{A_0}:\Omega ^1_l \supset \widetilde{U}_{A_0} \rightarrow \Omega ^1_l \end{aligned}$$

given by (51) with t=1.

##### Remark 2.10

A priori, the limit of this power series lives in the completion $\Omega ^{1}_l$ of $\Omega ^1$ with respect to $||\cdot ||_l$. However, under the additional Assumption 2.4 of Fréchet continuity the sum actually converges in the Fréchet topology and $\widetilde{\delta }_{A_0}$ actually lands in $\Omega ^1$.

Convergence of the map $\widetilde{\delta }_{A_0}$ implies that there are well-defined maps

56 $$\begin{aligned}&\delta _{A_0} = \widetilde{\delta }_{A_0} \circ i_{A_0} :U_{A_0} \rightarrow \Omega ^1, \end{aligned}$$

57 $$\begin{aligned}&\varphi _{A_0} = A_0 + \delta _{A_0} :U_{A_0} \rightarrow \textrm{FC}^\textrm{sm}, \end{aligned}$$

58 $$\begin{aligned}&\widetilde{\psi }_{A_0} :\widetilde{U}_{A_0} \cap \; \Omega ^1_{d_{A_0}\mathrm {-cl}} \rightarrow \textrm{FC}^\textrm{sm}, \end{aligned}$$

59 $$\begin{aligned}&\widetilde{\varphi }_{A_0} = A_0 + \widetilde{\delta }_{A_0} :\widetilde{U}_{A_0} \rightarrow \Omega ^1. \end{aligned}$$

#### Kuranishi map

The map $\widetilde{\delta }_{A_0}$ admits a compositional inverse known as Kuranishi map.^34^

##### Definition 2.11

We define the Kuranishi map $\widetilde{\kappa }_{A_0}:\Omega ^1 \rightarrow \Omega ^1$ to be given by

60 $$\begin{aligned} \widetilde{\kappa }_{A_0}({\mathfrak {d}}) = {\mathfrak {d}}+ \frac{1}{2}K_{A_0}[{\mathfrak {d}},{\mathfrak {d}}] . \end{aligned}$$

This map is as regular as $K_{A_0}$, in particular if $K_{A_0}$ is *l*-Sobolev bounded (for l≥2) then so is $\widetilde{\kappa }_{A_0}$ and if $K_{A_0}$ is Fréchet continuous then so is $\widetilde{\kappa }_{A_0}$. Some salient properties of the this map are:

##### Proposition 2.12

Assume $r_{A_0}$ satisfies the *l*-Sobolev boundedness assumption for some l≥2 (Assumption 2.3). Then $\widetilde{\kappa }_{A_0}$ extends to the completion $\Omega ^1_l$ and we have:

(i) The map $\widetilde{\kappa }_{A_0} :\Omega ^1_l \rightarrow \Omega ^1_l$ is invertible in a neighborhood $V_{A_0}$ of $0\in \Omega ^1_l$, and
  $$\begin{aligned} V_{A_0} \supset \{{\mathfrak {d}}\in \Omega ^1|\; ||K_{A_0}\textrm{ad}_{\mathfrak {d}}||_\textrm{op} < 1\}. \end{aligned}$$
  Here $||\cdot ||_\textrm{op}$ denotes the operator norm induced by the Sobolev norm $||\cdot ||_l$.
(ii) Recall that there exists an open set $U_{A_0}$ on which $\widetilde{\delta }_{A_0}$ converges. We have $U_{A_0} \subset V_{A_0}$, and on $U_{A_0}$ the inverse of $\widetilde{\kappa }_{A_0}$ is given by the map $\widetilde{\delta }_{A_0}$.
(iii) Define the Maurer-Cartan set $MC_{A_0} \subset \Omega ^1_{A_0}$ by
  61 $$\begin{aligned} MC_{A_0} = \{m \in \Omega ^1_{A_0} \;|\; d_{A_0}m + \frac{1}{2}[m,m] = 0\}. \end{aligned}$$
  Then $$\widetilde{\kappa }_{A_0}(MC_{A_0}) \subset \Omega ^1_{A_0-\textrm{cl}}$$, i.e. $$m \in MC_{A_0}$$ implies $$d_{A_0}\widetilde{\kappa }_{A_0} m = 0$$.
(iv) We have
  62 $$\begin{aligned} \widetilde{\varphi }_{A_0}^{-1}(A_1) = \widetilde{\kappa }_{A_0}(A_1-A_0). \end{aligned}$$

The proof is in Appendix D.1.1.

#### Banach and Fréchet smoothness

We know that $\widetilde{\psi }_{A_0}(\alpha )$ is flat for any closed 1-form α in its domain. We claim that locally, it is actually invertible and thus provides $\textrm{FC}$ with the structure of a Banach manifold around $A_0$.

##### Proposition 2.13

(i) There exists a neighborhood $U_{A_0}$ of 0 in $\Omega ^1_{A_0-\textrm{cl},l}$ such that the map $\widetilde{\psi }_{A_0}:U_{A_0} \rightarrow \textrm{FC}_l\subset \Omega ^1_l$ is a homeomorphism onto its image.
(ii) If $A_1 \in \widetilde{\psi }_{A_0}(U_{A_0})$, then the composition $\widetilde{\psi }_{A_1}^{-1} \circ \widetilde{\psi }_{A_0}$ is smooth^35^ on its domain.

In particular, $\textrm{FC}^\textrm{sm}_l$ is a Banach manifold and $\{\widetilde{\psi }_{A_0}\}_{A_0 \in \textrm{FC}^\textrm{sm}_l}$ is an atlas for $\textrm{FC}^\textrm{sm}_l$. We give the proof in Appendix D.1.2. We then have the following corollary.

##### Proposition 2.14

There is a neighborhood $0 \in V_{A_0} \subset \Omega ^1_{A_0-\textrm{cl},l}$ such that $\widetilde{\delta }_{A_0}$ applied to a closed 1-form $\alpha \in V_{A_0}$ defines a Maurer-Cartan element in $\Omega ^1_{A_0,l}$:

63 $$\begin{aligned} d_{A_0}\widetilde{\delta }_{A_0} (\alpha ) + \frac{1}{2}[\widetilde{\delta }_{A_0}(\alpha ),\widetilde{\delta }_{A_0} (\alpha )] = 0. \end{aligned}$$

##### Proof

We know that $\textrm{FC}^\textrm{sm}_l$ is a Banach manifold and for any $A_0 \in \textrm{FC}^\textrm{sm}$, the map $A_1 \mapsto \widetilde{\kappa }_{A_0}(A_1 - A_0)$ sends $A_1 \in \textrm{FC}^\textrm{sm}_l$ to $\Omega ^1_{A_0-\textrm{cl},l}$ (point iii of Proposition 2.12). We can now invoke the inverse function theorem to conclude that this map is surjective onto a neighborhood $V_{A_0}$ of zero. We then have for all $\alpha \in V_{A_0}$ that $\alpha = \widetilde{\kappa }_{A_0}(m)$, with $m \in MC_{A_0}$. Therefore, $\widetilde{\delta }_{A_0}(\alpha ) = \widetilde{\delta }_{A_0}\widetilde{\kappa }_{A_0}(m) = m$ satisfies the Maurer-Cartan equation.

□

This implies the following corollary.

##### Corollary 2.15

For each point $A_0 \in \textrm{FC}^{\textrm{sm}}_l$, the map

$\widetilde{\varphi }_{A_0}:\Omega ^1_{A_0-\textrm{cl}} \supset U_{A_0} \rightarrow \textrm{FC}_l^\textrm{sm}$ is a chart with inverse given by Equation (62).

In particular, Theorem 2.5 follows. Finally, if $K_{A_0}$ is Fréchet continuous, we also have smoothness of $\textrm{FC}^\textrm{sm}$ in the Fréchet topology:

##### Corollary 2.16

The set of smooth points $\textrm{FC}^{\textrm{sm}}$ is a Fréchet manifold. If $K_{A_0}$ satisfies Assumption 2.4, then for each point $A_0 \in \textrm{FC}^{\textrm{sm}}$, restriction of $\widetilde{\varphi }_{A_0}$ to $\textrm{FC}^\textrm{sm}$ defines a chart $\widetilde{\varphi }_{A_0}:\Omega ^1_{A_0-\textrm{cl}} \supset U_{A_0} \rightarrow \textrm{FC}^\textrm{sm}$.

##### Proof

The maps $\widetilde{\kappa }_{A_0}$ and $\widetilde{\delta }_{A_0}$ are both Fréchet continuous and inverse to each other, hence they are inverse homeomorhisms in the Fréchet topology when restricted to $\textrm{FC}^\textrm{sm}$ and $\Omega ^1_{A_0-\textrm{cl}}$ respectively. Also, note that $\widetilde{\kappa }_{A_0}$ and $\widetilde{\delta }_{A_1}$ are both smooth when considered on open neighborhoods of zero in $\Omega ^1$. Therefore, if $A_1 \in \widetilde{\varphi }_{A_0}(U_{A_0})$, then the composition $\widetilde{\varphi }^{-1}_{A_1} \circ \widetilde{\varphi }_{A_0}$ is smooth as a map between neighborhoods of zero in $\Omega ^1$.^36^ Therefore, its restriction to the subspace $\Omega ^1_{A_0-\textrm{cl}}$ is also smooth. □

This finishes the proof of Theorem 2.6.

##### Remark 2.17

Given a (small) closed form $\alpha \in \Omega ^1_{A_0-\textrm{cl}}$, we now have two different ways to lift it to a flat connection, namely as $\widetilde{\psi }_{A_0}(\alpha )$, or $\widetilde{\varphi }_{A_0}(\alpha )$. By definition, their restrictions to $\operatorname {Im} i_{A_0}$ agree, and they agree up to first order. However, from second order, they disagree. Considering for example $\alpha = d_{A_0}\beta$, we have

$$\begin{aligned} \widetilde{\psi }_{A_0}(d_{A_0}\beta ) = A_0 + d_{A_0}\beta -\frac{1}{2}[\beta ,d_{A_0}\beta ] + \frac{1}{2}[\beta ,[\beta ,d_{A_0}\beta ]] + O(\beta ^4) \end{aligned}$$

whereas the sum-over-trees map is

$$\begin{aligned} &  \widetilde{\varphi }_{A_0}(d_{A_0}\beta ) = A_0 + d_{A_0}\beta - \frac{1}{2}K_{A_0}[d_{A_0}\beta ,d_{A_0}\beta ] + \frac{1}{2}K_{A_0}[d_{A_0}\beta , K_{A_0}[d_{A_0}\beta ,d_{A_0}\beta ]] \\ &  \quad = A_0 + d_{A_0}\beta - \frac{1}{2}[\beta ,d_{A_0}\beta ] + \frac{1}{2} d_{A_0}K_{A_0}[\beta ,d_{A_0}\beta ] +\frac{1}{2} i_{A_0}p_{A_0}[\beta ,d_{A_0}\beta ] + O(\beta ^3). \end{aligned}$$

### Irreducible points

#### Definition 2.18

We say that a flat connection is *irreducible* if $H^0_{A_0}(M,{\mathfrak {g}}) = 0$.

The following example shows that connections can define smooth points without being irreducible.

#### Example 2.19

Let G=SU(2). For p>2 prime, on a lens space *L*(*p*, *q*) with fundamental group ${\mathbb {Z}}_p$, there are, up to conjugation $\frac{p+1}{2}$ different representations labeled by $k = 0,1,\ldots , \frac{p-1}{2}$ defined by

$$\begin{aligned} \rho _k(\gamma ) = \begin{pmatrix} e^{2\pi i k/p} & 0 \\ 0 & -e^{2\pi i k/p} \end{pmatrix}, \end{aligned}$$

with γ the generator of the fundamental group. Clearly those representations are reducible, but for k≠0 all the induced $L_\infty$ operations vanish as $H^1 = 0$ and $H^0 = {\mathfrak {t}}$ is the abelian subalgebra of diagonal matrices.

We denote the irreducible locus by $\textrm{FC}^\textrm{irr}$. On the irreducible locus, the quotient of the gauge group by its center acts freely and properly. Therefore, the quotient of the smooth irreducible locus

64 $$\begin{aligned} \textrm{FC}' = \textrm{FC}^\textrm{sm} \cap \textrm{FC}^\textrm{irr} \end{aligned}$$

by the gauge group is a smooth manifold that we denote by ${\mathcal {M}}' \subset {\mathcal {M}}$.

### Transporting “harmonic” forms

For a given smooth flat connection $A_0$ and SDR data $(i_{A_0},p_{A_0},K_{A_0})$ we will call forms in $\operatorname {Im}(i_{A_0}) = \ker d_{A_0} \cap \ker K_{A_0}$ “harmonic.”^37^

#### Proposition 2.20

Suppose we are given a smooth flat connection $A_0$ and a harmonic 1-form ${\textsf{a}}$. Let

65 $$\begin{aligned} A_t = A_0 + \widetilde{\delta }_{A_0}(t{\textsf{a}}) = \widetilde{\varphi }_{A_0}(t{\textsf{a}}) \end{aligned}$$

be the path of flat connections given by Proposition 2.7. Let χ be another harmonic form. Then, there exists a deformation

66 $$\begin{aligned} \chi _t = \sum _{k\ge 0} t^k \chi ^{(k)} \end{aligned}$$

with $\chi ^{(0)}= \chi$, such that $d_{A_t}\chi _t = K_{A_0}\chi _t = 0$.

#### Proof

We have

$$\begin{aligned} d_{A_t} \chi _t = \left( d_{A_0} + \sum _{k\ge 1}t^k\textrm{ad}_{\alpha ^{(k)}}\right) \left( \sum _{l\ge 0}t^l\chi ^{(l)}\right) = 0 \end{aligned}$$

and again we can look at the equations in powers of *t*:

67 $$\begin{aligned} d_{A_0}\chi ^{(n)} = -\sum _{k+k' = n, k\ge 1} \textrm{ad}_{\alpha ^{(k)}}\chi ^{(k')}. \end{aligned}$$

Similarly to the proof of Proposition 2.7, the right hand side here is a representative of the induced $L_\infty$-operation $l_n'({\textsf{a}},\ldots ,{\textsf{a}},\chi )$, its vanishing in cohomology implies that it is exact and that we can set

68 $$\begin{aligned} \chi ^{(n)} = -K_{A_0}\sum _{k+k' = n, k\ge 1} \textrm{ad}_{\alpha ^{(k)}}\chi ^{(k')}. \end{aligned}$$

□

#### Remark 2.21

Again, one has a similar sum-over-trees formula for $\chi ^{(n)}$, namely it is a sum over rooted binary trees with *n* leaves where one leaf is labeled with χ. I.e. we have that

69 $$\begin{aligned} \chi _t = (d\widetilde{\varphi }_{A_0})_{t{\textsf{a}}}(\chi ). \end{aligned}$$

#### Remark 2.22

One can also understand the deformation (66) of a harmonic form χ as $\chi _t=i_t ([\chi ])$ with $i_t=\sum _{n\ge 0}(-K_{A_0}\textrm{ad}_{\widetilde{\delta }_{A_0}(t{\textsf{a}})})^n\circ i_{A_0}$ the deformation of inclusion $i_{A_0}:H_{A_0}^\bullet \hookrightarrow \Omega ^\bullet$ of cohomology as harmonic forms, induced via homological perturbation lemma from the deformation of the differential $d_{A_0}\rightarrow d_{A_t}=d_{A_0}+\textrm{ad}_{\widetilde{\delta }_{A_0}(t{\textsf{a}})}$ on $\Omega ^\bullet$. Note that the corresponding induced differential on $H_{A_0}^\bullet$ is $d'_t:=\sum _{n\ge 1}\frac{1}{n!} l'_{n+1}(\underbrace{t{\textsf{a}},\ldots ,t{\textsf{a}}}_n,-)$; it vanishes since $A_0$ is assumed to be a smooth point. Hence, $d_{A_t}i_t [\chi ]= i_t d'_t [\chi ]=0$.

Under the additional assumption that $A_0$ is irreducible, Proposition 2.20 can be generalized to paths $A_t$ not restricted to the form (65):

#### Proposition 2.23

Let $A_0\in \textrm{FC}'$ be a smooth irreducible flat connection and let $A_t=A_0+\alpha _t$ with $\alpha _t$ be a path of flat connections with $\alpha _0=0$. Fix a harmonic form χ. Then the path of forms

70 $$\begin{aligned} \chi _t=\sum _{k\ge 0} (-K_{A_0} \textrm{ad}_{\alpha _t})^k\circ \chi \end{aligned}$$

starting at $\chi _{0}=\chi$ satisfies $d_{A_t}\chi _t=K_{A_0}\chi _t=0$.

#### Proof

Property $K_{A_0}\chi _t=0$ is immediate by construction (70). The nontrivial part is proving $d_{A_t}\chi _t=0$.

Due to irreducibility of $A_0$, we only have harmonic 1-forms and 2-forms. We consider these two cases for χ separately.

Let χ be a harmonic 1-form. Then $\chi _t = (d\widetilde{\varphi }_{A_0})_{{\textsf{a}}_t}(\chi )$, where ${\textsf{a}}_t$ is a path of closed forms such that $\widetilde{\varphi }_{A_0}({\textsf{a}}_t)=\alpha _t$, cf. Theorem 2.5. Since $\widetilde{\varphi }_{A_0}(\alpha )$ maps closed 1-forms to flat connections, its derivative maps closed 1-forms to first order deformations of flat connections, i.e., $d_{\widetilde{\varphi }_{A_0}(\alpha )}$-closed forms. Therefore, $d_{A_t}\chi _t=0$.

Now, let χ be a harmonic 2-form. We have

$$\begin{aligned} d_{A_0}\chi _t= &  \underbrace{d_{A_0}\chi }_0-\sum _{k\ge 1} d_{A_0}K_{A_0}\textrm{ad}_{\alpha _t}(-K_{A_0}\textrm{ad}_{\alpha _t})^{k-1}\circ \chi \\= &  - \sum _{k\ge 1}\textrm{ad}_{\alpha _t}(-K_{A_0}\textrm{ad}_{\alpha _t})^{k-1}\circ \chi =- \textrm{ad}_{\alpha _t} \chi _t, \end{aligned}$$

which implies $d_{A_t}\chi _t=0$. Here in transition to the second line, we note that the operator $d_{A_0}K_{A_0}=\textrm{id}-i_{A_0}p_{A_0}-K_{A_0}d_{A_0}$ acts on a 3-form β, and thus this operator acts as identity ($p_{A_0}\beta =0$ by irreducibility of $A_0$ and $d_{A_0}\beta =0$ by degree reason). □

### Exponential maps

The upshot of the previous discussion is the following. Suppose that we are given a smooth family (*i*, *p*, *K*) of SDR data on the smooth irreducible locus $\textrm{FC}' \subset \textrm{FC}$. Then, we have two exponential maps $\widetilde{\varphi }$ and $\widetilde{\psi }$ on $\textrm{FC}'$, defined on an open neighborhood $\widetilde{U} \subset T\textrm{FC}'$ of the zero section, which agree on $\widetilde{U} \cap \operatorname {im}i$. We denote by ${\mathbb {H}}$ the cohomology bundle over $\textrm{FC}'$ - the graded vector bundle with fiber over $A_0$ given by $H^\bullet _{A_0}$, and by $U \subset {\mathbb {H}}$ the preimage of $\widetilde{U}$ under *i*. Then, by restriction of $\widetilde{\varphi }$, we have the map $\varphi = \widetilde{\varphi } \circ i :U \rightarrow \textrm{FC}'$.

#### Lemma 2.24

Suppose the family (*i*, *p*, *K*) is equivariant with respect to the action of the gauge group, i.e. for all ${\textsf{a}}\in H^\bullet _{A_0}$ and $\alpha \in \Omega ^\bullet$ we have

71 $$\begin{aligned} i_{{}^\textrm{g}A_0}({}^\textrm{g}a) = {}^\textrm{g}(i_{A_0}a), \quad p_{{}^\textrm{g}A_0}({}^\textrm{g}\alpha ) = {}^\textrm{g}(p_{A_0}\alpha ), \quad K_{{}^\textrm{g}A_0}({}^\textrm{g}\alpha ) = {}^\textrm{g}(K_{A_0}\alpha ). \end{aligned}$$

Then the map φ is equivariant with respect to gauge transformations,

72 $$\begin{aligned} \varphi _{{}^\textrm{g}A_0}({}^\textrm{g}\alpha ) = {}^\textrm{g}(\varphi _{A_0}\alpha ). \end{aligned}$$

#### Proof

The only ingredients of the map φ are the chain homotopy $K_{A_0}$ and the Lie bracket, which are both equivariant with respect to gauge transformations. □

In particular, the map φ descends to the moduli space and defines a generalized exponential map that we denote by $\underline{\varphi }$:

73 $$\begin{aligned} \underline{\varphi } :U \subset T{\mathcal {M}}\rightarrow {\mathcal {M}}, \qquad ([A_0],[\alpha ] \mapsto [\varphi _{A_0}\alpha ]). \end{aligned}$$

#### Grothendieck connection

The exponential maps $\widetilde{\varphi }$ and $\underline{\varphi }$ induce connections on the tangent bundles (viewed as fiber bundle) of $\textrm{FC}'$ and ${\mathcal {M}}'$. These connections are sometimes called the *Grothendieck connections* (see [CF01, CFT02, CMW19, CMW20]). Here we present a slightly different approach. Namely, let $[A],[\widetilde{A}] \in {\mathcal {M}}'$ and $\alpha \in T_A{\mathcal {M}}'$. If [*A*] and $[\widetilde{A}]$ are close enough, there exists $\widetilde{\alpha } \in T_{\widetilde{A}}{\mathcal {M}}'$ such that

74 $$\begin{aligned} \underline{\varphi }_{\widetilde{A}}\widetilde{\alpha } = \underline{\varphi }_A\alpha \qquad \text {or} \qquad \widetilde{\alpha } = \underline{\varphi }^{-1}_{\widetilde{A}}(\underline{\varphi }_A\alpha ). \end{aligned}$$

##### Definition 2.25

The *Grothendieck connection* $\nabla ^G$ is the fiber bundle connection on $U \subset T{\mathcal {M}}'$ whose parallel transport of $\alpha \in U_A$ from *A* to $\widetilde{A}$ is given by

75 $$\begin{aligned} \widetilde{\alpha } = \underline{\varphi }^{-1}_{\widetilde{A}}(\underline{\varphi }_A\alpha ). \end{aligned}$$

In other words, if $A_t$ is a path of flat connections starting at *A*, then the horizontal lift of this path starting at α is given by $\alpha _t = \underline{\varphi }_{A_t}^{-1}\underline{\varphi }_A\alpha$. From the definition, is it obvious that this connection is flat, since its parallel transport between any two (close enough) points $[A],[\widetilde{A}]$ is independent of the choice of a path between them.

##### Remark 2.26

38 The role of $\nabla ^G$ is that it “recognizes” global objects. More explicitly, $\nabla ^G$ induces a connection in the bundle $\widehat{\textrm{Sym}^\bullet }T^*{\mathcal {M}}'$ of formal functions on ${\mathcal {M}}'$—let us also denote it $\nabla ^G$ by abuse of notations. Then a section σ of $\widehat{\textrm{Sym}^\bullet } T^*{\mathcal {M}}'$ (a formal function) is of the form $\textrm{T}\underline{\varphi }^* f$ for some $f\in C^\infty ({\mathcal {M}}')$ (an actual, “global,” function) if and only if σ is horizontal w.r.t. $\nabla ^G$:

76 $$\begin{aligned} \nabla ^G\sigma =0. \end{aligned}$$

Here $\textrm{T}$ stands for the Taylor expansion of a function on *U* in vertical (tangent) coordinates on $T{\mathcal {M}}'$. This discussion applies to any manifold with a formal exponential map, not just $({\mathcal {M}}',\underline{\varphi })$. Also, one can replace functions with half-densities (especially relevant for BV formalism), differential forms, spinors, etc.

### Gauge fixing operators

We now specialize to SDR data defined by gauge-fixing operators.

#### Definition 2.27

*(Gauge-fixing operator)*. We say that ${\textsf{h}}:\Omega ^{\bullet }(M,{\mathfrak {g}}) \rightarrow \Omega ^{\bullet -1}(M,{\mathfrak {g}})$ is a gauge fixing operator for $d_{A_0}$ if the operator^39^

77 $$\begin{aligned} {\mathcal {H}}={\mathcal {H}}_{d_{A_0},{\textsf{h}}}:=[d_{A_0},{\textsf{h}}] :\Omega ^{\bullet }(M,{\mathfrak {g}}) \rightarrow \Omega ^{\bullet }(M,{\mathfrak {g}}) \end{aligned}$$

is a generalized Laplacian, i.e. has symbol $\sigma _2(H)(x,\xi ) = |\xi |^2$.

#### Example 2.28

If *g* is a Riemannian metric on *M*, then the formal adjoint $$d^*_{A_0}$$ of $$d_{A_0}$$ is a gauge fixing operator, with $${\mathcal {H}}_{d_{A_0},d^*_{A_0}} = \Delta _{A_0}$$ the (twisted) Hodge-de Rham Laplacian. In fact, if $A^{\prime }$ is a different flat connection, then $$d^*_{A'}$$ is still a gauge-fixing operator for $$d_{A_0}$$, because the difference

$$\begin{aligned} {\mathcal {H}}_{d_{A_0},d^*_{A_0}} - {\mathcal {H}}_{d_{A_0},d^*_{A'}} = [d_{A_0}, \textrm{ad}_{A_0-A'}^*] \end{aligned}$$

is a first-order differential operator.

#### Good gauge fixing operators

Let ${\textsf{h}}$ be a gauge fixing operator for $d_{A_0}$ and ${\mathcal {H}}= [d_{A_0},{\textsf{h}}]$ the corresponding generalized Laplacian.

##### Definition 2.29

We say that ${\textsf{h}}$ is a good gauge fixing operator if

1. ${\textsf{h}}$ is skew-selfadjoint with respect to Poincaré pairing,
2. $${\textsf{h}}^2 = 0$$,
3. the eigenvalues of ${\mathcal {H}}$ have nonnegative real part,
4. there is a Hodge decomposition
  78 $$\begin{aligned} \Omega = \ker {\mathcal {H}}\oplus \underbrace{\operatorname {im} d_{A_0} \oplus \operatorname {im} h}_{\operatorname {im} {\mathcal {H}}} , \end{aligned}$$
5. we have $$\ker {\mathcal {H}}\cong H^\bullet _{A_0}$$.

Denote *P* the projection onto the kernel of ${\mathcal {H}}$ along the image of ${\mathcal {H}}$. The operator ${\mathcal {H}}+ P$ is invertible and we denote $G:=({\mathcal {H}}+P)^{-1}$ its inverse. It satisfies

79 $$\begin{aligned} {\mathcal {H}}G = G {\mathcal {H}}= \textrm{id} - P. \end{aligned}$$

Also, defining $K = {\textsf{h}}\circ G$ we have $[d_{A_0},K] = \operatorname {id} - P$.

For good gauge-fixing operators, we thus have a strong deformation retraction (SDR)

80 $$\begin{aligned} \begin{aligned} i:H^\bullet _{A_0}\cong \ker {\mathcal {H}}\hookrightarrow \Omega ^\bullet , \\ p:\Omega ^\bullet \twoheadrightarrow \ker {\mathcal {H}}\cong H^\bullet _{A_0}, \\ K= {\textsf{h}}\circ G :\Omega ^\bullet \rightarrow \Omega ^{\bullet - 1}. \end{aligned} \end{aligned}$$

##### Example 2.30

*(Hodge decomposition)*. The main example of a good gauge fixing operator is, given the choice of a Riemannian metric on *M*, the codifferential $d^*_{A_0}$. The fact that $d^*_{A_0}$ is a good gauge-fixing operator follows from the well-known Hodge decomposition.^40^ In this case

- The operator ${\mathcal {H}}= \Delta _{A_0}$ is the Hodge-de Rham Laplacian (twisted by the flat connection $A_0$),
- the map $i_{A_0} :H^\bullet _{A_0} \rightarrow \ker \Delta _{A_0}$ is the isomorphism between de Rham cohomology and harmonic forms, composed with the inclusion into $\Omega ^\bullet$,
- the decomposition (78) is orthogonal,
- and $p_{A_0} = i_{A_0}^{-1} P_{A_0}$ is the orthogonal projection to harmonic forms, composed with the isomorphism with de Rham cohomology.

Moreover, the family of SDR data defined by $A_0 \mapsto (i_{A_0},P_{A_0},K_{A_0})$ defines a global, equivariant family of SDR data and in particular induces a formal exponential map on ${\mathcal {M}}'$ as explained in Sect. 2.4.

##### Proposition 2.31

On a compact manifold *M*, for any $A_0$ and $k \in {\mathbb {N}}, p \ge 2$, the SDR data $(i_{A_0},p_{A_0},K_{A_0})$ is bounded with respect to the Sobolev norm $||\cdot ||_{k,p}$. In particular, the SDR data is Fréchet continuous (satisfies Assumption 2.4.

##### Proof

The maps $i_{A_0}$ and $p_{A_0}$ are bounded since they have finite-dimensional domain (resp. codomain). Denoting the completion of $\Omega ^\bullet$ with respect to $||\cdot ||_{k,p}$ by $\Omega ^\bullet _{k,p}$, the map *K* is bounded since it is a composition of the bounded maps $G_{A_0} :\Omega ^\bullet _{k,p} \rightarrow \Omega ^\bullet _{k+2,p}$ and $d^*_{A_0}:\Omega ^\bullet _{k+2,p} \rightarrow \Omega ^{\bullet -1}_{k+1,p}$. Therefore it defines a continuous map $K_{A_0}:\Omega ^\bullet _{k,p} \rightarrow \Omega ^{\bullet -1}_{k+1,p}$, and the injection $\Omega ^{\bullet -1}_{k+1,p} \rightarrow \Omega ^{\bullet -1}_{k,p}$ is continuous, which proves the first claim.^41^ Since it is bounded with respect to all Sobolev norms, it is also continuous in the Fréchet topology. □

##### Lemma 2.32

(Variation of ${\textsf{h}}$). An infinitesimal variation of a good gauge-fixing operator ${\textsf{h}}\rightarrow {\textsf{h}}+\delta {\textsf{h}}$ induces the following first-order deformation of the SDR (80): $i\rightarrow i+\delta i$, $p\rightarrow p+\delta p$, $K\rightarrow K+\delta K$ with

81 $$\begin{aligned} \delta i=-d_{A_0} {\mathbb {I}}_{\delta {\textsf{h}}} i,\quad \delta p =-p {\mathbb {P}}_{\delta {\textsf{h}}} d_{A_0},\quad \delta K= [d_{A_0},\Lambda _{\delta {\textsf{h}}}]+P {\mathbb {P}}_{\delta {\textsf{h}}}+{\mathbb {I}}_{\delta {\textsf{h}}}i. \end{aligned}$$

Here we denoted

82 $$\begin{aligned} {\mathbb {I}}_{\delta {\textsf{h}}}=G\delta {\textsf{h}},\quad {\mathbb {P}}_{\delta {\textsf{h}}}=\delta {\textsf{h}}G,\quad \Lambda _{\delta {\textsf{h}}}=K\delta {\textsf{h}}G. \end{aligned}$$

The proof is similar to the proof of Proposition B.2.

#### Desynchronized Hodge decomposition

##### Proposition 2.33

Let $$A_0\in \textrm{FC}'$$ be a smooth irreducible flat connection. Then there is a neighborhood *U* of $$A_0$$ in $$\textrm{FC}'$$ such that, for any $A^{\prime }\in U$, $$d^*_{A'}$$ is a good gauge fixing operator for $$d_{A_0}$$.

Before giving the proof we need to make the following remark.

##### Remark 2.34

Let $A_0$ be a smooth irreducible flat connection and $A'_t = A_0 + \alpha _t$, with $\alpha _t=\sum _{k \ge 1}t^k \alpha ^{(k)}$, a path of flat connections starting at $A_0$. Dually to Proposition 2.23, one can deform a harmonic form χ satisfying $d_{A_0}\chi = d^*_{A_0}\chi = 0$ to a path $\chi _t=\sum _{k\ge 0}t^k\chi ^{(k)}$ with $\chi ^{(0)}=\chi$, satisfying $d_{A_0}\chi _t = d_{A'_t}^*\chi _t = 0$, with

$$ \chi ^{(n)} = -d_{A_0}G_{A_0}\sum _{k+k' = n, k\ge 1}\textrm{ad}^*_{\alpha ^{(k)}}\chi ^{(k')}. $$

Equivalently,

$$ \chi _t=\sum _{k\ge 0}(-d_{A_0}G_{A_0}\textrm{ad}^*_{\alpha _t})^k\circ \chi . $$

This statement follows by applying Hodge star to (70).

##### Proof of Proposition 2.33

Property (1) follows from integration by parts and property (2) from $(d_{A'}^*)^2 = 0$.

Remark 2.34 implies that, for $A^{\prime }$ an open neighborhood *U* of $$A_0$$, the graded vector space

83 $$\begin{aligned} W:=\ker d_{A_0}\cap \ker d^*_{A'} \end{aligned}$$

satisfies the following:

- *W* coincides with $$\ker {\mathcal {H}}$$ and has constant (graded) rank as $A^{\prime }\in U$ moves away from $$A_0$$. Indeed, one has:
  (i) $$W\subset \ker {\mathcal {H}}$$.
  (ii) At $A'=A_0$, $W=\ker {\mathcal {H}}$ (by usual Hodge decomposition).
  (iii) Rank of *W* is non-decreasing when moving $A^{\prime }$ away from $$A_0$$. Indeed, by Remark 2.34, one can deform a basis of $$A_0$$-harmonic forms to a linear independent set in *W*. (Since linear independence is an open condition.)
  (iv) Rank of $$\ker {\mathcal {H}}$$ is non-increasing as $A^{\prime }$ is moved away from $$A_0$$.—The rank of the kernel in a family of elliptic operator jumps (increases) on a closed subset in the space of parameters.
  These properties imply the claim above: $W=\ker {\mathcal {H}}$ and the rank is constant.
- *W* contains a single representative of each $$d_{A_0}$$-cohomology class. Indeed, the map $$q:W \rightarrow H_{A_0}$$ sending $$\alpha \mapsto [\alpha ]$$ is surjective, since Remark 2.34 constructs a preimage under *q* of a cohomology class [α] as a deformation of its harmonic representative χ along a path $$A'_t$$ from $$A_0$$ to $A^{\prime }$, to a form $$\widetilde{\chi }$$ satisfying $$d_{A_0}\widetilde{\chi }=d^*_{A'}\widetilde{\chi }=0$$. On the other hand, by (a) *W* has constant rank when $A^{\prime }$ is changing, equal to the rank of $$H_{A_0}$$ at $$A'=A_0$$. Hence, the fact that *q* is a surjection implies that it is in fact an isomorphism.

Then, (a) together with (b) proves (5).

For (4), note that $${\mathcal {H}}$$ is diagonalizable at $$A'=A_0$$ and hence is diagonalizable for $A^{\prime }$ in a neighborhood of $$A_0$$ (since diagonalizability is an open condition). Thus, $$\Omega = \ker {\mathcal {H}}\oplus \textrm{im}{\mathcal {H}}$$ for $A^{\prime }\in U$,—splitting into zero-modes of $${\mathcal {H}}$$ and $$\textrm{im}{\mathcal {H}}=:V$$—the span of eigenforms of $${\mathcal {H}}$$ with nonzero eigenvalues. Operators $$d_{A_0}$$ and $$d^*_{A'}$$ act on *V* (since they commute with $${\mathcal {H}}$$). Moreover, one has

84 $$\begin{aligned} \textrm{im}{\mathcal {H}}=\textrm{im}(d_{A_0})\oplus \textrm{im}(d^*_{A'}) \end{aligned}$$

Indeed, the intersection of the summands on the right is zero: if $d_{A_0}\alpha =d^*_{A'}\alpha =0$, then $\alpha \in \ker d_{A_0}\cap \ker d^*_{A'}=\ker {\mathcal {H}}$, but since $\alpha \in V$ it must be zero. Also, if $\alpha \in V$ then $\alpha =d_{A_0} \beta + d^*_{A'}\gamma$ with $\beta =d^*_{A'}{\mathcal {H}}^{-1}\alpha$ and $\gamma =d_{A_0}{\mathcal {H}}^{-1}\alpha$ (here we are using that ${\mathcal {H}}$ is invertible on *V*). This proves that (84) is a direct sum.

Property (3) is obvious by a continuity argument: in the deformation of $A^{\prime }$ away from $$A_0$$ (in a small enough neighborhood), zero modes of $${\mathcal {H}}$$ are deformed to zero modes while eigenvectors with eigenvalues λ>0 are deformed to eigenvectors with $$\textrm{Re}(\lambda )>0$$. □

##### Definition 2.35

1. If $$A_0$$ and *U* are as in Proposition 2.33 then for any $A^{\prime }\in U$ we say that $$(A_0, A')$$ is a pair of *close* flat connections.
2. If $(A_0,A')$ is a pair of close flat connections, we call the space (83) the *space of* $(A_0,A')$*-harmonic forms* and denote it $\textrm{Harm}_{A_0,A'}$. We also call the associated decomposition (78) the *desynchronized Hodge decomposition*:
  85 $$\begin{aligned} \Omega (M,{\mathfrak {g}})=\textrm{Harm}_{A_0,A'}\oplus \textrm{im}\, d_{A_0} \oplus \textrm{im}\, d^*_{A'}. \end{aligned}$$

Further in this section we will suppress the subscript in $A_0$ and just denote it *A*.

##### Remark 2.36

Notice that Proposition 2.31 is true also for the desynchronized Hodge gauge fixing, since we are only perturbing $\Delta _{A_0}$ by differential operators of degree one or less, so its inverse is still a degree -2 operator.

#### Connection on the bundle of $\left(A,A^{\prime }\right)$-harmonic forms

Let $$\mathcal {U}\subset \textrm{FC}'\times \textrm{FC}'$$ be an open neighborhood of the diagonal in $$\textrm{FC}'\times \textrm{FC}'$$ obtained as the union of open sets *U* from Proposition 2.33. Consider the vector bundle $$\textrm{Harm}$$ over $$\mathcal {U}$$ whose fiber over $\left(A,A^{\prime }\right)$ is the space of $\left(A,A^{\prime }\right)$-harmonic forms $$\textrm{Harm}_{A,A'}$$.

Consider the connection $\nabla ^\textrm{Harm}$ on the bundle $\textrm{Harm}$ defined by infinitesimal parallel transport as follows. If $\chi \in \textrm{Harm}_{A,A'}$ is a harmonic form, then:

(i) For any $$\alpha \in \Omega ^1_{d_{A}\mathrm {-closed}}$$, when moving from $\left(A,A^{\prime }\right)$ to $\left(A+t\alpha ,A^{\prime }\right)$ on $$\mathcal {U}$$, χ transforms to
  86 $$\begin{aligned} \chi -t d^*_{A'}G_{A,A'}\textrm{ad}_\alpha \chi \quad \in \textrm{Harm}_{A+t\alpha ,A'}. \end{aligned}$$
(ii) For any $$\beta \in \Omega ^1_{d_{A'}\mathrm {-closed}}$$, when moving from $\left(A,A^{\prime }\right)$ to $\left(A,A^{\prime }+s\beta \right)$ on $$\mathcal {U}$$, χ transforms to
  87 $$\begin{aligned} \chi -s d_{A}G_{A,A'}\textrm{ad}^*_\beta \chi \quad \in \textrm{Harm}_{A,A'+s\beta }. \end{aligned}$$

The formulae above are written in the first order in deformation parameters *s*, *t*. One can consider $\nabla ^\textrm{Harm}$ as a connection in the trivial bundle over $\mathcal {U}$ with fiber $\Omega ^\bullet (M,{\mathfrak {g}})$ preserving the subbundle $\textrm{Harm}$. The covariant derivative operator associated with the connection $\nabla ^\textrm{Harm}$ is

88 $$\begin{aligned} \nabla ^\textrm{Harm}=\delta -G_{A,A'}\Big (d^*_{A'}\textrm{ad}_{\delta A}+d_{A}\textrm{ad}^*_{\delta A'}\Big ) \end{aligned}$$

with δ the de Rham operator on $\textrm{FC}'\times \textrm{FC}'$.

##### Remark 2.37

One can think of $$\nabla ^\textrm{Harm}$$ as a “shift-and-project” connection: its infinitesimal parallel transport takes an $\left(A,A^{\prime }\right)$-harmonic form χ over $\left(A,A^{\prime }\right)$ and moves it to the $$(A+t\alpha ,A'+s\beta )$$-harmonic form $$P_{A+t\alpha ,A'+s\beta }(\chi )$$ over $$(A+t\alpha ,A'+s\beta )$$. We note that this construction is reminiscent of the construction of Hitchin’s (projectively flat) connection in the Verlinde bundle^42^ over the moduli space of complex structures on a surface Σ.

One has the following:

##### Proposition 2.38

**Fig. 4:**
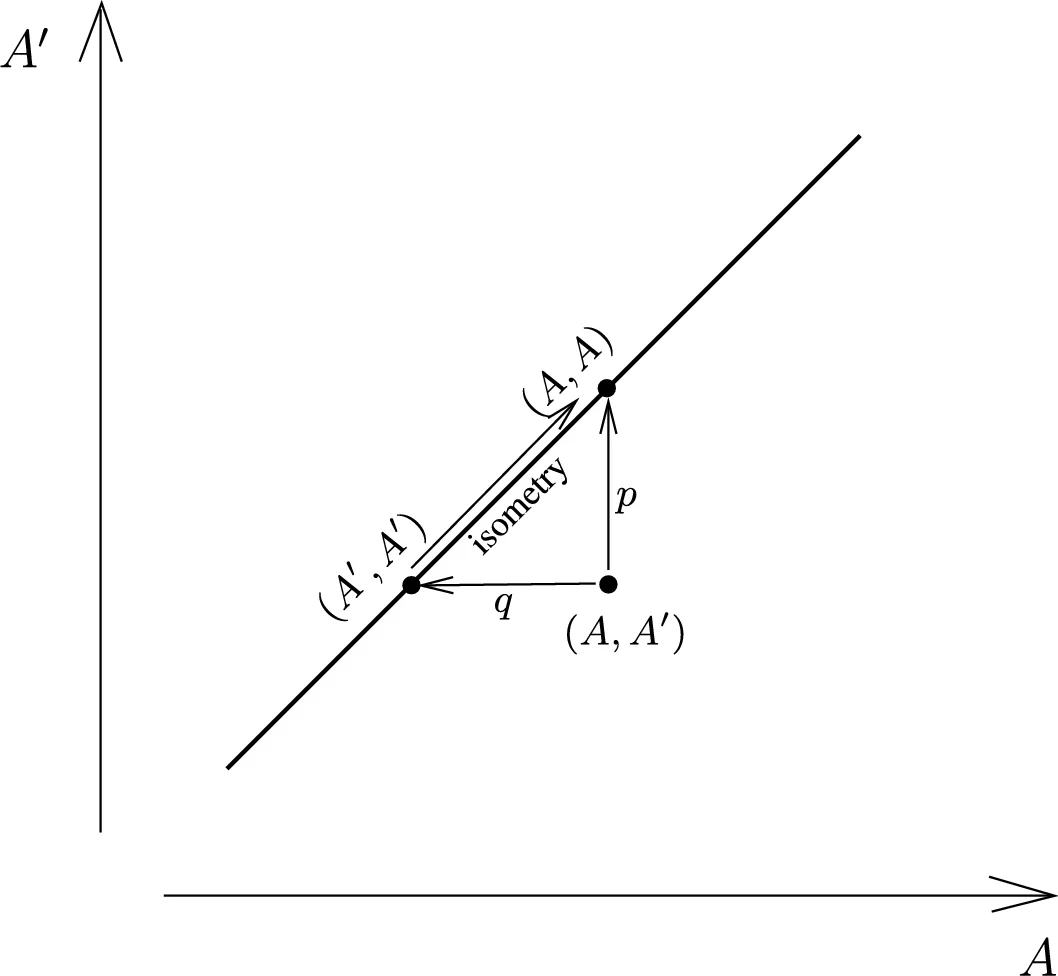
Vertical and horizontal parallel transport to the diagonal on $\textrm{FC}'\times \textrm{FC}'$ (Proposition 2.38 (c))

- The curvature of the connection $\nabla ^\textrm{Harm}$ (restricted to harmonic forms) is
  89 $$\begin{aligned}&F_{\nabla ^\textrm{Harm}}=-P\textrm{ad}_{\delta A} G \textrm{ad}^*_{\delta A'} P- P\textrm{ad}^*_{\delta A'} G \textrm{ad}_{\delta A} P \nonumber \\&\quad \in \Omega ^{1,1}(\mathcal {U},\textrm{End}(\textrm{Harm}_{A,A'})), \end{aligned}$$
  where we suppress subscripts in $$P_{A,A'},G_{A,A'}$$. In particular, $$\nabla ^\textrm{Harm}$$ is flat on $A^{\prime }$-fixed and on *A*-fixed slices of $$\mathcal {U}$$.
- The restriction of $\nabla ^\textrm{Harm}$ to the diagonal in $\mathcal {U}\subset \textrm{FC}'\times \textrm{FC}'$ is a Euclidean connection—it preserves the Hodge inner product on harmonic forms.
- Given a path $$A_t$$ of flat connections $$0\le t\le 1$$, from *A* to $A^{\prime }$, the parallel transport of an $\left(A,A^{\prime }\right)$-harmonic form χ along the path $$(A_t,A')$$ is $$q(\chi )\in \textrm{Harm}_{A',A'}$$ with $$q:\textrm{Harm}_{A,A'}\xrightarrow {\sim } \textrm{Harm}_{A',A'}$$ the orthogonal projection onto $A^{\prime }$-harmonic forms. Likewise, the parallel transport of χ along the path $$(A,A_{1-t})$$ is $$p(\chi )\in \textrm{Harm}_{A,A}$$ with $$p:\textrm{Harm}_{A,A'}\xrightarrow {\sim } \textrm{Harm}_{A,A}$$ the orthogonal projection onto *A*-harmonic forms (Fig. 4).

##### Proof

(a): Note that the connection (88) can be equivalently written as

$$ \nabla ^\textrm{Harm}=\delta -G([d^*,\textrm{ad}_{\delta A}]+[d,\textrm{ad}^*_{\delta A'}]) = \delta + G \delta \Delta . $$

Therefore, the curvature (on harmonic forms) is

90 $$\begin{aligned} (\nabla ^\textrm{Harm})^2 P&= (\delta G \delta \Delta + G \,\delta \Delta \, G \,\delta \Delta )P \nonumber \\&=\Big (G\Big (\underbrace{[d^*,\textrm{ad}_{\delta A}]+[d,\textrm{ad}^*_{\delta A'}]}_{-\delta \Delta }-K\textrm{ad}_{\delta A}P+P\textrm{ad}_{\delta A}K \nonumber \\&\quad -dG \textrm{ad}^*_{\delta A'} P+P\textrm{ad}^*_{\delta A'} Gd\Big ) G\delta \Delta + G\delta \Delta G \delta \Delta \Big )P\nonumber \\&=-P\textrm{ad}_{\delta A}\underbrace{KGd}_{G P_\textrm{coex}} \textrm{ad}^*_{\delta A'} P- P\textrm{ad}^*_{\delta A'} G\underbrace{d G d^*}_{P_\textrm{ex}} \textrm{ad}_{\delta A} P, \end{aligned}$$

which simplifies to (89).

(b): Infinitesimal parallel transport along the diagonal in $$\textrm{FC}'\times \textrm{FC}'$$, from (*A*, *A*) to $$(A+t\alpha ,A+t\alpha )$$ transform a harmonic form $$\chi \in \textrm{Harm}_{A,A}$$ to $$\chi '=\chi -t d^*_{A}G_{A,A} \textrm{ad}_\alpha \chi -t d_{A} G_{A,A} \textrm{ad}^*_{\alpha }\chi $$. Note that the three summands in $\chi^{\prime }$ are mutually orthogonal and two of them are of order *t*, hence $$||\chi '||=||\chi ||+O(t^2)$$. Therefore, if $$A_t$$ is a path of flat connections and $$\chi _t\in \textrm{Harm}_{A_t,A_t}$$ is the parallel transport of χ along the corresponding path in the diagonal in $$\textrm{FC}'\times \textrm{FC}'$$, then $$\frac{d}{dt}||\chi _t ||=0$$.

(c): The form of the connection (86) implies that the parallel transport from $\left(A^{\prime },A^{\prime }\right)$ to $\left(A,A^{\prime }\right)$ transforms an $A^{\prime }$-harmonic form ψ to $$\psi +d^*_{A'}(\cdots )=\chi $$. Hence, the reverse parallel transport transforms an $$d^*_{A'}$$-closed form χ to its projection q(χ) onto $A^{\prime }$-harmonic forms. The case of moving from $\left(A,A^{\prime }\right)$ to (*A*, *A*) is analogous. □

##### Remark 2.39

The connection $\nabla ^\textrm{Harm}$ is a rephrasing of the result of Proposition 2.20 and Remark 2.34 (and for paths considered in that Proposition and Remark, $\nabla ^\textrm{Harm}$ gives the same parallel transport).

#### Cohomology comparison map

Let $${\mathbb {H}}$$ be the “cohomology bundle” over $$\textrm{FC}'$$—the graded vector bundle with the fiber over *A* being $$H^\bullet _{A}$$. For a fixed $$A'\in \textrm{FC}'$$, let $$\mathcal {U}_{A'}:=\mathcal {U}\cap (\textrm{FC}'\times \{A'\})$$—the $A^{\prime }$-fixed slice of $$\mathcal {U}$$. The connection $$\nabla ^\textrm{Harm}$$ of Sect. 2.5.3 restricted to $$\mathcal {U}_{A'}$$ induces (via the isomorphism $$\textrm{Harm}_{A,A'}\cong H_{A}$$, $$\chi \mapsto [\chi ]$$) a flat connection $$\nabla ^{{\mathbb {H}},A'}$$ in $${\mathbb {H}}|_{\mathcal {U}_{A'}}$$.

For $$A,\widetilde{A}$$ a pair of close flat connections, close to $A^{\prime }$, we will call the parallel transport of $$\nabla ^{{\mathbb {H}},A'}$$—the linear map

91 $$\begin{aligned} {\mathfrak {B}}_{\widetilde{A}\leftarrow A;A'}:H^\bullet _{A}\rightarrow H^\bullet _{\widetilde{A}} \end{aligned}$$

– the “cohomology comparison map.” Notice that due to the curvature of $$\nabla ^\textrm{Harm}$$, this map depends nontrivially on $A^{\prime }$.

Sometimes we will need the cohomology comparison map restricted to cohomology in degree 1; we will denote it ${\mathfrak {B}}^1_{\widetilde{A}\leftarrow A;A'}$.

##### Remark 2.40

In the special case $\widetilde{A}=A'$ the cohomology comparison map is

$$\begin{aligned} {\mathfrak {B}}_{A'\leftarrow A;A'}=[q]:H_{A}\rightarrow H_{A'} \end{aligned}$$

– the map induced in cohomology by the map *q* of Proposition 2.38 (c).

#### Local exponential map for fixed $A^{\prime }$.

For a given smooth flat connection $A^{\prime }$, for $$A \in \mathcal {U}_{A'}$$, we have the desynchronized Hodge SDR data $$(i_{A,A'},p_{A,A'},K_{A,A'})$$. These induce, locally around $A^{\prime }$, a sum-over-trees exponential map that we denote $$\widetilde{\varphi }_{\bullet ,A'}:T\textrm{FC}' \supset V_A \rightarrow \textrm{FC}'$$. Contrary to the “global” exponential map $$\widetilde{\varphi }_{\bullet ,\bullet }$$, the local exponential map $$\widetilde{\varphi }_{\bullet ,A'}$$ is not equivariant with respect to the gauge group action on its argument. However, it satisfies the following “convolution” property:

##### Proposition 2.41

Let $\beta , \gamma \in T_A\textrm{FC}'$ such that $\beta , \gamma$ and $\beta + \gamma$ are in the domain of $\varphi _{A,A'}$. Let $\widetilde{A} = \widetilde{\varphi }_{A,A'}(\beta )$. Then

92 $$\begin{aligned} \widetilde{\varphi }_{A,A'}(\beta + \gamma ) = \widetilde{\varphi }_{\widetilde{A},A'}( (d\widetilde{\varphi }_{A,A'})_\beta (\gamma )). \end{aligned}$$

##### Proof

The proof follows from the combinatorics of tress and the homological perturbation lemma. Namely, one can expand the left hand side as a sum-over-trees map where edges are decorated by $K_{A,A'}$ and leaves are decorated either by β or by γ. On the other hand, one can expand the right hand side as a sum-over-trees map where edges are decorated by $K_{\widetilde{A},A'}$ and leaves are decorated by ${\mathfrak {B}}^1_{\widetilde{A}\leftarrow A;A'}(\gamma )$. By the homological perturbation lemma, we have $K_{\widetilde{A},A'} = K_{A,A'} - K_{A,A'}\delta _{A,A'}K_{A,A'} + \ldots$, which we can represent as a sum over ways to plug in a forest of trees into an edge, with leaves decorated by β and edges decorated by $K_{A,A'}$. Finally, $(d\widetilde{\varphi }_{A,A'})_\beta (\gamma )$ is itself given as a sum over trees with edges labeled by $K_{A,A'}$, one leaf labeled γ and all other leaves labeled β (see Remark 2.21). In this way, one also on the right hand side obtains a sum over trees with edges labeled by $K_{A,A'}$ and leaves either labeled β or γ. The numerical prefactor of each such tree is the same on both sides and given by $(-1) ^{n-1}/|\operatorname {Aut} T|$ where *n* is the number of leaves and $\operatorname {Aut} T$ are the automorphisms of *T* respecting the decorations of leaves. □

This then leads to the following explicit description of the Grothendieck connection on $\textrm{FC}'$: Let $\alpha , \beta \in T_A\textrm{FC}'$, and $\widetilde{A} = \widetilde{\varphi }_{A,A'}(\beta )$. Denote $\widetilde{\alpha }$ the parallel transport of α from $A \rightarrow \widetilde{A}$.

##### Proposition 2.42

We have

93 $$\begin{aligned} \widetilde{\alpha }= (d\widetilde{\varphi }_{A,A'})_\beta (\alpha - \beta ). \end{aligned}$$

##### Proof

This follows from the fact that $\widetilde{\alpha }= \widetilde{\varphi }^{-1}_{\widetilde{A},A'}\widetilde{\varphi }_{A,A'}$ and Proposition 2.41 by choosing γ such that $\alpha = \gamma + \beta$. □

By restricting to harmonic forms and passing to cohomology, we obtain a local exponential map $\varphi _{A,A'}:V_{A'} \rightarrow \mathcal {U}_{A'}$, defined on an open subset $V_{A'}$ of the cohomology bundle ${\mathbb {H}}\big |_{\mathcal {U}_A}\rightarrow \mathcal {U}_{A'}$. Associated to this map is a partial fiber bundle connection, whose parallel transport can be defined as follows: Let $\alpha , \beta \in H^1_A$ and $\widetilde{A} = \varphi _{A,A'}(\alpha )$. Then the parallel transport of α from *A* to $\widetilde{A}$ is given by the cohomology comparison map

94 $$\begin{aligned} \widetilde{\alpha } = {\mathfrak {B}}_{\widetilde{A} \leftarrow A,A'}(\alpha - \beta ) \end{aligned}$$

since by Remark 2.39 the parallel transport of $\nabla ^\textrm{Harm}$ from *A* to $\widetilde{A} = \varphi _{A,A'}(\beta )$ is given by $(d\varphi _{A,A'})_\beta$. Cf. also Definition 4.7 and Remark 4.8.

## Perturbative Chern–Simons Partition Function in the BV Formalism

In this section we recall the definition of the perturbative Chern–Simons partition function at an arbitrary reference flat connection $A_0$ given in [CM08] (a detailed review can be found also in [Mne19, Wer22]), and extend this to the definition of the desynchronized partition function which uses as gauge fixing operator the codifferential $d^*_{A'}$ instead of $d^*_{A_0}$. Let *G* be a simple, compact and simply connected Lie group and $\langle \cdot ,\cdot \rangle$ an $\textrm{ad}$-invariant pairing on ${\mathfrak {g}}$. Let *P* be a principal *G*-bundle on a 3-manifold *M*, we will assume that a trivialization^43^ of *P* has been fixed: $P \cong M \times G$. We can therefore identify connections with ${\mathfrak {g}}$-valued 1-forms. Our convention for the Chern–Simons action $S_{CS} :\Omega ^1(M,{\mathfrak {g}}) \rightarrow {\mathbb {R}}$ is

95 $$\begin{aligned} S_{CS}(A) = \int _M \frac{1}{2} \langle A, dA\rangle + \frac{1}{6}\langle A,[A,A]\rangle . \end{aligned}$$

Its critical points are the flat connections, i.e. those 1-forms $A_0 \in \Omega ^1(M,{\mathfrak {g}})$ satisfying

96 $$\begin{aligned} dA_0 + \frac{1}{2}[A_0,A_0] = 0. \end{aligned}$$

For a flat connection $A_0 \in \Omega ^1(M,{\mathfrak {g}})$, we denote the twisted de Rham differential by

97 $$\begin{aligned} d_{A_0} = d + [A_0,\cdot ] :\Omega ^\bullet \rightarrow \Omega ^{\bullet + 1} \end{aligned}$$

and the $A_0$-twisted de Rham cohomology by $H_{A_0}^\bullet$.

### Perturbative partition function

We can now proceed with the definition of the perturbative Chern–Simons partition function at $A_0$. Formally, we want to define it as the perturbative evaluation of the BV pushforward

$$\begin{aligned} Z_{\textsf{h}}(A_0,{\textsf{a}}) = \int _{\alpha _\textrm{fl} \in \operatorname {im} {\textsf{h}}}e^{\frac{i}{\hbar }S_{CS}(A_0 + {\textsf{a}}+\alpha _\textrm{fl})}\mu ^{\frac{1}{2}}. \end{aligned}$$

We first define the partition function with gauge fixing operator ${\textsf{h}}=d^*_{A_0}$, and then comment on changing the gauge fixing operator.

#### Definition 3.1

Let $A_0$ be a flat connection on *M*, and *g* a Riemannian metric on *M*. The Chern–Simons partition function at $A_0$ with gauge fixing operator $d^*_{A_0}$ is defined by

98 $$\begin{aligned}&Z(A_0,{\textsf{a}};g):=e^{\frac{i}{\hbar }S_{CS}(A_0)} \tau (A_0)^\frac{1}{2} e^{\frac{\pi i}{4}\psi (A_0;g)}\cdot \nonumber \\&\quad \cdot \exp \left( \sum _\Gamma \frac{(-i\hbar )^{-\chi (\Gamma )}}{|\textrm{Aut}(\Gamma )|}\Phi _{\Gamma ,A_0;g}({\textsf{a}})\right) \nonumber \\&\quad \in e^{\frac{i}{\hbar }\big (S_{CS}(A_0)+\sum _{n\ge 2}\frac{1}{(n+1)!}\langle {\textsf{a}},l_n({\textsf{a}},\ldots ,{\textsf{a}})\rangle \big )}\cdot \textrm{Det}^\frac{1}{2}(H^\bullet _{A_0})\otimes \widehat{\textrm{Sym}}(H^\bullet _{A_0}[1])^*[[\hbar ]] \end{aligned}$$

– a formal half-density on de Rham cohomology twisted by $A_0$.^44^ Here:

- $S_{CS}(A_0)$ is the value of Chern–Simons action (95) on $A_0$.
- $\tau (A_0)\in \textrm{Det}(H^\bullet _{A_0})$ is the Ray-Singer torsion of *M* with local system $A_0$. $\tau (A_0)^{\frac{1}{2}}\in \textrm{Det}^{\frac{1}{2}}(H^\bullet _{A_0})$ is its square root.
- $\psi (A_0;g)$ is the Atiyah-Patodi-Singer eta-invariant of the operator $L_-:=*d_{A_0}+d_{A_0}*$ acting on forms of odd degree.
- The sum ranges over connected 3-valent graphs (“Feynman graphs”) Γ with leaves (loose half-edges) allowed. $\chi (\Gamma )$ is the Euler characteristic of the graph and $\textrm{Aut}(\Gamma )$ is the automorphism group. The weight of a graph Γ is a polynomial in ${\textsf{a}}$ defined as
  99 $$\begin{aligned}&\Phi _{\Gamma ,A_0;g}({\textsf{a}})= \nonumber \\&\quad \int _{\overline{\textrm{Conf}}_V(M)} \left\langle \prod _{\textrm{leaves}\; l} \pi ^*_{v(l)}i_{A_0}({\textsf{a}}) \prod _{\textrm{edges}\, e=(uv)} \pi _{uv}^*\eta _{A_0} \prod _{\mathrm {short\; loops}\; e=(vv)} \pi _v^* \eta _{A_0}^\Delta , \bigotimes _{\textrm{vertices}} f \right\rangle , \end{aligned}$$
  where:
  - $\overline{\textrm{Conf}}_V(M)$ is the Fulton-MacPherson-Axelrod-Singer compactification of the configuration space of $V=\#\{\textrm{vertices}\}$ points on *M*.
  - $\pi _{uv}:\overline{\textrm{Conf}}_V(M) \rightarrow \overline{\textrm{Conf}}_2(M)$ is the map forgetting the positions of all points except points *u* and *v*; similarly, $\pi _v:\overline{\textrm{Conf}}_V(M) \rightarrow M$ is the map forgetting all points except *v*.
  - The propagator $\eta _{A_0}\in \Omega ^2(\overline{\textrm{Conf}}_2(M),{\mathfrak {g}}\otimes {\mathfrak {g}})$ is minus the integral kernel of the operator
    100 $$\begin{aligned} K_{A_0}=d^*_{A_0}(\Delta _{A_0}+P_\textrm{Harm})^{-1} \end{aligned}$$
    —the Hodge chain homotopy between $d_{A_0}$ and projection to harmonic forms.
  - $\eta _{A_0}^\Delta \in \Omega ^2(M,{\mathfrak {g}}\otimes {\mathfrak {g}})$ is the appropriately renormalized evaluation of $\eta _{A_0}$ on the diagonal.^45^
  - $i_{A_0}$ maps a cohomology class to its harmonic representative.
  - $f\in {\mathfrak {g}}^{\otimes 3}$ is the structure tensor of the Lie algebra ${\mathfrak {g}}$.
  - $\langle ,\rangle$ is the inner product on ${\mathfrak {g}}$ extended to ${\mathfrak {g}}^{\otimes \#\{\mathrm {half-edges}\}}$.
  - In (99), the first product is over leaves of Γ, with *v*(*l*) the vertex incident to the leaf; the second product is over edges connecting *distinct* vertices *u*, *v*; the third product is over “short loops”—edges connecting a vertex *v* to itself.

#### Remark 3.2

One can split the partition function according to “loop number” as $Z(A_0,{\textsf{a}})=Z^{(0)}(A_0,{\textsf{a}})Z^{(1)}(A_0,{\textsf{a}})Z^{(\ge 2)}(A_0,{\textsf{a}})$, where

$$\begin{aligned} Z^{(0)}(A_0,{\textsf{a}})&:= \exp \left( \frac{i}{\hbar }\left( S_{CS}(A_0)+\sum _{\Gamma \in \mathrm {Gr_{conn}},\, l(\Gamma ) = 0} \frac{1}{|\operatorname {Aut}(\Gamma )|}\Phi _{\Gamma ,A_0;m}({\textsf{a}}) \right) \right) \\&=\exp \left( \frac{i}{\hbar }\left( S_{CS}(A_0)+\sum _{n \ge 1}\frac{1}{(n+1)!}\langle {\textsf{a}}, l_n({\textsf{a}},\ldots ,{\textsf{a}})\rangle \right) \right) , \\ Z^{(1)}(A_0,{\textsf{a}})&:= \tau (A_0)^{\frac{1}{2}}e^{\frac{\pi i }{4}\psi (A_0;g)} \cdot \exp \left( \sum _{\Gamma \in \mathrm {Gr_{conn}},\, l(\Gamma ) = 1} \frac{1}{|\operatorname {Aut}(\Gamma )|}\Phi _{\Gamma ,A_0;g}({\textsf{a}})\right) \\&\in \textrm{Det}^{\frac{1}{2}}( H^\bullet _{A_0})\otimes \widehat{\operatorname {Sym}}(H^\bullet _{A_0}[1])^*, \\ Z^{(\ge 2)}(A_0,{\textsf{a}})&:= \exp \left( \frac{i}{\hbar }\sum _{\Gamma \in \mathrm {Gr_{conn}},\, l(\Gamma ) \ge 2} \frac{(-i\hbar )^{l(\Gamma )}}{|\operatorname {Aut}(\Gamma )|}\Phi _{\Gamma ,A_0;g}({\textsf{a}})\right) \in \widehat{\operatorname {Sym}}(H^\bullet _{A_0}[1])^*[[\hbar ]]. \end{aligned}$$

Here l(Γ) is the number of loops in a connected graph.

The reason for excluding tree (0-loop) diagrams from the sum in (98) is that they come with a factor of 1/ħ so after taking exponential we would obtain unbounded negative powers oh ħ. Instead, they are included in the prefactor in the form of the induced $L_\infty$ operations $l_n$.

#### Theorem 3.3

The perturbative partition function is closed with respect to the BV Laplacian on zero modes,

101 $$\begin{aligned} \Delta _{\textsf{a}}Z_{A_0}({\textsf{a}}) = 0. \end{aligned}$$

We refer to [Wer22, Section 3.4.2] for the proof. It is in turn an adaptation of the proof of Lemma 4.11 from [CMR17], using Stokes’ theorem for configuration space integrals representing Feynman weights. Also, the case $A_0=0$ is a part of Theorem 1 in [CM08].

#### Remark 3.4

(i) If the flat connection $A_0$ is *irreducible*, then $H^0_{A_0}=H^3_{A_0}=0$. An elementary degree count then shows that $Z(A_0,{\textsf{a}})$ depends only on the 1-form component of ${\textsf{a}}$. In particular, in this case (101) holds trivially.
(ii) If $[A_0]$ is a smooth point in the moduli space of flat connections, then operations $l_n$ on $H^\bullet _{A_0}$ vanish (i.e., the tree graphs in (98) cancel out).

### Desynchronized partition function

Let ${\textsf{h}}$ be a good gauge fixing operator for $A_0$, and $r_{\textsf{h}}= (i_{\textsf{h}},p_{\textsf{h}},K_{\textsf{h}})$ the corresponding SDR data. The goal of this subsection is to define the “desynchronized” perturbative partition function, heuristically given by the BV pushforward

102 $$\begin{aligned} Z_{A_0,{\textsf{h}}}({\textsf{a}}) = \sqrt{\mu '}\int _{\alpha \in \operatorname {im} {\textsf{h}}}e^{\frac{i}{\hbar }S_{CS}(A_0+i_{\textsf{h}}({\textsf{a}})+\alpha )}\sqrt{\mu ''}\bigg |_{\mathcal {L}}\end{aligned}$$

where $$\sqrt{\mu } = \sqrt{\mu '}\sqrt{\mu ''}$$ is the formal Lebesgue half-density on $$\Omega ^\bullet (M,{\mathfrak {g}})[1]$$ and $$\sqrt{\mu '},\sqrt{\mu ''}$$ the Lebesgue half-densities on $$H^\bullet _{A_0}[1] \cong \ker {\mathcal {H}}$$ and $$\operatorname {im}d_{A_0} \oplus \operatorname {im}{\textsf{h}}$$ respectively. For the remainder of this section we fix $${\textsf{h}}= d^*_{A'}$$, for some flat connection $A^{\prime }$ close to $$A_0$$ in the sense of Definition 2.35.

We then define the desynchronized partition function analogously to the synchronized case:

#### Definition 3.5

Let $\left(A,A^{\prime }\right)$ be a pair of close, smooth flat connections. Then we define the *desynchronized partition function*

$$\begin{aligned} Z_{A,A'} \in e^{\frac{i}{\hbar }S_{CS}(A)}\cdot \operatorname {Det}^\frac{1}{2} (H^\bullet _{A})\otimes \widehat{\operatorname {Sym}}(H^\bullet _{A}[1])^*[[\hbar ]] \end{aligned}$$

as the product

103 $$\begin{aligned} Z_{A,A'}({\textsf{a}}) = Z^{(0)}_{A,A'}\,Z^{(1)}_{A,A'}({\textsf{a}})\, Z^{(\ge 2)}_{A,A'}({\textsf{a}}) \end{aligned}$$

where $$Z_{A,A'}^{(0)}:= e^{\frac{i}{\hbar }S_{CS}(A)} $$ and

104 $$\begin{aligned}&Z_{A,A'}^{(1)}({\textsf{a}}) := e^{\frac{\pi i}{4}\psi _{A}} \tau _{A}^{1/2} \exp \left( \sum _{\Gamma \in Gr_{conn}, l(\Gamma ) =1 } \frac{1}{|\operatorname {Aut}(\Gamma )|}\Phi _{\Gamma ,A,A'}({\textsf{a}})\right) \nonumber \\&\quad \in \operatorname {Det}^\frac{1}{2} (H^\bullet _{A})\otimes \widehat{\operatorname {Sym}}(H^\bullet _{A}[1])^*, \end{aligned}$$

105 $$\begin{aligned}&Z_{A,A'}^{(\ge 2)}({\textsf{a}}) := \exp \left( \sum _{\Gamma \in Gr_{conn}, l(\Gamma ) \ge 2} \frac{(-i\hbar )^{l(\Gamma )-1}}{|\operatorname {Aut}(\Gamma )|}\Phi _{\Gamma ,A,A'}({\textsf{a}})\right) \nonumber \\&\quad \in \widehat{\operatorname {Sym}}(H^\bullet _{A}[1])^*[[\hbar ]]. \end{aligned}$$

The Feynman weights $\Phi _{A,A'}(\Gamma )$ are defined as in (99), where we replace the integral kernel (100) of $K_{A}$ by the integral kernel of

106 $$\begin{aligned} K_{A,A'} = d_{A'}^*\circ (\Delta _{A,A'} + P_{A,A'})^{-1} \end{aligned}$$

and the map $i_{A}$ with $i_{A,A'}$.

Notice that since *A* is smooth, there are no trees in the zero-loop part—their weights vanish by the smoothness assumption.

#### Digression: Path integral computation of desynchronized 1-loop part

The abelian part of the path integral (102) is

107 $$\begin{aligned} I_{A,A'} := \sqrt{\mu '} \int _{\alpha \in {\mathcal {L}}= \operatorname {im}d_{A'^*} }e^{\frac{i}{\hbar }\int _M\frac{1}{2}\langle \alpha , d_{A}\alpha \rangle }\sqrt{\mu ''}\bigg |_{\mathcal {L}}. \end{aligned}$$

Perturbative formula (104) corresponds to evaluating the path integral (107) to

108 $$\begin{aligned} I_{A,A'}:= \tau _Ae^{\frac{i\pi }{4}\psi _A}. \end{aligned}$$

In this digression we want to explain why this is a good definition of the r.h.s. of (107). Namely, naive evaluation of this path integral would go along the following lines. For a subspace $V \subset \Omega ^\bullet (M,{\mathfrak {g}})$ and an isomorphism F:V→V we set

$$\begin{aligned} \int _{\alpha \in V} e^{\frac{i}{\hbar }\frac{1}{2}(\alpha , F\alpha )_H}\mu _H = e^{\frac{i\pi }{4}\textrm{sign}F}\operatorname {Sdet}_V^\frac{1}{2}F \end{aligned}$$

where $(\cdot ,\cdot )_H$ denotes the Hodge inner product

$$\begin{aligned} (\alpha _1, \alpha _2)_H = \int _M \langle \alpha _1 {\mathop {,}\limits ^{\wedge }} * \alpha _2 \rangle \end{aligned}$$

and the signature and superdeterminant have to be understood in a regularized sense. Looking at (107), the map $*d_{A}$ maps $d_{A'}$-coexact forms to $d_{A}$-coexact forms, so it is not an endomorphism of ${\mathcal {L}}= \operatorname {im}d_{A'}^*$. The orthogonal (with respect to the Hodge inner product) projector to $\operatorname {im}d_{A'}^*$ is $K_{A'}d_{A'}$, so we obtain

109 $$\begin{aligned} I_{A,A'} = \sqrt{\mu '}\, e^{\frac{i\pi }{4}\operatorname {sign}K_{A'}d_{A'}*d_{A}}\operatorname {Sdet}_{\operatorname {im}d_{A'}^*}^\frac{1}{2}(K_{A'}d_{A'}*d_{A}). \end{aligned}$$

We claim that this coincides with the following definition:

##### Lemma 3.6

For a pair of close flat connections $\left(A,A^{\prime }\right)$, $$I_{A,A'}$$ can be expressed as

110 $$\begin{aligned} I_{A,A'} = e^{\frac{i\pi }{4} \psi _{A'}} \det ({\mathfrak {B}}_{A\leftarrow A';A'})^{1/2} \tau _{A'}^{1/2}\operatorname {Sdet}^{\frac{1}{2}}_{\operatorname {im}d_{A'}^*}\left( 1 + K_{A'}\operatorname {ad}_\beta \right) \in \operatorname {Det}^{\frac{1}{2}} H^\bullet _{A}, \end{aligned}$$

where

- $\psi _{A'}$ is the eta-invariant of $*d_{A'} + d_{A'}*$,
- ${\mathfrak {B}}_{A\leftarrow A';A'}:H^\bullet _{A'}\rightarrow H^\bullet _{A}$ is the cohomology comparison map of Sect. 2.5.4.
- $\operatorname {Sdet}$ denotes a zeta-regularized superdeterminant,
- $\beta =A-A^{\prime }$ is the difference between the two flat connections.

##### Proof

We have on $\operatorname {im}d_{A'}^*$ that $K_{A'}d_{A'} = \textrm{id}$ and therefore

$$\begin{aligned} \textrm{id} + K_{A'}\mathrm {ad_\beta } = \textrm{id} + K_{A'}(d_{A} - d_{A'}) = K_{A'}d_{A}. \end{aligned}$$

Also,

$$ \det ({\mathfrak {B}}_{A\leftarrow A';A'})^{\frac{1}{2}} \tau _{A'}^\frac{1}{2} = \sqrt{\mu '}\operatorname {Sdet}^\frac{1}{2}_{\operatorname {im}d_{A'}^*} *d_{A'}.$$

This implies that

$$\begin{aligned}&\det ({\mathfrak {B}}_{A\leftarrow A';A'})^{\frac{1}{2}} \tau _{A'}^\frac{1}{2}\operatorname {Sdet}^{\frac{1}{2}}_{\operatorname {im}d_{A'}^*}\left( 1 + K_{A'}\operatorname {ad}_\beta \right) \\&= \sqrt{\mu '}\operatorname {Sdet}_{\operatorname {im}d_{A'}^*}^\frac{1}{2}(*d_{A'}K_{A'}d_{A}) \\&= \sqrt{\mu '}\operatorname {Sdet}_{\operatorname {im}d_{A'}^*}^\frac{1}{2}(*d_{A'}* d_{A'}* G_{A'}d_{A}) \\&= \sqrt{\mu '}\operatorname {Sdet}_{\operatorname {im}d_{A'}^*}^\frac{1}{2}(*d_{A'}* G_{A'} d_{A'}* d_{A})\\&= \sqrt{\mu '}\operatorname {Sdet}_{\operatorname {im}d_{A'}^*}^\frac{1}{2}(K_{A'}d_{A'}*d_{A}) \end{aligned}$$

where we have used that the Green’s function $G_{A'}$ commutes with both the the Hodge star and $d_{A'}$.^46^ For the phase, we note that the spectrum of $K_{A'}d_{A'} * d_{A}$ is obtained from the spectrum of $*d_{A'}$ through continuous deformation where none of the real parts of the eigenvalues crosses zero, therefore any regularization of the signature will yield the same result. □

However, it turns out that we have the following:

##### Lemma 3.7

The expression (110) for $$I_{A,A'}$$ is independent of $A^{\prime }$ and depends on *g* only through $$\psi _{A'}$$. In particular, $$I_{A,A'} = I_{A,A} = \tau ^\frac{1}{2}_Ae^{\frac{i\pi }{4}\psi _A}$$.

The proof is a long computation. Crucially, the non-flatness of $$\nabla ^\textrm{Harm}$$ means that the cohomology comparison map $${\mathfrak {B}}_{A\leftarrow A';A'}$$ depends both on $A^{\prime }$ and *g*, this dependence precisely cancels the dependence of $$\operatorname {Sdet}^{\frac{1}{2}}_{\operatorname {im}d_{A'}^*}\left( 1 + K_{A'}\operatorname {ad}_\beta \right) $$ on $A^{\prime }$ and *g*.

## Properties of the Desynschronized Partition Function

This section is devoted to the proof of Theorem 1.6 which we split up in several subsections. Throughout this section *A* and *A* is a pair of close smooth irreducible flat connections.

### Gauge invariance

We first discuss the impact of gauge transformations on $Z_{A,A'}({\textsf{a}})$. Note that the gauge transformation $A \mapsto {}^\textrm{g}A$ induces an isomorphism $H_{A}^\bullet \cong H_{{}^\textrm{g}A}^\bullet$ by the adjoint action on cohomology classes.

#### Proposition 4.1

We have that $Z_{A,A'}({\textsf{a}})$ is invariant under “diagonal” gauge transformations $(A,A',{\textsf{a}}) \mapsto ({}^\textrm{g}A,{}^\textrm{g}A',{}^\textrm{g}{\textsf{a}})$.

#### Proof

This follows from the fact that all the ingredients of $Z_{A,A'}$ are gauge equivariant. I.e., we have $K_{{}^\textrm{g}A}({}^g\omega ) = {}^\textrm{g}(K_{A}\omega )$ and $\iota _{{}^\textrm{g}A}[{}^g\omega ] = {}^\textrm{g}(\iota _{A}\omega ).$ Finally, we contract tensors using the *G*-invariant pairing on ${\mathfrak {g}}$. □

### Horizontality w.r.t. Grothendieck connection (changing the kinetic operator)

In the next theorem we prove that a shift in the kinetic operator can be expressed as a shift of the zero mode (or vice versa).

Let $\varphi _{A,A'}(\alpha )=A+\delta _{A,A'}(\alpha ):\; U \rightarrow \textrm{FC}'$ be the sum-over-trees exponential map (47), determined by the SDR data associated to the SDR data $r_{\textsf{h}}= (i_{\textsf{h}},p_{\textsf{h}},K_{\textsf{h}})$ corresponding to the gauge-fixing operator ${\textsf{h}}=d^*_{A'}$. The map $\varphi _{A,A'}(\alpha )$, as a function of α, is defined on some open neighborhood *U* of zero in $H^1_{A}$.

In this section we will denote for brevity

111 $$\begin{aligned} \widetilde{A}:= \varphi _{A,A'}(\alpha ). \end{aligned}$$

Denote

112 $$\begin{aligned} B:= {\mathfrak {B}}^1_{\widetilde{A}\leftarrow A;A'}:H^1_{A}\rightarrow H^1_{\widetilde{A}} \end{aligned}$$

the cohomology comparison map in degree 1.

#### Remark 4.2

The map (112) coincides with the differential of $\pi \circ \varphi _{A,A'}(\alpha )$ in the last argument, with $\pi :\textrm{FC}' \rightarrow {\mathcal {M}}'$ the quotient by gauge transformations. This follows from the fact that

113 $$\begin{aligned} \begin{aligned} i_{\widetilde{A},A'}\circ B= &  i_{A,A'}-K_{A,A'}\textrm{ad}_{\delta _{A,A'}(\alpha )}i_{A,A'}+\cdots \\= &  d_\alpha \varphi _{A,A'}(\alpha )\;:H_{A}^1\rightarrow \textrm{Harm}^1_{\widetilde{A},A'}, \end{aligned} \end{aligned}$$

cf. Remarks 2.22, 2.39.

#### Theorem 4.3

We have that the desynchronized partition function satisfies

114 $$\begin{aligned} \det (B^\vee ) \circ Z_{\varphi _{A,A'}(\alpha ),A'}(B({\textsf{a}})) = Z_{A,A'}(\alpha + {\textsf{a}}) \end{aligned}$$

where ${\textsf{a}}$ and α denote variables in an open neighborhood of zero in $H^1_{A}$.

The proof of this theorem relies on the following fact about the dependence of the Ray-Singer torsion on the local system that we were unable to locate in the literature:

#### Proposition 4.4

Ray-Singer torsion satisfies

115 $$\begin{aligned} \det (B^\vee )\circ \tau _{\widetilde{A}}^{1/2}=\tau _{A}^{1/2} \exp \sum _{\gamma } \frac{1}{|\textrm{Aut}(\gamma )|}\Phi _{\gamma ,A,A'}(\alpha ). \end{aligned}$$

Here γ runs over 1-loop connected trivalent graphs.

We give the proof in Appendix D.2.

**Fig. 5:**
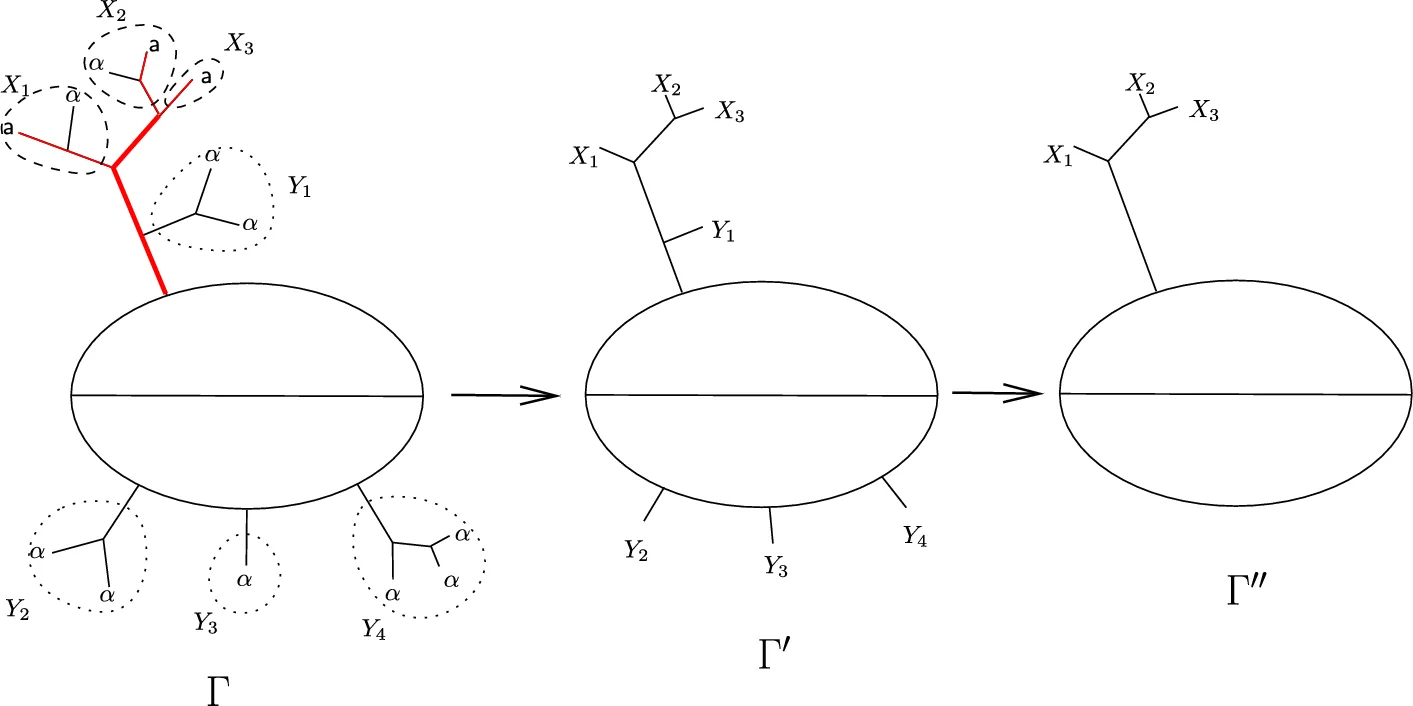
A Feynman graph Γ with *X*- and *Y*-subtrees and the resulting $\Gamma^{\prime }$ and $\Gamma^{\prime \prime }$ graphs. $${\textsf{a}}$$-paths are shown in red; edges belonging to several $${\textsf{a}}$$ paths are thick red edges

#### Sketch of proof of Theorem 4.3

The r.h.s. of (114) is $$e^{\frac{i}{\hbar }S_{CS}(A)} e^{\frac{i}{\hbar }\psi _{A}}\tau _{A}^\frac{1}{2}$$ times the exponential of the sum of connected Feynman graphs Γ with l≥1 loops, with leaves decorated by either $$i_{A,A'}({\textsf{a}})$$ or $$i_{A,A'}(\alpha )$$ and edges decorated by $$K_{A,A'}$$. Given such a graph Γ, one can represent it—in a unique way—as a smaller graph $\Gamma^{\prime }$ with leaves decorated by subtrees $$X_1,\ldots ,X_m$$ and $$Y_1,\ldots ,Y_n$$ of the original graph Γ, where

- Subtrees $X_i$ have a single leaf decorated by ${\textsf{a}}$; all the rest are decorated by α.
- Subtrees $Y_j$ have all leaves decorated by α.
- If a vertex of $\Gamma^{\prime }$ has more than one incident leaves, they must all be decorated by *X*-subtrees.

One can think of $\Gamma^{\prime }$ as Γ with subtrees $$\{X_i\}$$, $$\{Y_j\}$$ collapsed, each tree to its root. We will also denote $\Gamma^{\prime \prime }$ the graph obtained from $\Gamma^{\prime }$ by removing all *Y*-leaves and merging the internal edges incident to them.

Explicit construction of $\Gamma^{\prime }$: Γ can be thought of a trivalent graph $$\widetilde{\Gamma }$$ with no leaves, with several rooted trees $$T_1,\ldots ,T_N$$ plugged into the edges of $$\widetilde{\Gamma }$$. Starting from each leaf of Γ decorated by $${\textsf{a}}$$ (which belongs to some tree $$T_k$$), draw the shortest path along edges connecting it to the root of $$T_k$$, call it an “$${\textsf{a}}$$-path” (Fig. 5). The graph $\Gamma^{\prime }$ is obtained by taking all the edges of Γ which are either (a) non-separating (cutting the edge does not make the graph disconnected) or (b) have at least 2 $${\textsf{a}}$$-paths passing through them; together with each vertex involved we take its neighborhood in Γ, producing leaves. The graph $$\Gamma \setminus \Gamma '$$ is disjoint and consists of *X*-subtrees (those containing an $${\textsf{a}}$$-path) and *Y*-subtrees (those not containing an $${\textsf{a}}$$-path).

The sum over Γ can be represented as a sum over graphs $\Gamma^{\prime \prime }$. Summation over possible subtrees *X* on a leaf of $\Gamma^{\prime \prime }$ yields

116 $$\begin{aligned} i_{\widetilde{A},A'}(B({\textsf{a}}))= i_{A,A'}({\textsf{a}})-K_{A,A'}\textrm{ad}_{\delta _{A,A'}(\alpha )} i_{A,A'}({\textsf{a}})+\cdots , \end{aligned}$$

cf. (113). Summation over inserting k≥0 *Y*-subtrees into an edge *e* of $\Gamma^{\prime \prime }$ results in decorating that edge with the chain homotopy

117 $$\begin{aligned} K_{\widetilde{A},A'} =K_{A,A'}-K_{A,A'}\textrm{ad}_{\delta _{A,A'}(\alpha )} K_{A,A'}+\cdots \end{aligned}$$

Formulae (116), (117) are the homological perturbation theory expressions for the deformation of an SDR data (*i*, *p*, *K*) for the deformation retraction $\Omega (M,{\mathfrak {g}})\rightarrow H_{A}$ induced by a deformation of the differential from $d_{A}$ to $d_{\widetilde{A}}$, see Appendix A for details.

Thus, the sum over Feynman graphs Γ in the r.h.s. of (114) equals the sum over Feynman graphs $\Gamma^{\prime \prime }$ in the l.h.s. of (114). There is one correction: one-loop graphs Γ with leaves decorated only by α (no $${\textsf{a}}$$) were omitted in this correspondence, since they result in $\Gamma^{\prime \prime }$ being a loop with no vertices, which is not a legitimate graph. Thus we have

118 $$\begin{aligned} &  \exp \sum _{\Gamma ''}\frac{(-i\hbar )^{l(\Gamma '')-1}}{|\textrm{Aut}(\Gamma )|}\Phi _{\Gamma '',\widetilde{A},A'}(B({\textsf{a}}))\cdot \exp \sum _{\gamma \;\mathrm {1-loop}} \frac{1}{|\textrm{Aut}(\gamma )|}\Phi _{\gamma ,A,A'}(\alpha ) \nonumber \\ &  \quad =\exp \sum _{\Gamma }\frac{(-i\hbar )^{l(\Gamma )-1}}{|\textrm{Aut}(\Gamma )|}\Phi _{\Gamma ,A,A'}(\alpha +{\textsf{a}}) . \end{aligned}$$

Together with (115) this implies (114). □

#### Corollary 4.5

(Infinitesimal variation of kinetic operator). Let $A_t$ be a curve of flat connections such that ${\dot{A}}_0 = i_{A_0,A'}(\alpha )$ and let $B_t ={\mathfrak {B}}^1_{A_t \leftarrow A_0;A'}:H^1_{A_0}\rightarrow H^1_{A_t}$ be the cohomology comparison map in degree 1. Then

119 $$\begin{aligned} \left. \frac{d}{dt}\right| _{t=0} \Big (\det (B_t^\vee )\circ Z_{A_t,A'}( B_t ({\textsf{a}}))\Big ) = \left\langle \alpha , \frac{\partial }{\partial {\textsf{a}}} \right\rangle Z_{A_0,A'}({\textsf{a}}). \end{aligned}$$

#### Proof 1

Follows immediately from Theorem 4.3 by setting $A_t = \varphi _{A_0,A'}(t \alpha )$ and taking the derivative of both sides in *t* at t=0.

□

One can also prove (119) by a standalone combinatorial argument.

#### Proof 2

One has the following formulae for the infinitesimal variation of *i*, *K*:

120 $$\begin{aligned} \begin{aligned}&\left. \frac{d}{dt}\right| _{t=0}K_{A_t,A'}=-K_{A_0,A'}\textrm{ad}_{i_{A_0,A'}(\alpha )} K_{A_0,A'},\\&\left. \frac{d}{dt}\right| _{t=0} i_{A_t,A'}(B_t({\textsf{a}}))= - K_{A_0,A'}\textrm{ad}_{i_{A_0,A'}(\alpha )} i_{A_0,A'}({\textsf{a}}). \end{aligned} \end{aligned}$$

**Fig. 6:**
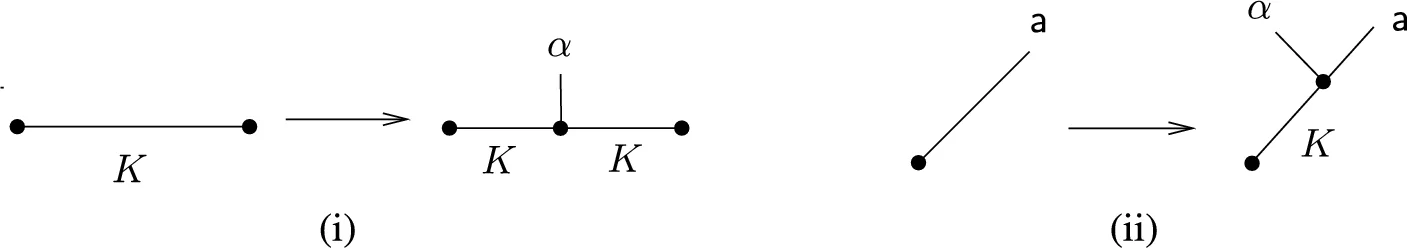
Variation of a Feynman graph Γ under a harmonic shift of *A*: local picture on the graph

These imply that the l.h.s. of (119) is the sum over graphs Γ, where either (i) one edge is split into two by an insertion of leaf decorated by α, or (ii) one ${\textsf{a}}$-leaf is replaced by a subtree consisting of an ${\textsf{a}}$-leaf meeting an α-leaf and continuing with an edge (Fig. 6).

Additionally, one has a special graph—the one-loop graph with a single α-leaf (the “tadpole”), arising from the variation of $\tau _{A_t}^{1/2}$ (Proposition 4.4) It is easy to see that the r.h.s. of (119) yields exactly the same graphs. □

#### Remark 4.6

The r.h.s. of (119) can also be written as a BV-exact term

121 $$\begin{aligned} \Delta _{\textsf{a}}\Big (\langle \alpha , {\textsf{a}}\rangle Z_{A_0,A'}({\textsf{a}})\Big ). \end{aligned}$$

The expression in brackets is a formal half-density on $H^\bullet _{A_0}$ of ghost degree -1. Expanding ${\textsf{a}}={\textsf{a}}^{1}+{\textsf{a}}^{2}$, with ${\textsf{a}}^{k}\in H^k_{A_0}[1-k]$, we can write the expression in brackets as $\langle \alpha , {\textsf{a}}^{2}\rangle Z_{A_0,A'}({\textsf{a}}^{1})$.

Indeed, one has

122 $$\begin{aligned}&\Delta _{\textsf{a}}\Big (\langle \alpha , {\textsf{a}}\rangle Z_{A_0,A'}({\textsf{a}})\Big ) \nonumber \\&\quad = -\underbrace{\Delta _{\textsf{a}}\langle \alpha , {\textsf{a}}\rangle }_0 \cdot Z_{A_0,A'}({\textsf{a}})- \langle \alpha , {\textsf{a}}\rangle \cdot \underbrace{\Delta _{\textsf{a}}Z_{A_0,A'}({\textsf{a}})}_0 - \Big \{\left\langle \alpha , {\textsf{a}}\right\rangle , Z_{A_0,A'}({\textsf{a}})\Big \}\nonumber \\&\quad = \left\langle \alpha , \frac{\partial }{\partial {\textsf{a}}} \right\rangle Z_{A_0,A'}({\textsf{a}}) \end{aligned}$$

– the r.h.s. of (119), as claimed.^47^

Consider the graded vector bundle $\mathcal {D}$ over $\textrm{FC}'$ with fiber over *A* being the space of formal half-densities on cohomology

123 $$\begin{aligned} \mathcal {D}_{A}=\textrm{Dens}^{\frac{1}{2},\textrm{formal}}(H^\bullet _{A}[1])\cong \textrm{Det} (H^1_{A})^* \otimes \widehat{\textrm{Sym}}(H^\bullet _{A}[1])^*. \end{aligned}$$

The flat connection $\nabla ^{{\mathbb {H}},A'}$ in the cohomology bundle (Sect. 2.5.4) induces a flat connection in $\mathcal {D}$ which by abuse of notations we will also denote $\nabla ^{{\mathbb {H}},A'}$.

Let $\textrm{pr}_1$ be the projection onto the first factor in $\textrm{FC}'\times \textrm{FC}'$.

#### Definition 4.7

*(Partial Grothendieck connection)*. One has a partial connection $\widetilde{\nabla }^G$ on the bundle $\textrm{pr}_1^* \mathcal {D}$ over $\mathcal {U}\subset \textrm{FC}'\times \textrm{FC}'$ defined by

124 $$\begin{aligned} (\widetilde{\nabla }^G)_{(\chi ,0)}\xi ({\textsf{a}})=\nabla ^{{\mathbb {H}},A'}_\chi \xi ({\textsf{a}}) -\left\langle [\chi ],\frac{\partial }{\partial {\textsf{a}}} \right\rangle \xi ({\textsf{a}}) \end{aligned}$$

for any $$\chi \in \textrm{Harm}^1_{A,A'}$$ and $$\xi ({\textsf{a}})$$ a section of $$\textrm{pr}_1^* \mathcal {D}$$. Here (χ,0) is a tangent vector to $$\textrm{FC}'\times \textrm{FC}'$$ at a point $\left(A,A^{\prime }\right)$.

Thus (124) allows to differentiate sections of $\textrm{pr}_1^* \mathcal {D}$ in the direction of infinitesimal harmonic shifts of *A*.

#### Remark 4.8

The partial connection (124) is induced (via pushforward of half-densities) from the fiber bundle partial connection on the cohomology bundle $\textrm{pr}_1^* {\mathbb {H}}$ over $\mathcal {U}$ defined by the parallel transport

125 $$\begin{aligned} \begin{array}{ccc} H_A^\bullet =\textrm{pr}_1^* {\mathbb {H}}|_{A,A'} & \rightarrow & H^\bullet _{\widetilde{A}}=\textrm{pr}_1^* {\mathbb {H}}|_{\widetilde{A},A'} \\ {\textsf{a}}& \mapsto & \widetilde{{\textsf{a}}}={\mathfrak {B}}_{\widetilde{A}\leftarrow A,A'} ({\textsf{a}}-\alpha ) \end{array} \end{aligned}$$

with $\alpha \in H^1_A$ sufficiently small and $\widetilde{A}$ as in (111). We remark that the parallel transport (125) satisfies

126 $$\begin{aligned} \varphi _{A,A'}({\textsf{a}})=\varphi _{\widetilde{A},A'}(\widetilde{{\textsf{a}}}). \end{aligned}$$

for ${\textsf{a}}\in H^1_A$ sufficiently small.

#### Corollary 4.9

(Horizontality with respect to the partial Grothendieck connection). One has

127 $$\begin{aligned} {\widetilde{\nabla }}^G Z_{A,A'} = 0. \end{aligned}$$

This is just a rephrasing of Corollary 4.5.

### Changing the gauge-fixing operator

#### Theorem 4.10

(Changing the gauge-fixing operator). We have that, for $A_0'$ and $A'_1$ flat connections close to a flat connection *A*

128 $$\begin{aligned} Z_{A,A_1'}({\textsf{a}}) = Z_{A,A'_0}({\textsf{a}}) -i\hbar \Delta _{\textsf{a}}R_{A,A'_0,A'_1}({\textsf{a}}). \end{aligned}$$

with $R_{A,A'_0,A'_1}({\textsf{a}})$ a formal half-density on $H^\bullet _A[1]$ given by (132) below.

This is an immediate consequence of Proposition 4.11 below, by integrating over a path $A'_t$ from $A'_0$ to $A'_1$.

Let us consider the effect of an infinitesimal change of $A'\rightarrow A'+\delta A'$ on $Z_{A,A'}$. Consider the following endomorphisms of $\Omega ^\bullet (M,{\mathfrak {g}})$:

129 $$\begin{aligned} \Lambda = K_{A,A'} \textrm{ad}^*_{\delta A'} G_{A,A'}, \quad {\mathbb {I}} = G_{A,A'} \textrm{ad}^*_{\delta A'}, \quad {\mathbb {P}} = \textrm{ad}^*_{\delta A'} G_{A,A'}. \end{aligned}$$

They arise in the first-order deformation of the SDR data $$(i,p,K)_{A,A'}$$ resulting from the deformation of $A^{\prime }$:

130 $$\begin{aligned} \begin{aligned} \delta _{A'} K_{A,A'}=&[d_A,\Lambda ]+P_{A,A'}{\mathbb {P}} +{\mathbb {I}} P_{A,A'}, \\ \delta _{A'} i_{A,A'} =&-d_A{\mathbb {I}} i_{A,A'},\\ \delta _{A'} p_{A,A'} =&-p_{A,A'}{\mathbb {P}} d_A, \end{aligned} \end{aligned}$$

cf. Lemma A.3. Here $$P_{A,A'}=i_{A,A'}p_{A,A'}$$ is the projection onto $\left(A,A^{\prime }\right)$-harmonic forms. We note that $${\mathbb {I}}$$ and $${\mathbb {P}}$$ are mutually transpose w.r.t. Poincaré pairing on forms and cohomology, while Λ is symmetric w.r.t. Poincaré pairing.

#### Proposition 4.11

For $$A,A'\in \textrm{FC}'$$ a pair of close flat connections, the variation of $$Z_{A,A'}({\textsf{a}})$$ with respect to variation of $A^{\prime }$ is given by

131 $$\begin{aligned} \delta _{A'} Z_{A,A'}({\textsf{a}})= -i\hbar \Delta _{\textsf{a}}\left( r_{A,A';\delta A'}({\textsf{a}}) Z_{A,A'}({\textsf{a}})\right) \end{aligned}$$

where $r_{A,A';\delta A'}({\textsf{a}})$ is the sum of connected Feynman graphs with one marked edge decorated by Λ or one marked leaf decorated by ${\mathbb {I}}$.

Note that if $A'_t$ is a path from $A'_0$ to $A'_1$, integrating (131) we obtain (128) with

132 $$\begin{aligned} R_{A,A'_0,A'_1}({\textsf{a}})=\int _0^1 dt\, r_{A,A'_t;{\dot{A}}'_t}({\textsf{a}}) Z_{A,A'_t}({\textsf{a}}). \end{aligned}$$

#### Proof of Proposition 4.11

Denote $Z^0_A=e^{\frac{i}{\hbar }S_{CS}(A)}e^{\frac{\pi i}{4}\psi _A}\tau _A^{\frac{1}{2}}$. We have

133 $$\begin{aligned} (Z^0_A)^{-1} \delta _{A'} Z_{A,A'}({\textsf{a}}) = \sum _{\Gamma } \delta _{A'}\overline{\Phi }_{\Gamma ,A,A'}({\textsf{a}}) \end{aligned}$$

– a sum over possibly disconnected trivalent graphs with leaves allowed, where we denoted

$$\begin{aligned} \overline{\Phi }_{\Gamma ,A,A'}({\textsf{a}})=\underbrace{\frac{(-i\hbar )^{-\chi (\Gamma )}}{|\textrm{Aut}(\Gamma )|}}_{c_\Gamma }\Phi _{\Gamma ,A,A'}({\textsf{a}})=\int _{\overline{\textrm{Conf}}_V(M)} \omega _\Gamma . \end{aligned}$$

Here $$\omega _\Gamma $$ is the integrand of (99) in $\left(A,A^{\prime }\right)$-gauge, normalized with an appropriate power of ħ and a combinatorial factor:

134 $$\begin{aligned} \omega _\Gamma =c_\Gamma \Big \langle \bigwedge _{e=(uv)\in E} \pi ^*_{uv} \eta \wedge \bigwedge _{l\in L} \pi ^*_{v(l)}i({\textsf{a}}),\bigotimes _{v\in V}f \Big \rangle . \end{aligned}$$

Here:

- *V*, *E*, *L* are the sets of vertices, edges and leaves of Γ.
- For a short loop e=(vv), expression $\pi _{vv}^*\eta$ is interpreted as $\pi _v^* \eta ^\Delta$ with $\eta ^\Delta$ the regularized diagonal evaluation of η.
- $A,A^{\prime }$ subscripts are suppressed.

We will also use shorthand notation $\eta _e= \pi ^*_{uv}\eta$, $i({\textsf{a}})_l=\pi _{v(l)}^* i({\textsf{a}})$.

The rest of the proof is parallel to the arguments of [CM08, Section 4.5, Appendix A], [CMR17, Lemma 4.11].^48^

Variation of the value of a Feynman graph in $A^{\prime }$ is the sum over edges and leaves of the graph of replacing that edge or leaf with its variation:

135 $$\begin{aligned} &  \delta _{A'}{\overline{\Phi }}_{\Gamma ,A,A'} \nonumber \\ &  \quad =\sum _{e_\textrm{m}\in E} \int _{\overline{\textrm{Conf}}_V(M)} c_\Gamma \Big \langle \delta _{A'}\eta _{e_\textrm{m}}\wedge \bigwedge _{e\in E\setminus e_\textrm{m}}\eta _e\wedge \bigwedge _{l\in L}i({\textsf{a}})_l,\bigotimes _{v\in V} f \Big \rangle \nonumber \\ &  \qquad + \sum _{l_\textrm{m}\in L} \int _{\overline{\textrm{Conf}}_V(M)} c_\Gamma \Big \langle \bigwedge _{e\in E}\eta _e\wedge \delta _{A'}i({\textsf{a}})_{l_\textrm{m}} \wedge \bigwedge _{l\in L\setminus l_\textrm{m}}i({\textsf{a}})_l,\bigotimes _{v\in V} f \Big \rangle . \end{aligned}$$

(Subscript $\textrm{m}$ in $e_\textrm{m},l_\textrm{m}$ is for “marked” edge/leaf.) We introduce the following notation: for $O\in \{\Lambda , P,\mathbb {I} P, P{\mathbb {P}}\}$ an operator on $\Omega ^\bullet (M,{\mathfrak {g}})$, we will denote $\eta _O \in \Omega ^p(\overline{\textrm{Conf}}_2(M),{\mathfrak {g}}\otimes {\mathfrak {g}})$ its integral kernel, seen as a form on the compactified configuration space of two points of degree p=1 for Λ, p=2 for $\mathbb {I} P$ and $P{\mathbb {P}}$, p=3 for *P*. From (130) we have

136 $$\begin{aligned} \delta _{A'} \eta = -d\eta _\Lambda + \eta _{\mathbb {I} P}+ \eta _{P {\mathbb {P}}}, \quad \delta _{A'}i({\textsf{a}})=-d{\mathbb {I}}i({\textsf{a}}). \end{aligned}$$

Note that operators $\mathbb {I} P$ and $P{\mathbb {P}}$ are mutually transpose.

**Fig. 7:**
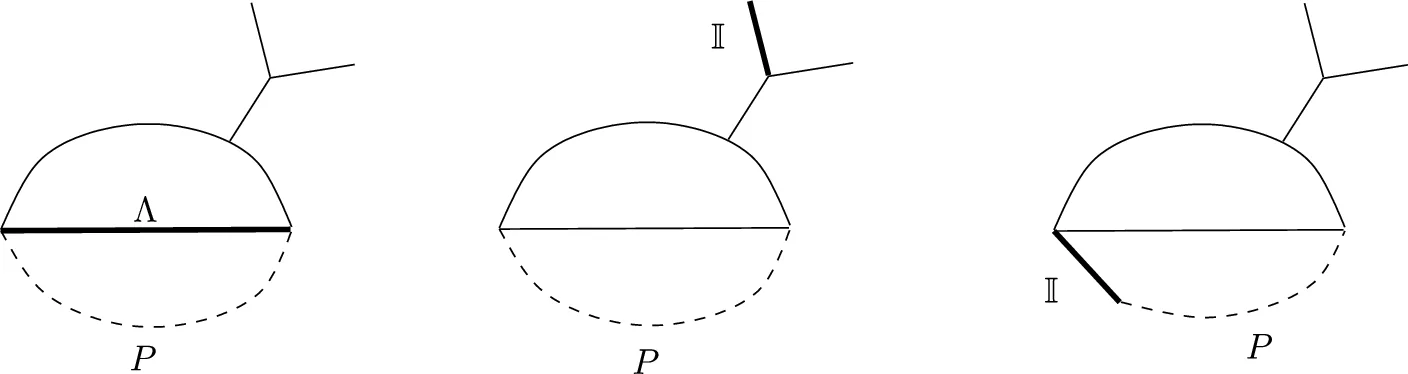
Examples of graphs contributing to (137), (138), (139)

Next, we substitute (136) in (135) and use Stokes’ theorem on the configuration space to move *d* from the marked edge $e_\textrm{m}$ or leaf $l_\textrm{m}$ to another edge $e'_\textrm{m}$ (note that^49^$d\eta =\eta _P$, since [d,K]=1-P). In the process, Stokes’ theorem produces additional contributions of the boundary of the configuration space (see also Fig. 7).

137 $$\begin{aligned}&\delta _{A'} {\overline{\Phi }}_{\Gamma ,A,A'}= \nonumber \\&\sum _{e_\textrm{m}\ne e'_\textrm{m}\in E}\int _{\overline{\textrm{Conf}}_V(M)} c_\Gamma \cdot \nonumber \\&\cdot \Big \langle (\eta _P)_{e'_\textrm{m}}\wedge (\eta _\Lambda )_{e_\textrm{m}}\wedge \bigwedge _{e\in E\setminus \{e_\textrm{m},e'_\textrm{m}\}}\eta _e \wedge \bigwedge _{l\in L} i({\textsf{a}})_l,\bigotimes _{v\in V} f \Big \rangle \end{aligned}$$

138 $$\begin{aligned}&+\sum _{l_\textrm{m}\in L,\, e'_\textrm{m}\in E} \int _{\overline{\textrm{Conf}}_V(M)} c_\Gamma \cdot \nonumber \\&\cdot \Big \langle (\eta _P)_{e'_\textrm{m}} \wedge \bigwedge _{e\in E\setminus e'_\textrm{m}}\eta _e \wedge \mathbb {I} i({\textsf{a}})_{l_\textrm{m}}\wedge \bigwedge _{l\in L\setminus l_\textrm{m}} i({\textsf{a}})_l, \bigotimes _{v\in V} f\Big \rangle \end{aligned}$$

139 $$\begin{aligned}&+\sum _{e_\textrm{m}\in E} \int _{\overline{\textrm{Conf}}_V(M)} c_\Gamma \cdot \nonumber \\&\cdot \Big \langle (\eta _{\mathbb {I} P}+\eta _{P{\mathbb {P}}})_{e_\textrm{m}}\wedge \bigwedge _{e\in E\setminus e_\textrm{m}}\eta _e\wedge \bigwedge _{l\in L}i({\textsf{a}})_l,\bigotimes _{v\in V} f \Big \rangle \end{aligned}$$

140 $$\begin{aligned}&+\sum _{e_\textrm{m}\in E} \int _{\partial \overline{\textrm{Conf}}_V(M)} c_\Gamma \cdot \nonumber \\&\cdot \Big \langle (\eta _\Lambda )_{e_\textrm{m}}\wedge \bigwedge _{e\in E\setminus e_\textrm{m}}\eta _e\wedge \bigwedge _{l\in L}i({\textsf{a}})_l,\bigotimes _{v\in V} f \Big \rangle \end{aligned}$$

141 $$\begin{aligned}&+ \sum _{l_\textrm{m}\in L} \int _{\partial \overline{\textrm{Conf}}_V(M)} c_\Gamma \cdot \nonumber \\&\cdot \Big \langle \bigwedge _{e\in E}\eta _e\wedge \mathbb {I} i({\textsf{a}})_{l_\textrm{m}} \wedge \bigwedge _{l\in L\setminus l_\textrm{m}}i({\textsf{a}})_l,\bigotimes _{v\in V} f \Big \rangle . \end{aligned}$$

The boundary contributions here (140), (141) cancel out in the sum over graphs Γ as follows. The boundary of the configuration space is stratified (see [AS94, CM08]) into “principal strata” (differential geometric blow-ups of collapses of two points on *M*) and “hidden strata” (collapses of ≥3 points). Contributions of principal strata cancel out in the sum over graphs Γ as a consequence of Jacobi identity (or, equivalently, as a consequence of the classical master equation on BV Chern–Simons action).

Hidden boundary strata of the configuration space do not contribute to the integrals in (140), (141); we defer the proof of this to Appendix D.3.

Denote

142 $$\begin{aligned} \zeta&= \sum _{\Gamma ^\#} \sum _{e_\textrm{m}\in E^\#} \int _{\overline{\textrm{Conf}}_{V^\#}(M)} c_{\Gamma ^\#} \Big \langle (\eta _\Lambda )_{e_\textrm{m}}\wedge \bigwedge _{e\in E^\#\setminus e_\textrm{m}}\eta _e\wedge \bigwedge _{l\in L^\#} i({\textsf{a}})_l,\bigotimes _{v\in V^\#} f \Big \rangle \nonumber \\&\quad +\sum _{\Gamma ^\#} \sum _{l_\textrm{m}\in L^\#} \int _{\overline{\textrm{Conf}}_{V^\#}(M)} c_{\Gamma ^\#} \Big \langle \bigwedge _{e\in E^\#}\eta _e\wedge \mathbb {I} i({\textsf{a}})_{l_\textrm{m}} \wedge \bigwedge _{l\in L^\#\setminus l_\textrm{m}} i({\textsf{a}})_l,\bigotimes _{v\in V^\#} f \Big \rangle . \end{aligned}$$

Here $\Gamma ^\#$ runs over possibly disconnected trivalent graphs with leaves allowed; $V^\#,E^\#,L^\#$ stand for the sets of vertices/edges/leaves of $\Gamma ^\#$.

Note that

143 $$\begin{aligned} \zeta =(Z^0_A)^{-1} r_{A,A';\delta A'}({\textsf{a}}) Z_{A,A'}({\textsf{a}}) \end{aligned}$$

with *r* defined as in Proposition 4.11.

On the other hand, applying $\Delta _{\textsf{a}}$ to each term in (142) yields a sum over pairs of $i({\textsf{a}})$ leaves, of replacement of that pair by a new edge adjoined to the graph $\Gamma ^\#$ and decorated by *P*:

144 $$\begin{aligned}&\Delta _{\textsf{a}}\zeta \nonumber \\&\quad =\sum _{\Gamma ^\#} \sum _{e_\textrm{m}\in E^\#,\, l_\textrm{m}\ne l'_\textrm{m}\in L^\#} \int _{\overline{\textrm{Conf}}_{V^\#}(M)} c_{\Gamma ^\#}\cdot \end{aligned}$$

145 $$\begin{aligned}&\qquad \cdot \Big \langle (\eta _P)_{(v(l_\textrm{m}) v(l'_\textrm{m}))} \wedge (\eta _\Lambda )_{e_\textrm{m}}\wedge \bigwedge _{e\in E^\#\setminus e_\textrm{m}}\eta _e\wedge \bigwedge _{l\in L^\#\setminus \{l_\textrm{m},l'_\textrm{m}\}} i({\textsf{a}})_l,\bigotimes _{v\in V^\#} f \Big \rangle \nonumber \\&\qquad + \sum _{\Gamma ^\#} \sum _{l_\textrm{m},l'_\textrm{m},l''_\textrm{m}\in L^\#\,\textrm{distinct}} \int _{\overline{\textrm{Conf}}_{V^\#}(M)} c_{\Gamma ^\#}\cdot \end{aligned}$$

146 $$\begin{aligned}&\qquad \cdot \Big \langle \bigwedge _{e\in E^\#}\eta _e\wedge \mathbb {I} i({\textsf{a}})_{l_\textrm{m}} \wedge (\eta _P)_{(v(l'_\textrm{m}) v(l''_\textrm{m}))} \wedge \bigwedge _{l\in L^\#\setminus \{l_\textrm{m},l'_\textrm{m},l''_\textrm{m}\}} i({\textsf{a}})_l,\bigotimes _{v\in V^\#} f \Big \rangle \nonumber \\&\qquad +\sum _{\Gamma ^\#} \sum _{l_\textrm{m}\ne l'_\textrm{m}\in L^\#} \int _{\overline{\textrm{Conf}}_{V^\#}(M)} c_{\Gamma ^\#} \cdot \nonumber \\&\qquad \cdot \Big \langle \bigwedge _{e\in E^\#}\eta _e\wedge (\eta _{\mathbb {I} P})_{(v(l_\textrm{m}) v(l'_\textrm{m}))} \wedge \bigwedge _{l\in L^\#\setminus \{l_\textrm{m}, l'_\textrm{m}\} } i({\textsf{a}})_l,\bigotimes _{v\in V^\#} f \Big \rangle . \end{aligned}$$

Comparing with the computation of $\delta _{A'}{\overline{\Phi }}_{\Gamma ,A,A'}$, we find that:

- $\sum _\Gamma$ (137) =-iħ (144). Here Γ is $\Gamma ^\#$ with a new *P*-edge $e'_\textrm{m}=(v(l_\textrm{m}) v(l'_\textrm{m}))$ attached. The extra -iħ factor appears because attaching a new *P*-edge to $\Gamma ^\#$ changes its Euler characteristic by -1 and thus changes the power of (-iħ) in $c_\Gamma$ by +1.
- $\sum _\Gamma$ (138) =-iħ (145). Here Γ is $\Gamma ^\#$ with a new *P*-edge $e'_\textrm{m}=(v(l'_\textrm{m})v(l''_\textrm{m}))$ attached.
- $\sum _\Gamma$ (139) =-iħ (146). Here Γ is $\Gamma ^\#$ with a new $\mathbb {I} P$-edge attached, $e_\textrm{m}=(v(l_\textrm{m})v(l'_\textrm{m}))$, or new $P{\mathbb {P}}$-edge attached, $e_\textrm{m}=(v(l'_\textrm{m})v(l_\textrm{m}))$.

Therefore we have

147 $$\begin{aligned} \underbrace{\sum _\Gamma \delta _{A'} {\overline{\Phi }}_{\Gamma ,A,A'}}_{(Z^0_A)^{-1}\delta _{A'} Z_{A,A'}({\textsf{a}})}=\underbrace{-i\hbar \Delta _{\textsf{a}}\zeta }_{i\hbar (Z^0_A)^{-1} \Delta _{\textsf{a}}(r_{A,A';\delta A'}({\textsf{a}})Z_{A,A'}({\textsf{a}}))} \end{aligned}$$

Multiplying both sides by $Z^0_A$, we have (131). □

#### Total horizontality (modulo Δ-exact terms) on $\textrm{FC}\times \textrm{FC}$

One can summarize the results of Propositions 4.11, 4.1 and Corollary 4.9 as follows. One has splitting of the tangent bundle of $\mathcal {U}\subset \textrm{FC}'\times \textrm{FC}'$ into a direct sum of three integrable distributions

148 $$\begin{aligned} T\mathcal {U}=T^I\oplus T^{II} \oplus T^{III}. \end{aligned}$$

Here:

- $T^I_{A,A'}=\{(\chi ,0)\;|\; \chi \in \mathrm {Harm^1_{A,A'}}\}$—harmonic shifts of *A*.
- $$T^{II}_{A,A'}=\{(0,\gamma )\;|\; \gamma \in \Omega ^1_{A'\mathrm {-closed}}\}$$—shifts of $A^{\prime }$.
- $$T^{III}_{A,A'}=\{(d_A\beta ,d_{A'}\beta )\;|\; \beta \in \Omega ^0\}$$—diagonal gauge transformations of $\left(A,A^{\prime }\right)$.

For the bundle $$\mathcal {D}$$ of formal half-densities on $$H_A$$ over $$\mathcal {U}$$, one has three flat partial connections $$\widetilde{\nabla }^G$$ (124), $$\nabla _{A'}=\delta _{A'}$$, $$\nabla ^\textrm{gauge}$$ along these three distributions. Here $$\nabla ^\textrm{gauge}$$ is such that its parallel transport takes a formal half-density $$\psi ({\textsf{a}})$$ over $\left(A,A^{\prime }\right)$ to the half-density $$\psi (\textrm{g}{\textsf{a}}\textrm{g}^{-1})$$ over $$({}^\textrm{g}A,{}^\textrm{g}A')$$ for any $$\textrm{g}:M\rightarrow G$$. These three partial connections can be assembled into a total flat connection

149 $$\begin{aligned} \nabla ^\textrm{tot}= \widetilde{\nabla }^G+\nabla _{A'}+\nabla ^\textrm{gauge}. \end{aligned}$$

The extended partition function then satisfies the total horizontality equation (modulo Δ-exact terms):

150 $$\begin{aligned} \nabla ^\textrm{tot} Z= -i\hbar \Delta _{\textsf{a}}\Big ( r_{{\widetilde{\delta A'}}}({\textsf{a}}) Z_{A,A'}({\textsf{a}})\Big ). \end{aligned}$$

Here $\delta A^{\prime }$ is replaced in *r* (as in Proposition 4.11) with the expression

151 $$\begin{aligned} \widetilde{\delta A'}=\delta A'-d_{A'}K_{A,A'}\delta A \end{aligned}$$

– a 1-form on $$\mathcal {U}$$ valued in $$\Omega ^1(M,{\mathfrak {g}})$$ which vanishes along $$T^I$$ and $$T^{III}$$ and coincides with $\delta A^{\prime }$ on $$T^{II}$$. Put another way, $$\widetilde{\delta A'}$$ is the projector onto $$T^{II}$$ in (148).

### Extension of $$ Z_{A,A'}$$ to a horizontal nonhomogeneous form in $A^{\prime }$

In this section we describe a refinement of Proposition 4.11: one combines the partition function $$Z_{A,A'}({\textsf{a}})$$ with the BV generator in the r.h.s. of (131) into a nonhomogeneous form in $A^{\prime }$

$$\begin{aligned} \widehat{Z}_{A,A'}({\textsf{a}})= Z_{A,A'}({\textsf{a}})+ r({\textsf{a}}) Z_{A,A'}({\textsf{a}})+\underbrace{\cdots }_{\textrm{degree}\;\ge 2\;\textrm{in}\; A'} \end{aligned}$$

so that the full object satisfies horizontality condition

152 $$\begin{aligned} \widehat{\nabla }_{A'} \widehat{Z}_{A,A'}({\textsf{a}})=0 \end{aligned}$$

with respect to the flat partial superconnection^50^

153 $$\begin{aligned} \widehat{\nabla }_{A'}=\delta _{A'}-i\hbar \Delta _{{\textsf{a}}} \end{aligned}$$

in the direction of $A^{\prime }$ in the bundle of formal half-densities in $$H_A$$ over $$\mathcal {U}\subset \textrm{FC}'\times \textrm{FC}'$$. In particular, in degree zero in $A^{\prime }$, (152) is the equation $$\Delta _{\textsf{a}}Z_{A,A'}({\textsf{a}})=0$$ (the BV quantum master equation) and in degree one in $A^{\prime }$, it yields the Eq. (131).

We proceed to the detailed construction.

Consider the following quadruple of nonhomogeneous forms in $A^{\prime }$ valued in linear maps:

154 $$\begin{aligned} \begin{aligned} \widehat{i}&=\sum _{k\ge 0} (-G_{A,A'}\textrm{ad}^*_{\delta A'})^k i_{A,A'}=i+{\mathbb {I}}i+\cdots \quad \in \Omega ^{0,\bullet }(\mathcal {U},\textrm{Hom}(H_A^\bullet ,\Omega ^\bullet (M,{\mathfrak {g}}))), \\ \widehat{p}&= \sum _{k\ge 0} p_{A,A'}(-\textrm{ad}^*_{\delta A'}G_{A,A'})^k=p+p{\mathbb {P}}+\cdots \quad \in \Omega ^{0,\bullet }(\mathcal {U},\textrm{Hom}(\Omega ^\bullet (M,{\mathfrak {g}}),H_A^\bullet )), \\ \widehat{K}&= \sum _{k\ge 0} K_{A,A'}(-\textrm{ad}^*_{\delta A'}G_{A,A'})^k=K+\Lambda +\cdots \quad \in \Omega ^{0,\bullet }(\mathcal {U},\textrm{End}(\Omega ^\bullet (M,{\mathfrak {g}}))),\\ \widehat{\Theta }&=p_{A,A'}\textrm{ad}^*_{\delta A'} d_A G_{A,A'}(-G_{A,A'}\textrm{ad}^*_{\delta A'}) i_{A,A'}\quad \in \Omega ^{0,2}(\mathcal {U},\textrm{End}(H^\bullet _A)). \end{aligned} \end{aligned}$$

Here for the first three maps, the 0-form component in $A^{\prime }$ is given by the usual maps *i*, *p*, *K* in $\left(A,A^{\prime }\right)$-gauge and the 1-form component is given by the maps $${\mathbb {I}}i$$, $$p{\mathbb {P}}$$, Λ, with $${\mathbb {I}},{\mathbb {P}},\Lambda $$ as in (129). In $$\Omega ^{i,j}(\mathcal {U})$$, (*i*, *j*) is the form bi-degree along the two factors in $$\textrm{FC}'\times \textrm{FC}'$$.

We remark that sums in (154) are finite (stop at k=2) since each factor $\textrm{ad}^*_{\delta A'}$ drops the form degree on *M* by one.

The triple $$(\widehat{i},\widehat{p},\widehat{K})$$ above can be thought of as a promotion of the (*i*, *p*, *K*) triple (SDR data) associated to the $\left(A,A^{\prime }\right)$-gauge-fixing to a *differential family* over $$A'\in \textrm{FC}'$$, cf. Lemma 4.14 below.

#### Definition 4.12

We define the extended desynchronized Chern–Simons perturbative partition function as

155 $$\begin{aligned} &  \widehat{Z}_{A,A'}({\textsf{a}}) =e^{\frac{i}{\hbar }S_{CS}(A)} e^{\frac{\pi i}{4}\psi _A}\tau _{A}^{1/2} e^{\frac{i}{\hbar }\,\frac{1}{2}\langle {\textsf{a}}, \widehat{\Theta }({\textsf{a}})\rangle }\;\exp \sum _\Gamma \frac{(-i\hbar )^{l(\Gamma )-1}}{|\textrm{Aut}(\Gamma )|}\widehat{\Phi }_{\Gamma ,A,A'}({\textsf{a}}) \nonumber \\ &  \quad \in \Omega ^{0,\bullet }(\mathcal {U},\textrm{Dens}^{\frac{1}{2},\textrm{formal}}(H_A[1])) \end{aligned}$$

where Γ runs over connected graphs with l≥0 loops and Feynman weights of graphs are defined as in (99), where we replace *K* with $\widehat{K}$ and *i* with $\widehat{i}$.

#### Theorem 4.13

We have the horizontality relation

156 $$\begin{aligned} \widehat{\nabla }_{A'}\widehat{Z}_{A,A'}({\textsf{a}})=0, \end{aligned}$$

with $\widehat{\nabla }_{A'}$ as in (153).

An alternative name for Eq. (156) is the *differential quantum master equation*, cf. [BCM12].

The proof is based on the fact that the triple $$(\widehat{i},\widehat{p},\widehat{K})$$ satisfies the the relations of an (*i*, *p*, *K*) triple, with the differential $$d_A$$ replaced by the total differential $$\delta _{A'}+d_A$$. Interestingly, in these relations cohomology $$H_A$$ as a family over $A^{\prime }$ attains a nontrivial total differential^51^$$\delta _{A'}+\widehat{\Theta }$$, with $$\widehat{\Theta }$$ of mixed degree along $$\mathcal {U}$$ and along $$H_A$$ but of total degree one.

#### Lemma 4.14

The triple $(\widehat{i},\widehat{p},\widehat{K})$ satisfies the following relations:

157a $$\begin{aligned} (\delta _{A'}+[d_A,-])\widehat{K}= &  1-\widehat{i}\,\widehat{p}, \end{aligned}$$

157b $$\begin{aligned} (\delta _{A'}+d_A)\widehat{i}= &  \widehat{i}\, \widehat{\Theta }, \end{aligned}$$

157c $$\begin{aligned} \delta _{A'} \widehat{p} - \widehat{p}\, d_A= &  -\widehat{\Theta }\, \widehat{p}, \end{aligned}$$

157d $$\begin{aligned} \widehat{K}\, \widehat{i}= &  0, \end{aligned}$$

157e $$\begin{aligned} \widehat{p}\, \widehat{K}= &  0, \end{aligned}$$

157f $$\begin{aligned} \widehat{K}^2= &  0, \end{aligned}$$

157g $$\begin{aligned} \widehat{p} \,\widehat{i}= &  1. \end{aligned}$$

Formulae (154) and Lemma 4.14 are an application of homological perturbation lemma, see Appendix C.

#### Remark 4.15

As an immediate consequence of Lemma 4.14 we have that $\widehat{\Theta }$ satisfies

158 $$\begin{aligned} (\delta _{A'}+\widehat{\Theta })^2=0, \end{aligned}$$

or, equivalently, $\widehat{\Theta }$ satisfies the Maurer-Cartan equation

159 $$\begin{aligned} \delta _{A'}\widehat{\Theta }+\widehat{\Theta }^2=0. \end{aligned}$$

Indeed, one has

$$\begin{aligned} &  \widehat{\Theta }^2\underset{(157g)}{=}\widehat{p}\widehat{i}\widehat{\Theta }\widehat{\Theta }\underset{(157b)}{=}\widehat{p} \big ((\delta _{A'}+d_A)\widehat{i}\big )\widehat{\Theta }= \widehat{p} (\delta _{A'}+d_A)(\widehat{i}\,\widehat{\Theta })-\underbrace{\widehat{p}\,\widehat{i}}_1\delta _{A'}\widehat{\Theta }\\ &  \quad \underset{(157b)}{=} \widehat{p}\underbrace{(\delta _{A'}+d_A)^2}_0\widehat{i}-\delta _{A'}\widehat{\Theta }=-\delta _{A'}\widehat{\Theta }. \end{aligned}$$

#### Proof of Theorem 4.13

The proof is similar to the proof of Proposition 4.11.

Consider the Feynman graph part of $\widehat{Z}$, $\sum _\Gamma \widehat{\Phi }_\Gamma$, with Γ running over possibly disconnected graphs, with edges decorated by $\widehat{K}$ and leaves decorated by $\widehat{i}({\textsf{a}})$. (We include powers of ħ and the symmetry factor in $\widehat{\Phi }$.) The action of $\delta _{A'}$ on $\widehat{\Phi }_\Gamma$ can be computed as a sum of (a) decorations of one edge of Γ with $(\delta _{A'}+[d_A,-])\widehat{K}=1-\widehat{i}\,\widehat{p}$ (157a), plus (b) decorations of one leaf of Γ with $(\delta _{A'}+d_A)\widehat{i}=\widehat{i}\widehat{\Theta }$ (157b). Upon summing over graphs, contributions of 1 cancel out;^52^ contributions of $\widehat{i}\widehat{p}$ yield $-i\hbar \Delta _{\textsf{a}}\sum _\Gamma \widehat{\Phi }_\Gamma$; contributions of $\widehat{i}\widehat{\Theta }$ add up to

$$\begin{aligned} -\Big \{\frac{1}{2} \langle {\textsf{a}},\widehat{\Theta }({\textsf{a}}) \rangle ,\sum _\Gamma \widehat{\Phi }_\Gamma \Big \}. \end{aligned}$$

Thus, we have

$$ (\delta _{A'}-i\hbar \Delta _{\textsf{a}}) \sum _\Gamma \widehat{\Phi }_\Gamma =-\Big \{\frac{1}{2} \langle {\textsf{a}},\widehat{\Theta }({\textsf{a}}) \rangle ,\sum _\Gamma \widehat{\Phi }_\Gamma \Big \}. $$

Next, we have

$$\begin{aligned} &  (\delta _{A'}-i\hbar \Delta _{\textsf{a}})\Big (e^{\frac{i}{\hbar }\,\frac{1}{2}\langle {\textsf{a}},\widehat{\Theta }({\textsf{a}}) \rangle } \sum _\Gamma \widehat{\Phi }_\Gamma \Big ) \\ &  \quad =e^{\frac{i}{\hbar }\,\frac{1}{2}\langle {\textsf{a}},\widehat{\Theta }({\textsf{a}}) \rangle }\frac{i}{\hbar }\Big (\frac{1}{2}\langle {\textsf{a}},(\delta _{A'}\widehat{\Theta }+\widehat{\Theta }^2){\textsf{a}}\rangle + \Big \{\frac{1}{2}\langle {\textsf{a}},\widehat{\Theta }({\textsf{a}}) \rangle ,-\Big \} \\ &  \qquad -\Big \{\frac{1}{2}\langle {\textsf{a}},\widehat{\Theta }({\textsf{a}}) \rangle ,-\Big \} \Big ) \sum _\Gamma \widehat{\Phi }_\Gamma =0, \end{aligned}$$

where we used (159). This proves the horizontality Eq. (156). □

#### A path integral formula for $\widehat{Z}$

**Fig. 8:**
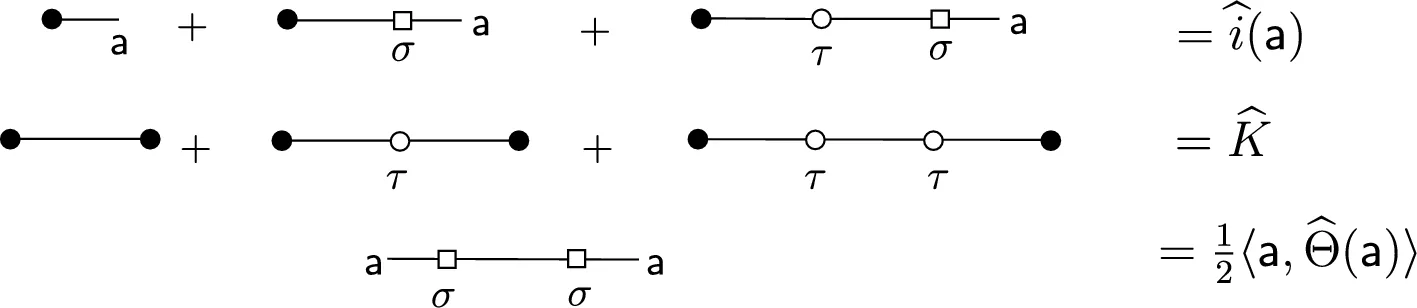
Feynman diagrams containing vertices τ and σ. Black dots are the usual internal vertices of Chern–Simons graphs

The extended partition function (155) can be seen as a perturbative expansion of the following path integral:

160 $$\begin{aligned} \widehat{Z}_{A,A'}({\textsf{a}})= &  \int _{\mathcal {L}=\Omega _{d^*_{A'}\mathrm {-ex}}[1]}\mathcal {D}\alpha _\textrm{fl} \; \exp \frac{i}{\hbar }\Big ( S_{CS}(A+i({\textsf{a}})+\alpha _\textrm{fl}) \nonumber \\ &  +\int _M\frac{1}{2} \underbrace{\big \langle \alpha _\textrm{fl}, d_A G\,\textrm{ad}^*_{\delta A'}\alpha _\textrm{fl} \big \rangle }_\tau + \underbrace{\big \langle \alpha _\textrm{fl},d_A G\,\textrm{ad}^*_{\delta A'} i({\textsf{a}}) \big \rangle }_\sigma \Big ) \end{aligned}$$

where we suppress the indices in $i_{A,A'},G_{A,A'}$. The addition of the second and third terms in the exponential generates Feynman diagrams with edges and leaves decorated by $\widehat{K}$ and $\widehat{i}$ instead of *K* and *i*. Additionally, one has a diagram consisting of two source terms σ connected by an edge – this accounts for the exponential prefactor containing $\widehat{\Theta }$ in (155). See Fig. 8.

### Metric dependence of the desynchronized partition function

Our definition of the desynchronized partition function $Z_{A,A'}$ (and its extended version) rely on a Riemannian metric *g* on *M*. In this subsection, we analyze the dependence of *Z* on this metric. Changing the metric induces another deformation of the gauge-fixing operator $d_{A'}^*$. The goal of this subsection is to prove Theorem 4.17 below. Most of the discussion is analogous to the previous subsection and we only sketch the proofs.

#### Remark 4.16

*(Framing anomaly and renormalization)* It is well known that the (synchronized) perturbative Chern–Simons partition function exhibits metric dependence known as *framing anomaly*. Namely, the phase $e^{\frac{i\pi }{4}\psi _A}$ of the synchronized 1-loop part

$$ I_A=e^{\frac{i\pi }{4}\psi _A}\tau ^\frac{1}{2}_A$$

depends on the metric through the eta invariant ψ, as already discussed in Witten’s treatment [Wit89].^53^ The dependence on the metric can be canceled by choosing a framing or 2-framing ϕ of *M*,i.e. a trivialization of *TM* or TM⊕TM^54^, and multiplying $I_{A,A}$ by $e^{\frac{i\dim G}{24}\cdot \frac{S_\textrm{grav}(g,\phi )}{2\pi }}$, where $S_\textrm{grav}(g,\phi ) = S_{CS}(\phi ^*A_{g})$ denotes the evaluation of the Chern–Simons action on Levi-Civita connection $A_g$. For a 2-framing ϕ, one defines $S_\textrm{grav}(g,\phi ) = \frac{1}{2}S_{CS}(A_g \oplus A_g)$. Then, one has that

161 $$\begin{aligned} I_{A}^\textrm{ren}:= e^{\frac{i\dim G}{24}\cdot \frac{S_\textrm{grav}(g,\phi )}{2\pi }}I_{A} \end{aligned}$$

is invariant under variations of the metric. Axelrod and Singer [AS91, AS94] showed that the anomaly persists at even loop orders: boundary strata of the compactified configuration spaces corresponding to the collapse of all vertices of a connected component of a Feynman graph yield potentially non-zero contributions. However, one can show that there exists a power series

162 $$\begin{aligned} c(\hbar ) = \frac{\dim G}{24}\hbar - \frac{h^\vee \dim G}{24\cdot (2\pi )}\hbar ^2 + O(\hbar ^4) \in \hbar {\mathbb {R}}[[\hbar ]] \end{aligned}$$

such that the renormalized perturbative partition function

163 $$\begin{aligned} Z^\textrm{ren}_{A}:=Z_A e^{\frac{i}{\hbar }c(\hbar )\frac{S_\textrm{grav}(g,\phi )}{2\pi }} \end{aligned}$$

is independent of the metric *g*, up to an explicit BV exact term (also for *A* non-smooth), see [CM08, Mne19, Wer19]. Here $h^\vee$ is the dual Coxeter number of ${\mathfrak {g}}$. Comparison with the non-perturbative answer suggests that all higher order terms in (162) vanish, see e.g. the discussion below Eq. (6.124) in [AS91]. In our desynchronized setting, the same anomalies appear, and they can be canceled in the same way.

Let us now consider the effect of an infinitesimal deformation $g \mapsto g + \delta g$. We have

164 $$\begin{aligned} \delta _g d_{A'}^* = [d_{A'}^*,\lambda _{\delta g}] \end{aligned}$$

where $\lambda _{\delta g} \in \Omega ^1(\textrm{Met}, \operatorname {End}(\Omega ^\bullet (M))$ is given by (see Lemma B.7)

165 $$\begin{aligned} \lambda _{\delta g} = \star ^{-1} \delta _g\star = \frac{1}{2} \operatorname {tr}g^{-1}\delta g - \iota _{g^{-1}\delta _g}. \end{aligned}$$

We note that we have

166 $$\begin{aligned} \delta _g\lambda _{\delta g} = - \star ^{-1}(\delta _g\star )\star ^{-1}\delta _g = -\lambda _{\delta _g}^2. \end{aligned}$$

The (*i*, *p*, *K*) triple transforms as

167 $$\begin{aligned} \delta _g i_{A,A'}&= -d_A{\mathbb {I}}_{\delta g}i_{A,A'}, \end{aligned}$$

168 $$\begin{aligned} \delta _g p_{A,A'}&= -p_{A,A'}{\mathbb {P}}_{\delta g} d_A, \end{aligned}$$

169 $$\begin{aligned} \delta _g K_{A,A'}&= [d_A,\Lambda _{\delta g}] + P_{A,A'} {\mathbb {P}}_{\delta g} + {\mathbb {I}}_{\delta g}P_{A,A'} \end{aligned}$$

where

170 $$\begin{aligned} \Lambda _{\delta g} = K_{A,A'}\lambda _{\delta g} K_{A,A'},\qquad {\mathbb {I}}_{\delta g} = K_{A,A'}\lambda _{\delta g}, \qquad {\mathbb {P}}_{\delta g} = \lambda _{\delta g} K_{A,A'} \end{aligned}$$

are the endomorphisms of $\Omega ^\bullet (M,{\mathfrak {g}})$ analogous to $\Lambda ,{\mathbb {I}},{\mathbb {P}}$ defined in (129).

We then have the following theorem:

#### Theorem 4.17

For $A,A' \in \textrm{FC}$ a pair of close flat connections, we have

171 $$\begin{aligned} \delta _g Z_{A,A'}^\textrm{ren}({\textsf{a}}) = -i\hbar \Delta _{\textsf{a}}\left( r_{\delta g}({\textsf{a}})Z^\textrm{ren}_{A,A'}({\textsf{a}})\right) \end{aligned}$$

where $r_{\delta g}({\textsf{a}})$ is given by the sum of connected Feynman diagrams with one edge marked by $\Lambda _{\delta g}$ or one leaf marked by ${\mathbb {I}}_{\delta g}$.

#### Sketch of the proof

This proof is analogous to the proof of Proposition 4.11, using the fact that the renormalization cancels potential contributions from hidden boundary strata.^55^□

#### Extension of *Z* to a horizontal nonhomogeneous form in *g*.

One can perform a construction analogous to the one in Sect. 4.4 and extend the desynchronized partition function to a horizontal non-homogeneous differential form in the metric direction. This generalizes the construction of Axelrod and Singer [AS94] to the desynchronized case and arbitrary kinetic operators.^56^ Namely, one has the following analog of (154):

172 $$\begin{aligned} \begin{aligned} \widehat{i}_{\delta g}&=\sum _{k\ge 0} (K_{A,A'}\lambda _{\delta g})^k i_{A,A'}=i+{\mathbb {I}}_{\delta g}i+\cdots \quad \in \Omega ^{0,0,\bullet }(\mathcal {U}\times \textrm{Met},\textrm{Hom}(H_A^\bullet ,\Omega ^\bullet (M,{\mathfrak {g}}))), \\ \widehat{p}_{\delta g}&= \sum _{k\ge 0} p_{A,A'}(\lambda _{\delta g} K_{A,A'})^k=p+p{\mathbb {P}}_{\delta g}+\cdots \quad \in \Omega ^{0,0,\bullet }(\mathcal {U}\times \textrm{Met},\textrm{Hom}(\Omega ^\bullet (M,{\mathfrak {g}}),H_A^\bullet )), \\ \widehat{K}_{\delta g}&= \sum _{k\ge 0} K_{A,A'}(\lambda _{\delta g} K_{A,A'})^k=K+\Lambda _{\delta g}+\cdots \quad \in \Omega ^{0,0,\bullet }(\mathcal {U}\times \textrm{Met},\textrm{End}(\Omega ^\bullet (M,{\mathfrak {g}}))),\\ \widehat{\Theta }_{\delta g}&=-p_{A,A'}\lambda _{\delta g} K_{A,A'}\lambda _{\delta g} i_{A,A'}\quad \in \Omega ^{0,0,2}(\mathcal {U},\textrm{End}(H^\bullet _A)). \end{aligned} \end{aligned}$$

Here $\textrm{Met}$ denotes the Fréchet manifold of metrics and we are using its exterior calculus. Alternatively, one can restrict to an arbitrary finite-dimensional submanifold thereof. Again, we note that since *K* reduces the form degree along *M*, the sums in (172) are finite: The sum stops at k=2 for $\widehat{i}_{\delta g}$, $\widehat{p}_{\delta g}$ and $\widehat{K}_{\lambda _{\delta g}}$.

##### Remark 4.18

Another way to construct the triple $(\widehat{i}_{\delta g},\widehat{p}_{\delta g},\widehat{K}_{\delta g})$ is to consider the operator^57^

173 $$\begin{aligned} {\widehat{H}}_g = [d_A + \delta _g,d^*_{A',g}] = \widehat{H}_g + (\delta _g d_{A',g}^*) = \widehat{H}_g + [\lambda _{\delta g}, d^*_{A',g}]. \end{aligned}$$

The operator $$H_g + P_{A,A',g} + [\lambda _{\delta g}, d^*_{A',g}]$$ is invertible and its Green’s function can be computed as $$\widehat{G} = G - G[\lambda _{\delta g}, d^*_{A',g}]G + \ldots $$. Upon applying $$d^*_{A',g}$$, one recovers (325). In particular, $$\widehat{K}_{\delta g}$$ coincides, for $A=A^{\prime }$ an acyclic flat connection, with the extended propagator of Axelrod and Singer [AS94, Section 4].

Similarly to Lemma 4.14 we have:

##### Lemma 4.19

The triple $(\widehat{i}_{\delta g},\widehat{p}_{\delta g},\widehat{K}_{\delta g})$ satisfies the following relations:

174a $$\begin{aligned} (\delta _{g}+[d_A,-])\widehat{K}_{\delta g}= &  1-\widehat{i}_{\delta g}\,\widehat{p}_{\delta g}, \end{aligned}$$

174b $$\begin{aligned} (\delta _{g}+d_A)\widehat{i}_{\delta g}= &  \widehat{i}_{\delta g}\, \widehat{\Theta }_{\delta g}, \end{aligned}$$

174c $$\begin{aligned} \delta _{g} \widehat{p}_{\delta g} - \widehat{p}_{\delta g} \, d_A= &  -\widehat{\Theta }_{\delta g}\, \widehat{p}_{\delta g}, \end{aligned}$$

174d $$\begin{aligned} \widehat{K}_{\delta g}\, \widehat{i}_{\delta g}= &  0, \end{aligned}$$

174e $$\begin{aligned} \widehat{p}_{\delta g}\, \widehat{K}_{\delta g}= &  0, \end{aligned}$$

174f $$\begin{aligned} \widehat{K}_{\delta g}^2= &  0, \end{aligned}$$

174g $$\begin{aligned} \widehat{p}_{\delta g} \,\widehat{i}_{\delta g}= &  1. \end{aligned}$$

This is a special case of Proposition C.1 for $\mathbb{G}\mathbb{F}=\textrm{Met}$. The extended partition function

175 $$\begin{aligned} &  \widehat{Z}_{A,A';\delta g}({\textsf{a}}) =e^{\frac{i}{\hbar }S_{CS}(A)} \nonumber \\ &  \quad e^{\frac{\pi i}{4}\psi _A}\tau _{A}^{1/2} e^{\frac{i}{\hbar }\,\frac{1}{2}\langle {\textsf{a}}, \widehat{\Theta }({\textsf{a}})\rangle }\;\exp \sum _\Gamma \frac{(-i\hbar )^{l(\Gamma )-1}}{|\textrm{Aut}(\Gamma )|}\widehat{\Phi }_{\Gamma ,A,A';\delta g}({\textsf{a}}) \nonumber \\ &  \quad \in \Omega ^{0,0,\bullet }(\mathcal {U}\times \textrm{Met},\textrm{Dens}^{\frac{1}{2},\textrm{formal}}(H_A [1])) \end{aligned}$$

then satisfies the differential Master Equation

176 $$\begin{aligned} (\delta _g - i\hbar \Delta _{\textsf{a}})\widehat{Z}_{A,A';\delta g}^\textrm{ren} = 0, \end{aligned}$$

which one can prove analogously to Theorem 4.13; the superscript “ren” means that we include the renormalization factor as in (163). Again, one can view $\widehat{Z}_{A,A';\delta g}$ as the perturbative expansion of the path integral

177 $$\begin{aligned} &  \widehat{Z}_{A,A';\delta g}({\textsf{a}})=\int _{\mathcal {L}=\Omega _{d^*_{A'}\mathrm {-ex}}[1]}\mathcal {D}\alpha _\textrm{fl} \; \exp \frac{i}{\hbar }\Big ( S_{CS}(A+i({\textsf{a}})+\alpha _\textrm{fl}) \nonumber \\ &  \quad -\int _M\frac{1}{2} \big \langle \alpha _\textrm{fl}, \lambda _{\delta g}\alpha _\textrm{fl} \big \rangle +\big \langle \alpha _\textrm{fl},\lambda _{\delta g} i({\textsf{a}}) \big \rangle \Big ) \end{aligned}$$

with the last two terms generating additional vertices that sum up to $K_{\delta g}, i_{\delta g}$ and $\Theta _{\delta g}$ respectively.

### Partition function extended to a nonhomogeneous form on $\textrm{FC}\times \textrm{FC}\times \textrm{Met}$.

#### Connection $\nabla ^\textrm{Hodge}$

In this section, $$\textrm{Met}$$ will denote the space of Riemannian metrics on *M* and $$\underline{\mathcal {U}}=\{(A,A',g)\}$$ will stand for a sufficiently thin open neighborhood of $$\textrm{Diag}\times \textrm{Met}$$ in $$\textrm{FC}'\times \textrm{FC}'\times \textrm{Met}$$, where each fixed-*g* slice consists of pairs $\left(A,A^{\prime }\right)$ that are close w.r.t. *g*.

Let

178 $$\begin{aligned} H=H_{\delta A}+H_{\delta A'}+H_{\delta g}\quad \in \Omega ^1(\underline{\mathcal {U}},\textrm{End}(\Omega ^\bullet (M,{\mathfrak {g}}))), \end{aligned}$$

where

179a $$\begin{aligned} H_{\delta A}= &  -\Big (K\textrm{ad}_{\delta A} dK+K\textrm{ad}_{\delta A} P + P\textrm{ad}_{\delta A} K\Big ), \end{aligned}$$

179b $$\begin{aligned} H_{\delta A'}= &  -\Big (dG\textrm{ad}^*_{\delta A'}Kd + dG\textrm{ad}^*_{\delta A'}P+P\textrm{ad}^*_{\delta A'}Gd \Big ), \end{aligned}$$

179c $$\begin{aligned} H_{\delta g}= &  dK\lambda _{\delta g} Kd+ dK \lambda _{\delta g} P+ P\lambda _{\delta g} Kd. \end{aligned}$$

We are suppressing the subscripts in $d_A, d^*_{A'}, P_{A,A'}, K_{A,A'}$. Furthermore, let

180 $$\begin{aligned} \Psi =\int _M \frac{1}{2}\langle B, H(B) \rangle \quad \in \Omega ^1(\underline{\mathcal {U}})\otimes \textrm{Sym}^2(\Omega ^\bullet (M,{\mathfrak {g}})[1])^*. \end{aligned}$$

Here $B\in \Omega ^\bullet (M,{\mathfrak {g}})[1]$ is the field. Also, we consider the connection

181 $$\begin{aligned} \nabla ^\textrm{Hodge}= \delta ^\textrm{tot}+ H = \delta ^\textrm{tot} + \{\Psi ,-\}_B \end{aligned}$$

on the trivial bundle ${\underline{\Omega }}$ over $\underline{\mathcal {U}}$ with fiber $\Omega ^\bullet (M,{\mathfrak {g}})[1]$. Here $\{,\}_B$ stands for the Poisson bracket in the fiber and $\delta ^\textrm{tot}=\delta _A+\delta _{A'}+\delta _g$ is the de Rham differential on $\underline{\mathcal {U}}$.

We have the following.

##### Lemma 4.20

- The connection $\nabla ^\textrm{Hodge}$ preserves the harmonic, exact and coexact subbundles in ${\underline{\Omega }}$.^58^
- ∇ is symplectic, i.e., its parallel transport along any path in the base is a symplectomorphism between fibers w.r.t. the BV symplectic form $\omega =\int _M \langle - {\mathop {,}\limits ^{\wedge }} -\rangle$.
- The curvature of $\nabla ^\textrm{Hodge}$ is
  182 $$\begin{aligned}&F_{\nabla ^\textrm{Hodge}}=P\textrm{ad}_{\delta A}(K\lambda _{\delta g}-G\textrm{ad}^*_{\delta A'})P+ P(\lambda _{\delta g} K-\textrm{ad}^*_{\delta A'}G)\textrm{ad}_{\delta A} P+ \nonumber \\&\quad +d(K\lambda _{\delta g}-G\textrm{ad}^*_{\delta A'})(Kd+P)\textrm{ad}_{\delta A}K+K\textrm{ad}_{\delta A}(dK+P)(\lambda _{\delta g} K-\textrm{ad}^*_{\delta A'}G)d. \end{aligned}$$
  In particular, the curvature has vanishing $(\delta A)^2$, $(\delta A')^2$, $(\delta g)^2$ and $\delta A' \delta g$ terms; only $\delta A \delta A'$ and $\delta A \delta g$ terms are nonzero.
- For a fixed metric *g* and restricted to harmonic forms, $\nabla ^\textrm{Hodge}$ coincides with $\nabla ^\textrm{Harm}$ of Sect. 2.5.3.

(Proven by an explicit computation.)

##### Remark 4.21

59$\nabla ^\textrm{Hodge}$ can be seen as a sum of three “shift-and-project connections” (cf. Remark 2.37) induced on the harmonic, exact and coexact subbundles in ${\underline{\Omega }}$ from the trivial connection on ${\underline{\Omega }}$. Put another way, one can write

183 $$\begin{aligned} \nabla ^\textrm{Hodge}=\delta ^\textrm{tot}-\delta ^\textrm{tot} (P)\, P- \delta ^\textrm{tot}(dK)\, dK - \delta ^\textrm{tot}(Kd)\, Kd. \end{aligned}$$

Here *P*, *dK*, *Kd* are the fiberwise projections onto the three terms in the Hodge decomposition.

By restricting $\nabla ^\textrm{Hodge}$ to harmonic forms and projecting to cohomology $H_A$, $\nabla ^\textrm{Hodge}$ induces the connection $\nabla ^{\mathbb {H}}$ on the cohomology bundle bundle ${\mathbb {H}}$ over $\underline{\mathcal {U}}$ with fiber $H_A$.^60^ Notice that the cohomology bundle is trivial along $\textrm{GF} = \textrm{FC}_{A'} \times \textrm{Met}$ directions and the connection is also trivial in these directions, i.e.

184 $$\begin{aligned} \nabla ^{\mathbb {H}}= \nabla ^{\mathbb {H}}_{A} + \delta _{\textrm{GF}}. \end{aligned}$$

The curvature of $\nabla ^{\mathbb {H}}$ corresponds to the harmonic-harmonic block of (182):

185 $$\begin{aligned} F_{\nabla ^{\mathbb {H}}}=p\Big ((\textrm{ad}_{\delta A}(K\lambda _{\delta g}-G\textrm{ad}^*_{\delta A'}))+ (\lambda _{\delta g} K-\textrm{ad}^*_{\delta A'}G)\textrm{ad}_{\delta A}\Big )i. \end{aligned}$$

Furthermore, we will denote by $\nabla ^\mathcal {D}$ the connection induced by $\nabla ^{\mathbb {H}}$ on the bundle $\mathcal {D}$ of formal half-densities on $H_A{ [1]}$ over $\underline{\mathcal {U}}$.

#### Extended partition function

Denote^61^

$$\begin{aligned} \Psi ^G= \int _M\langle \delta A,B \rangle . \end{aligned}$$

Let

186 $$\begin{aligned} {\check{S}}(B)=S_{CS}(A+B)-\Psi ^G - \Psi \quad \in \Omega ^{\bullet }(\underline{\mathcal {U}})\otimes \textrm{Sym}(\Omega ^\bullet (M,{\mathfrak {g}})[1])^*. \end{aligned}$$

– a form on $\underline{\mathcal {U}}$ valued in polynomials in *B*. We split the field as $B=i_{A,A'}({\textsf{a}})+\alpha _\textrm{fl}$ and consider the perturbative path integral

187 $$\begin{aligned} {\check{Z}}({\textsf{a}})=\int _{\textrm{im}(d^*_{A'})} \mathcal {D}\alpha _\textrm{fl} \; e^{\frac{i}{\hbar } {\check{S}}(i_{A,A'}({\textsf{a}})+\alpha _\textrm{fl})}\quad \in \Omega ^\bullet (\underline{\mathcal {U}}, \textrm{Dens}^{\frac{1}{2},\textrm{formal}}(H_A[1]) ). \end{aligned}$$

Perturbative evaluation of (187) yields the following:

188 $$\begin{aligned}&{\check{Z}}({\textsf{a}})=e^{\frac{i}{\hbar }S_{CS}(A)} e^{\frac{\pi i}{4}\psi _A}\tau _A^{1/2} e^{\frac{i}{\hbar } (-\langle [\delta A],{\textsf{a}}\rangle +\frac{1}{2}\langle {\textsf{a}}, {\check{\Theta }}({\textsf{a}})\rangle )}\cdot \nonumber \\&\quad \cdot \exp \sum _\Gamma \frac{(-i\hbar )^{l(\Gamma )-1}}{|\textrm{Aut}(\Gamma )|}{\check{\Phi }}_\Gamma . \end{aligned}$$

Here:

- Γ runs over connected trivalent graphs with leaves, as usual.
- ${\check{\Phi }}_\Gamma$ is the Feynman weight of the graph Γ, where an edge is assigned the extended propagator
  189 $$\begin{aligned} {\check{K}}=\sum _{k=0}^2 K(HK)^k \end{aligned}$$
  – a nonhomogeneous form on $\underline{\mathcal {U}}$ valued in $\textrm{End}(\Omega ^\bullet (M,{\mathfrak {g}}))$; here *H* is the 1-form (178) of the connection $\nabla ^\textrm{Hodge}$. A leaf is assigned the expression
  190 $$\begin{aligned} {\check{i}}({\textsf{a}})=\sum _{k= 0}^2 (KH)^k i({\textsf{a}})+K\delta A. \end{aligned}$$
  Note that ${\check{i}}({\textsf{a}})$ is affine-linear in ${\textsf{a}}$, rather than just linear. Both (189) and (190) stop at k=2 because *K* decreases form degree by 1.
- $${\check{\Theta }}({\textsf{a}})$$ stands for
  191 $$\begin{aligned} {\check{\Theta }}({\textsf{a}})= -p H K H i({\textsf{a}}). \end{aligned}$$
  It is a 2-form on $\underline{\mathcal {U}}$ with values in endomorphisms of $H_A[1]$.

##### Remark 4.22

**Fig. 9:**
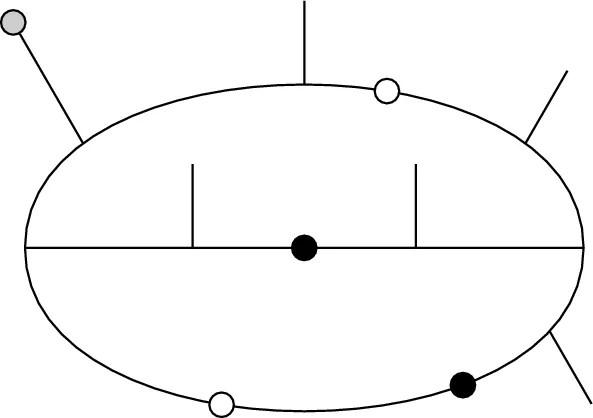
This graph evaluates to an element of $$\Omega ^{1,2,2}(\underline{\mathcal {U}},\textrm{Dens}^{\frac{1}{2},\textrm{formal}}(H_A[1])$$ (notice gray/white/black vertices carry form degree 1 along $A/A^{\prime }/g$). The ghost number of this graph is -5

**Fig. 10:**
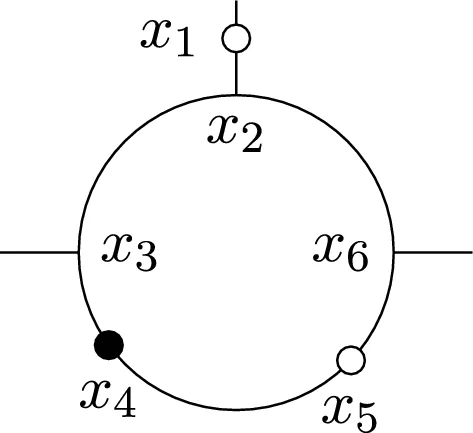
This diagram evaluates to $\operatorname {Str} \textrm{ad}_{G\textrm{ad}^*_{\delta A'}i({\textsf{a}})}K \textrm{ad}_{i({\textsf{a}})}K\lambda _{\delta _g}G\textrm{ad}^*_{\delta A}K \textrm{ad}_{i({\textsf{a}})}K \in \Omega ^{0,2,1}(\underline{\mathcal {U}},\textrm{Dens}^{\frac{1}{2},\textrm{formal}}(H_A[1]))$ (it can be written as a supertrace because it is a 1-loop graph)

To elucidate the relationship between ${\check{Z}}$ and *Z*, one can express (188) in terms of the regular Feynman rules, where an edge is assigned *K* and a leaf is assigned $i({\textsf{a}})$, by adding extra vertices carrying the form degree along $\underline{\mathcal {U}}$: bivalent black and white vertices and gray univalent vertices. White vertices are assigned $H_{\delta A'}$, black vertices are assigned $H_{\delta g}$, gray vertices are assigned δA. In addition, edges decorated by more than two bivalent vertices vanish automatically. See Fig. 9. When sandwiched between *K* and *K* (or *K* and *i*) only a single term in $H_{\delta A'}$ and $H_{\delta g}$ survives. Therefore, one can evaluate black vertices with $\lambda _{\delta g}$ and white vertices to $dG\textrm{ad}^*_{\delta A'}$. The latter effectively acts by applying “$(d^*)^{-1}$” on one of the internal edges incident to the vertex. See for instance the diagram in Fig. 10.

#### Differential quantum master equation

##### Theorem 4.23

The following differential quantum master equation holds:

192 $$\begin{aligned} \Big (\nabla ^\mathcal {D} -i\hbar \Delta _{\textsf{a}}-\frac{i}{\hbar } \frac{1}{2} \langle {\textsf{a}}, F_{\nabla ^{\mathbb {H}}}{\textsf{a}}\rangle \Big ) (e^{\frac{i}{\hbar } c(\hbar )\frac{S_\textrm{grav}(g,\phi )}{2\pi }} {\check{Z}}) =0, \end{aligned}$$

with $F_{\nabla ^{\mathbb {H}}}$ as in (185).

**Heuristic Path Integral Argument:** Denote $\mathcal {L}=\textrm{im}(d^*_{A'})$ the gauge fixing Lagrangian. We have

193 $$\begin{aligned}&\nabla ^\mathcal {D} \int _{\mathcal {L}} e^{\frac{i}{\hbar } {\check{S}}(B)}\nonumber \\&\quad = \int _{\mathcal {L}} e^{\frac{i}{\hbar } {\check{S}}(B)} \frac{i}{\hbar }(\delta _A S_{CS}(A+B)-\delta ^\textrm{tot}\Psi +\{{\check{S}},\Psi \}_B-i\hbar \Delta _B\Psi ). \end{aligned}$$

Here in the brackets in the r.h.s., the first two terms account for $$\delta ^\textrm{tot}$$ acting on the integrand and the last two terms account for the change of gauge-fixing induced by an infinitesimal change of $A,A^{\prime },g$, cf. [CM08, Proposition 2]. Also, note that we can write the first term in the r.h.s. of (193) as

$$\begin{aligned} \delta _A S_{CS}(A+B)=\{S_{CS}(A+B), \Psi ^G \}_B. \end{aligned}$$

Next, applying the BV Laplacian in zero-modes to ${\check{Z}}$, we have, using the BV-Stokes’ theorem,

194 $$\begin{aligned}&-i\hbar \Delta _{\textsf{a}}\int _{\mathcal {L}} e^{\frac{i}{\hbar } {\check{S}}(B)}= \int _{\mathcal {L}} -i\hbar \Delta _B e^{\frac{i}{\hbar } {\check{S}}(B)} =\nonumber \\&\quad = \int _{\mathcal {L}}e^{\frac{i}{\hbar } {\check{S}}(B)} \frac{i}{\hbar }(\frac{1}{2}\{{\check{S}},{\check{S}}\}_B-i\hbar \Delta _B {\check{S}}) \nonumber \\&\quad =\int _{\mathcal {L}}e^{\frac{i}{\hbar } {\check{S}}(B)} \frac{i}{\hbar }( -\{S_{CS},\Psi ^G\}_B-\{{\check{S}},\Psi \}_B-\frac{1}{2} \{\Psi ,\Psi \}_B+i\hbar \Delta _B \Psi )\nonumber \\&\quad =\int _{\mathcal {L}}e^{\frac{i}{\hbar } {\check{S}}(B)} \frac{i}{\hbar }( -\delta _AS_{CS}-\{{\check{S}},\Psi \}_B+\delta ^\textrm{tot}\Psi -\Psi ^F+i\hbar \Delta _B \Psi ). \end{aligned}$$

In the last transition we used that $\delta ^\textrm{tot}\Psi +\frac{1}{2}\{\Psi ,\Psi \}_B=\Psi ^F$, with $\Psi ^F=\frac{1}{2} \langle B,F_{\nabla ^\textrm{Hodge}} B\rangle$—the quadratic form associated with the curvature (182) of $\nabla ^\textrm{Hodge}$. Note that $\Psi ^F|_{B=i({\textsf{a}})+\alpha _\textrm{fl}}=\frac{1}{2} \langle {\textsf{a}},F_{\nabla ^{\mathbb {H}}} {\textsf{a}}\rangle$ for $\alpha _\textrm{fl}\in \mathcal {L}$.

Thus, (193) and (194) differ by ${\check{Z}}\frac{i}{\hbar }\frac{1}{2} \langle {\textsf{a}},F_{\nabla ^{\mathbb {H}}} {\textsf{a}}\rangle$.

##### Sketch of diagrammatic proof of Theorem 4.23

For the purpose of the proof we expand the partition function ${\check{Z}}$ as a sum over graphs with trivalent and univalent vertices and leaves. Edges are assigned the extended propagator ${\check{K}}= \sum _{k=0}^2 K(HK)^k$, leaves the extended inclusion ${\check{i}}_{\textrm{GF}}({\textsf{a}}) = \sum _{k=0}^2 (KH)^ki({\textsf{a}})$, and univalent vertices are assigned δA. Notice that we have

195 $$\begin{aligned} \delta _A {\check{K}}&= {\check{K}}\textrm{ad}_{\delta A}{\check{K}}, \end{aligned}$$

196 $$\begin{aligned} \nabla ^{\mathbb {H}}_A {\check{i}}_{GF}&= {\check{K}}\textrm{ad}_{\delta A}{\check{i}}_\textrm{GF}, \end{aligned}$$

197 $$\begin{aligned} \delta _{\textrm{GF}}{\check{K}}&= - [d,{\check{K}}] + \textrm{id} - {\check{i}}_{\textrm{GF}}{\check{p}}_\textrm{GF}, \end{aligned}$$

198 $$\begin{aligned} \delta _\textrm{GF}{\check{i}}_\textrm{GF}&= - d{\check{i}}_\textrm{GF} + {\check{i}}_{\textrm{GF}}\check{\Theta }. \end{aligned}$$

**Fig. 11:**
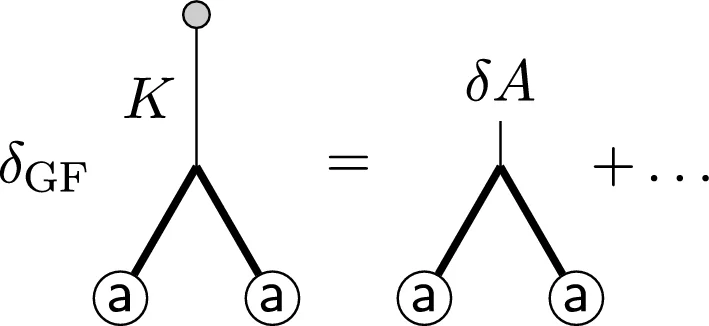
The graph on the right evaluates to $\frac{1}{2}\langle {\textsf{a}}, F_\nabla ({\textsf{a}})\rangle + \nabla ^{\mathbb {H}}_A\check{\Theta }({\textsf{a}})$, with $F_\nabla$ given in (185). Thick edge stands for ${\check{i}}$, thin edges stand for *K* (between vertices) or *i* (at leaves)

Now the proof follows closely the proof of Theorem 4.13. When computing $\delta _{\textrm{GF}}{\check{Z}}$, there are now additional terms when $\delta _\textrm{GF}$ hits the edge incident to a univalent vertex. Here, the terms $\textrm{id} - {\check{i}}_{\textrm{GF}}{\check{p}}_\textrm{GF}$ survive. The first term is canceled by a graph with a δA leaf that is produced when applying $\nabla ^{\mathbb {H}}_A$. A special case occurs for the graph consisting of a graph with one univalent vertex, one trivalent vertex and two leaves, here one such term survives and is canceled by the curvature (185) and $\nabla ^{\mathbb {H}}_A\check{\Theta }$, see Fig. 11. The second term involving ${\check{i}}_\textrm{GF}{\check{p}}_\textrm{GF}$ (which applied to δA is simply *P*) is canceled by a term in $\Delta _{\textsf{a}}(e^{-\frac{i}{\hbar }\langle [\delta A], {\textsf{a}}\rangle }\Phi _\Gamma ({\textsf{a}}))$.

As always, there are extra terms in the metric dependence of the partition function due to the total collapse of a connected component of a Feynman graph (canceled by $S_\textrm{grav}$ counterterm). Incorporating that, we get (192). □

##### Remark 4.24

Note that while $\nabla ^\mathcal {D}$ is not flat, the superconnection

199 $$\begin{aligned} {\check{\nabla }}=\nabla ^\mathcal {D}-i\hbar \Delta _{\textsf{a}}-\frac{i}{\hbar } \frac{1}{2} \langle {\textsf{a}},F_{\nabla ^{\mathbb {H}}}{\textsf{a}}\rangle \end{aligned}$$

on the bundle of formal half-densities on $H_A$, appearing in (192), is flat. In more detail: one has

200 $$\begin{aligned} (\nabla ^\mathcal {D})^2=\langle F_{\nabla ^{\mathbb {H}}}({\textsf{a}}),\frac{\partial }{\partial {\textsf{a}}} \rangle +\frac{1}{2} \textrm{Str}_{H_A[1]} F_{\nabla ^{\mathbb {H}}} \end{aligned}$$

and

201 $$\begin{aligned} &  {\check{\nabla }}^2= (\nabla ^\mathcal {D})^2 + \underbrace{[\nabla ^\mathcal {D},-i\hbar \Delta _{\textsf{a}}]}_0 - \frac{i}{\hbar } \frac{1}{2}\underbrace{[\nabla ^\mathcal {D}, \langle {\textsf{a}},F_{\nabla ^{\mathbb {H}}}{\textsf{a}}\rangle ]}_{=\langle {\textsf{a}}, (\nabla ^{\mathbb {H}}F_{\nabla ^{\mathbb {H}}}){\textsf{a}}\rangle =0 \,\mathrm {by\,Bianchi\, identity}}+ \nonumber \\ &  \quad + \underbrace{(-i\hbar \Delta _{\textsf{a}})^2}_0-[\Delta _{\textsf{a}},\frac{1}{2} \langle {\textsf{a}},F_{\nabla ^{\mathbb {H}}}{\textsf{a}}\rangle ]+\underbrace{(-\frac{i}{\hbar } \frac{1}{2} \langle {\textsf{a}},F_{\nabla ^{\mathbb {H}}}{\textsf{a}}\rangle )^2}_0 \nonumber \\ &  \quad = (\nabla ^\mathcal {D})^2+\Big \{\frac{1}{2} \langle {\textsf{a}},F_{\nabla ^{\mathbb {H}}}{\textsf{a}}\rangle ,-\Big \}_{\textsf{a}}-\Delta _{\textsf{a}}\Big (\frac{1}{2} \langle {\textsf{a}},F_{\nabla ^{\mathbb {H}}}{\textsf{a}}\rangle \Big )\underset{(200)}{=}0. \end{aligned}$$

#### Partial extensions of *Z* along *A*, $A^{\prime }$ and *g*—comparison with previous results

**Varying** $A^{\prime }$. If we consider the slice of $$\underline{\mathcal {U}}$$ with fixed *A*, *g* and varying $A^{\prime }$, the path integral (187) reduces to (160). The corresponding restriction of (192) to $$\delta A=\delta g=0$$ is equivalent to (156). Also, in this case $${\check{K}},{\check{i}},{\check{\Theta }}$$ reduce to $$\widehat{K},\widehat{i}$$ and $$\widehat{\Theta }$$ of Sect. 4.4.

**Varying** *g*. Similarly, if we consider the slice of $$\underline{\mathcal {U}}$$ with fixed $A,A^{\prime }$ and varying *g*, $${\check{Z}}$$ reduces to (177), (175); dQME (192) becomes (176); $${\check{K}},{\check{i}},{\check{\Theta }}$$ become $$\widehat{K}_{\delta g},\widehat{i}_{\delta g}$$ and $$\widehat{\Theta }_{\delta g}$$ of Sect. 4.5.1, respectively.

**Varying** *A*. One can also consider varying *A* while keeping $A^{\prime },g$ fixed. In this case (188) simplifies to

202 $$\begin{aligned} &  {\check{Z}}_{\delta A}=e^{\frac{i}{\hbar } S_{CS}(A)} e^{\frac{\pi i}{4}\psi _A}\tau _A^{1/2} e^{-\frac{i}{\hbar }\langle [\delta A],{\textsf{a}}\rangle } \nonumber \\ &  \quad \exp \sum _\Gamma \frac{(-i\hbar )^{l(\Gamma )-1}}{|\textrm{Aut}(\Gamma )|} {\check{\Phi }}_\Gamma \end{aligned}$$

where in ${\check{\Phi }}_\Gamma$, edges are decorated with the usual non-extended propagator *K* and leaves are decorated with $i({\textsf{a}})-K\delta A$. By (192), ${\check{Z}}_{\delta A}$ satisfies the dQME in the direction of shifts of *A*:

203 $$\begin{aligned} (\nabla ^{{\mathbb {H}},A'}-i\hbar \Delta _{\textsf{a}}){\check{Z}}_{\delta A}=0 \end{aligned}$$

which implies

204 $$\begin{aligned} (\nabla ^{{\mathbb {H}},A'}-\langle [\delta A],\frac{\partial }{\partial {\textsf{a}}} \rangle +\langle p\, \textrm{ad}_{K\delta A} i({\textsf{a}}),\frac{\partial }{\partial {\textsf{a}}}\rangle + O((\delta A)^2) -i\hbar \Delta _{\textsf{a}})\widetilde{Z}_{\delta A}=0 \end{aligned}$$

where $\widetilde{Z}_{\delta A}$ is (202) with two modifications: (i) factor $e^{-\frac{i}{\hbar }\langle [\delta A],{\textsf{a}}\rangle }$ is removed, (ii) the graph consisting of the single cubic vertex is removed from the sum over Γ.

We note that the result (204) restricted to degree 1 in δA is equivalent to (150) restricted to $\delta A^{\prime }=0$ as one has, from inspection of Feynman diagrams,

205 $$\begin{aligned} \widetilde{Z}_{\delta A}=Z+r_{\widetilde{\delta A'}=-d_{A'}K\delta A}Z+\underbrace{\cdots }_{\textrm{degree}\;\ge 2\;\textrm{in}\;\delta A}, \end{aligned}$$

with *r* and $\widetilde{\delta A'}$ as in (150), (151).^62^ To elucidate this equivalence, we note for harmonic shifts δA, the first two terms in (204) yield the connection $\widetilde{\nabla }^G$ (124); for δA exact, there is a discrepancy between $\nabla ^{{\mathbb {H}},A'}$ and $\nabla ^\textrm{tot}$ (149) compensated by the third term in (204).^63^

## The Global Partition Function

In this section we will discuss the properties of the “synchronized” partition function $Z(A_0,{\textsf{a}}) = Z_{A_0,A_0}({\textsf{a}})$ defined in Definition 3.1 seen as a family ${\underline{Z}}$ over the moduli space ${\mathcal {M}}'$. We introduce the *global partition function* $Z^\textrm{glob}$ – a volume form on the moduli space ${\mathcal {M}}'$ arising from modifying ${\underline{Z}}$ to a global ($\nabla ^G$-horizontal) object by a BV-exact term and then restricting it to ${\textsf{a}}=0$. Finally, we study the dependence of $Z^\textrm{glob}$ on metric.

### Perturbative partition function ${\underline{Z}}$ on the moduli space

#### Bundles over $\textrm{FC}'$ and ${\mathcal {M}}'$.

We recall that $\textrm{FC}$ denotes the space of all flat connections, ${\mathcal {G}} = C^\infty (M,G)$ the gauge group and

206 $$\begin{aligned} {\mathcal {M}}= \textrm{FC}/{\mathcal {G}} \end{aligned}$$

the moduli space of flat connections, with $\pi :\textrm{FC}\rightarrow {\mathcal {M}}$ the projection. Consider the subsets

207 $$\begin{aligned} \textrm{FC}'\subset \textrm{FC}, \qquad {\mathcal {M}}' \subset {\mathcal {M}} \end{aligned}$$

of smooth irreducible points, by the results of Sect. 2, they are smooth manifolds. Over $\textrm{FC}'$, we have the cohomology bundle ${\mathbb {H}} \rightarrow \textrm{FC}'$, with fiber over $A_0$ given by $H^\bullet _{A_0}$. The gauge group acts on ${\mathbb {H}}$ by conjugation, we have ${}^\textrm{g}(H^\bullet _{A_0}) = H_{{}^\textrm{g}A_0}$. The quotient ${\mathbb {H}}[1]/{\mathcal {G}} \rightarrow {\mathcal {M}}'$ is isomorphic to the bundle $T{\mathcal {M}}' \oplus T^*[-1]{\mathcal {M}}'$.

#### Perturbative partition function

Restricted to $\textrm{FC}'$, the perturbative partition function *Z* defined in (98) defines a section

208 $$\begin{aligned} Z \in e^{\frac{i}{\hbar }S_{CS}(-)} \Gamma (\textrm{FC}', \widehat{\operatorname {Sym}}\,{\mathbb {H}}[1]^* \otimes \operatorname {Det}^\frac{1}{2} {\mathbb {H}} )[[\hbar ]]. \end{aligned}$$

From [CM08] we have the following result.

##### Lemma 5.1

Suppose $A_0$ is an irreducible flat connection. Then

$$\begin{aligned} Z_{A_0}({\textsf{a}}) \in \widehat{\operatorname {Sym}}\,H^1_{A_0} \otimes \operatorname {Det}^\frac{1}{2}(H^\bullet _{A_0})[[\hbar ]] \end{aligned}$$

depends only on the 1-form part ${\textsf{a}}^{1}$ of ${\textsf{a}}$.

Finally, we have the following:

##### Proposition 5.2

The Chern–Simons partition function *Z* is equivariant with respect to the action of the gauge group on $\textrm{FC}'$ and ${\mathbb {H}}$.

##### Proof

Follows immediately from Proposition 4.1 by restricting to the diagonal $A=A^{\prime }$. □

##### Corollary 5.3

The perturbative partition function defines a section

209 $$\begin{aligned} {\underline{Z}} \in e^{\frac{i}{\hbar }S_{CS}(-)} \Gamma ({\mathcal {M}}', \widehat{\operatorname {Sym}}\,T^*{\mathcal {M}}' \otimes \operatorname {Det}^\frac{1}{2}(T{\mathcal {M}}'\oplus T^*[-1]{\mathcal {M}}')^*)[[\hbar ]]. \end{aligned}$$

##### Proof

This follows from (208) together with Lemma 5.1 and Proposition 5.2 (notice that the quotient bundle ${\mathbb {H}}^1[1]/{\mathcal {G}} \cong T{\mathcal {M}}'$.) □

#### Naive global partition function

Restricting $Z, {\underline{Z}}$ to ${\textsf{a}}=0$, we obtain the naive global partition functions

210 $$\begin{aligned} \begin{aligned} Z^\textrm{glob,naive}_A&= Z_A(0) \in e^{\frac{i}{\hbar }S_{CS}(A)}\Gamma (\textrm{FC}', \operatorname {Det}^\frac{1}{2}{\mathbb {H}} )[[\hbar ]], \\ {\underline{Z}}^\textrm{glob,naive}_{A}&= {\underline{Z}}_{A}(0) \in e^{\frac{i}{\hbar }S_{CS}(A)}\Gamma ({\mathcal {M}}', \operatorname {Det}^\frac{1}{2}(T{\mathcal {M}}'\oplus T^*[-1]{\mathcal {M}}')^*)[[\hbar ]] . \end{aligned} \end{aligned}$$

##### Remark 5.4

The bundle $\operatorname {Det}^\frac{1}{2}(T{\mathcal {M}}'\oplus T^*[-1]{\mathcal {M}}')^*$ is canonically isomorphic to the bundle of top forms $\operatorname {Det}(T^*{\mathcal {M}}')$ over ${\mathcal {M}}'$. From the BV viewpoint, it is also natural to identify this bundle with the subbundle of half-densities on $T^*[-1]{\mathcal {M}}'$ which do not depend on the fiber coordinates.

In Sect. 5.4 below we will construct the non-naive global partition function—a modification of ${\underline{Z}}^\textrm{glob,naive}$ yielding an invariant of *M* as a framed 3-manifold (Theorem 5.17).

### A formula for the Grothendieck connection

First we introduce some notations that we will be using in the remainder of this section.

Let Ξ be the one-form component (as a form on $\underline{\mathcal {U}}$) of the tree part of the extended partition function ${\check{Z}}$ (188):

211 $$\begin{aligned} \Xi = -\langle [\delta A],{\textsf{a}}\rangle +\sum _T \frac{1}{|\textrm{Aut}(T)|}\check{\Phi }_{T}({\textsf{a}}) \Big |_{\Omega ^1(\underline{\mathcal {U}})}\quad \in \Omega ^1(\underline{\mathcal {U}},\widehat{\textrm{Sym}} (H_A[1])^*) \end{aligned}$$

with *T* running over trivalent trees, with notations as in (188). Ξ splits as a 1-form along *A* plus a 1-form along $A^{\prime }$ plus a 1-form along *g*:

212 $$\begin{aligned} \Xi =\Xi _{\delta A}+\Xi _{\delta A'}+\Xi _{\delta g}. \end{aligned}$$

Furthermore, let

213 $$\begin{aligned} I^*:\Omega ^\bullet (\mathcal {U})\rightarrow \Gamma (\textrm{FC}',\wedge ^\bullet (H^1_A)^*) \end{aligned}$$

be the map which restricts a form on $$\mathcal {U}\subset \textrm{FC}'\times \textrm{FC}'$$ to the diagonal $A^{\prime }=A$ and evaluates it on (*A*, *A*)-*harmonic* tangent vectors $$\delta A'=\delta A$$.

Then, given a form ω on $$\mathcal {U}$$ valued in the bundle $$\mathcal {D}=\textrm{Dens}^{\frac{1}{2},\textrm{formal}}(H_A[1])$$ and invariant under gauge transformations, we can construct a $$\mathcal {D}$$-valued form on $${\mathcal {M}}'$$ by evaluating ω on the diagonal $A^{\prime }=A$ and harmonic tangent vectors $$\delta A'=\delta A$$, and passing to gauge-equivalence classes. I.e., we have a map

214 $$\begin{aligned} \pi _*I^*:\Omega ^\bullet (\mathcal {U},\mathcal {D})^\textrm{Gauge}\rightarrow \Omega ^\bullet ({\mathcal {M}}',\mathcal {D}). \end{aligned}$$

Here $\pi :\textrm{FC}\rightarrow {\mathcal {M}}$ is the projection to gauge-equivalence classes. The superscipt “Gauge” means “invariants under the action of the gauge group.”

Denote215—it is the term $-\langle [\delta A],{\textsf{a}}\rangle$ plus the sum of trees with one white binary vertex $H_{\delta A'=i[\delta A]}$, in terms of Remark 4.22.

#### Proposition 5.5

Grothendieck connection associated to the formal exponential map $\underline{\varphi }$ on ${\mathcal {M}}'$, seen as an operator on $\Omega ^\bullet ({\mathcal {M}}',\mathcal {D})$, can be written as

216 $$\begin{aligned} \nabla ^G=\nabla ^{{\mathbb {H}}}_{\mathcal {M}}+\{\xi ,-\}+\Delta _{\textsf{a}}\xi . \end{aligned}$$

Here $$\nabla ^{{\mathbb {H}}}_{\mathcal {M}}=\pi _*I^* \nabla ^{{\mathbb {H}}}$$ is the connection on the bundle $$\mathcal {D}\rightarrow {\mathcal {M}}'$$ obtained by restricting the connection in the cohomology bundle $$\nabla ^{{\mathbb {H}}}$$ over $$\mathcal {U}$$ (induced by $$\nabla ^\textrm{Harm}$$ of Sect.2.5.3) to the diagonal $A^{\prime }=A$ and harmonic tangent vectors, and passing to gauge-equivalence classes.

For the proof of Proposition 5.5 we will need the following lemma.

#### Lemma 5.6

Consider a path $$(A,A'_t)$$ in $$\mathcal {U}$$ passing through $\left(A,A^{\prime }\right)$ at t=0. Then the sum-over-trees map $$\varphi _{A,A'_t}:H^1_A\supset U\rightarrow \textrm{FC}'$$ satisfies

217 $$\begin{aligned} \frac{d}{dt}\Big |_{t=0}\varphi _{A,A'_t}({\textsf{a}})=d_{\varphi _{A,A'}({\textsf{a}})}\gamma + (d_{\textsf{a}}\varphi _{A,A'})(\beta ) \end{aligned}$$

for sufficiently small ${\textsf{a}}$. Here:

- γ is given by the sum over trees with *K* on the root and either one edge (or the root) marked by $\Lambda _{{\dot{A}}'}$ or one leaf marked by ${\mathbb {I}}_{{\dot{A}}'}i({\textsf{a}})$. One also allows for γ a special graph consisting of a single marked leaf with value ${\mathbb {I}}_{{\dot{A}}'}i({\textsf{a}})$.
- $\beta =\{\Xi _{\delta A'={\dot{A}}'},-\}$ is given by the sum over trees with *p* on the root, with one edge marked by $\Lambda _{{\dot{A}}'}$ or one leaf marked by ${\mathbb {I}}_{{\dot{A}}'}i({\textsf{a}})$ or the root marked by $p{\mathbb {P}}_{{\dot{A}}'}$.

Note that the first term in the r.h.s. of (217) is an infinitesimal gauge transformation.

#### Sketch of proof

We have $\varphi _{A,A'_t}({\textsf{a}})=A+\delta _{A,A'_t}({\textsf{a}})$, with δ being the sum over trees with *K* on the root. Therefore, the l.h.s. of (217) is the sum over trees with one edge (or the root) marked by $[d,\Lambda ] +{\mathbb {I}}P+P{\mathbb {P}}$ or one leaf marked by $d{\mathbb {I}}i$ (we suppress the subscripts, in particular, $d=d_A$). As in the proof of Proposition 4.11, using the Stokes’ theorem on the configuration space to move *d* from the marked edge or leaf to other edges, we obtain a sum of:

1. Trees with one edge (or root) marked by Λ or one leaf marked by ${\mathbb {I}}i$ and one other edge marked by [d,K]=1-P.
2. Trees with one edge marked by Λ or one leaf marked by ${\mathbb {I}}i$ and the root marked by Kd=1-P-dK.
3. Trees with the root marked by dΛ.
4. Trees with one edge (or root) decorated by ${\mathbb {I}}P$.
5. Trees with one edge (or root) decorated by $P{\mathbb {P}}$.

Contributions of 1 on the edge from (1) cancel out in the sum over graphs by the classical master equation (IHX relation). Contributions of *P* on the edge from (1) and (2), together with (4) and (5), add up to the second term in the r.h.s. of (217): (a) if the subtree between *P* and the leaves does not contain ${\mathbb {I}},\Lambda$, such subtrees add up to zero by smoothness, and the same applies to (4); (b) if the subtree does contain ${\mathbb {I}}$ or Λ, then such subtrees, as well as the ones coming from with (5), add up to β. The remaining contributions are: *dK* from (2) and dΛ from (3) on the root add up to dγ; 1 from (2) yields $[\delta _{A,A'}({\textsf{a}}),\gamma ]$ (from pairs of trees joined at the root, one tree containing Λ or ${\mathbb {I}}$ and one not). Thus, we obtain

218 $$\begin{aligned} \frac{d}{dt}\Big |_{t=0}\varphi _{A,A'_t}({\textsf{a}})=d\gamma +[\delta _{A,A'}({\textsf{a}}),\gamma ]+(d_{\textsf{a}}\varphi _{A,A'})(\beta )= d_{\varphi _{A,A'}({\textsf{a}})}\gamma +(d_{\textsf{a}}\varphi _{A,A'})(\beta ). \end{aligned}$$

□

#### Proof of Proposition 5.5

Fix $A\in \textrm{FC}'$ and ${\textsf{a}}\in H^1_A$ sufficiently small. Consider the first-order deformation $A\rightarrow \widetilde{A}=A+\epsilon i(\alpha )$ with $\alpha \in H^1_A$. Then the infinitesimal parallel transport of $\nabla ^G$, seen as a fiber bundle connection on a neighborhood of the zero-section in $T{\mathcal {M}}'$ (Definition 2.25), maps ${\textsf{a}}\rightarrow \widetilde{{\textsf{a}}}$ where $\widetilde{\textsf{a}}$ is determined by

219 $$\begin{aligned} {\underline{\varphi }}_A({\textsf{a}})={\underline{\varphi }}_{\widetilde{A}}(\widetilde{{\textsf{a}}}). \end{aligned}$$

We will write $\widetilde{{\textsf{a}}}=B({\textsf{a}}+\epsilon {\textsf{b}})$ (all computations are $\bmod \,\epsilon ^2$), with $B={\mathfrak {B}}^1_{\widetilde{A}\leftarrow A;\widetilde{A}}:H^1_A\rightarrow H^1_{\widetilde{A}}$ the cohomology comparison map.^64^ The r.h.s. of Eq. (219) reads

220 $$\begin{aligned}&[\varphi _{\widetilde{A},\widetilde{A}}(\widetilde{\textsf{a}})] \underset{\mathrm {Prop.\;}2.41}{=} [\varphi _{A,\widetilde{A}}(B^{-1}(\widetilde{\textsf{a}})+\epsilon \alpha )] \end{aligned}$$

221 $$\begin{aligned}&\quad = [\varphi _{A,\widetilde{A}}({\textsf{a}})+\epsilon (d_{\textsf{a}}\varphi _{A,A})(\alpha +{\textsf{b}})] \end{aligned}$$

222 $$\begin{aligned}&\quad \underset{\mathrm {Lemma\;}5.6}{=}[\varphi _{A,A}({\textsf{a}})+\epsilon (d_{\textsf{a}}\varphi _{A,A})(\alpha +{\textsf{b}}+\beta )+\underbrace{\epsilon d_{\varphi _{A,A}({\textsf{a}})}(\gamma )}_{\mathrm {gauge\;transf.}}] \end{aligned}$$

223 $$\begin{aligned}&\quad =[\varphi _{A,A}({\textsf{a}})+\epsilon (d_{\textsf{a}}\varphi _{A,A})(\alpha +{\textsf{b}}+\beta )]. \end{aligned}$$

Here $$\beta ,\gamma $$ are as in Lemma 5.6 with $A^{\prime }=A$ and $${\dot{A}}'=i(\alpha )$$. Thus, (219) holds if the O(ϵ) term in the last line vanishes, i.e., if $${\textsf{b}}=-\alpha -\beta $$ and thus

224 $$\begin{aligned} \widetilde{\textsf{a}}=B({\textsf{a}}-\epsilon (\alpha +\beta )). \end{aligned}$$

Therefore, as a fiber bundle connection on (a neighborhood of the zero-section in) $T{\mathcal {M}}'$,

225 $$\begin{aligned} \nabla ^G= \nabla ^{\mathbb {H}}_{{\mathcal {M}}}+\langle [\delta A],\frac{\partial }{\partial {\textsf{a}}} \rangle +\{\pi _* I^* \Xi _{\delta A'=i[\delta A]},-\}. \end{aligned}$$

Its action on half-densities is given by (216). □

#### Remark 5.7

When we consider $\nabla ^G$ as a fiber bundle connection on $T{\mathcal {M}}'$, ${\textsf{a}}={\textsf{a}}^1$ is always an element of $H^1_A$; second and third terms in (225) give a 1-form on ${\mathcal {M}}'$ valued in formal vector fields on $H^1_A$. On the other hand, when we allow $\nabla ^G$ to act on half-densities on $H^\bullet _A[1]$, we have the cotangent lift of that vector field, $\{\xi ,-\}$ (which involves ${\textsf{a}}^2$—the component $H^2_A$ of ${\textsf{a}}$) appearing in (216). The term $\Delta _{\textsf{a}}\xi$ arises from the natural action of a hamiltonian vector field on $H^\bullet _{A}[1]$ on a half-density.

### Almost horizontality of ${\underline{Z}}$ w.r.t. Grothendieck connection

#### Proposition 5.8

Let ${\underline{\varphi }} :U \subset T{\mathcal {M}}'\rightarrow {\mathcal {M}}'$ be the sum-over-trees exponential map (73) induced by the SDR $(i_{A},p_{A},K_{A})$. Fix *A* and small $\alpha \in H^1_{A}$, and let $B=d_\alpha {\underline{\varphi }}_{A}(\alpha ):H^1_{A}\rightarrow H^1_{{\underline{\varphi }}_A(\alpha )}$. Then, for ${\textsf{a}}\in H^1_{A}$ small, we have

226 $$\begin{aligned} \det (B^\vee )\circ {\underline{Z}}_{\underline{\varphi }_{A}(\alpha )} (B({\textsf{a}}))={\underline{Z}}_{A}(\alpha + {\textsf{a}}) -i\hbar \Delta _{\textsf{a}}R(A,\alpha ,{\textsf{a}}) , \end{aligned}$$

where

227 $$\begin{aligned} R(A,\alpha ,{\textsf{a}})= &  \det (B^\vee )\circ R_{\widetilde{A},A,\widetilde{A}}({\mathfrak {B}}({\textsf{a}})) \nonumber \\= &  \int _0^1 dt\, r_{\widetilde{A},A_t;{\dot{A}}_t}({\mathfrak {B}}({\textsf{a}}))\cdot \det (B^\vee )\circ Z_{\widetilde{A},A_t}(B({\textsf{a}})). \end{aligned}$$

Here: $R_{\widetilde{A},A,\widetilde{A}}$ is as in (132), $\widetilde{A}=\varphi _{A,A}(\alpha )$ (hence $[\widetilde{A}]={\underline{\varphi }}_{A}(\alpha )$), $A_t=\varphi _{A,A}(t\alpha )$ is a path from *A* to $\widetilde{A}$, ${\mathfrak {B}}={\mathfrak {B}}_{\widetilde{A}\leftarrow A,A}:H^\bullet _A\rightarrow H^\bullet _{\widetilde{A}}$ is the promotion of *B* to a map between full cohomology, *r* is as in Proposition 4.11.

#### Proof

This is an immediate consequence of Theorems 4.3 and 4.10. Indeed, we have

228 $$\begin{aligned} &  \det (B^\vee )\circ {\underline{Z}}_{\underline{\varphi }_{A}(\alpha )} (B({\textsf{a}}))=\det (B^\vee )\circ Z_{\widetilde{A},\widetilde{A}}(B({\textsf{a}})) \nonumber \\ &  \quad \underset{\mathrm {Theorem\,}4.10}{=} \det (B^\vee )\circ \Big ( Z_{\widetilde{A},A}(B({\textsf{a}}))-i\hbar (\Delta _{\textsf{b}}R_{\widetilde{A},A,\widetilde{A}}({\textsf{b}}))\Big |_{{\textsf{b}}=B({\textsf{a}})} \Big ) \nonumber \\ &  \quad \underset{\mathrm {Theorem\,}4.3}{=} Z_{A,A}(\alpha +{\textsf{a}})-i\hbar \Delta _{\textsf{a}}\Big (\det (B^\vee )\circ R_{\widetilde{A},A,\widetilde{A}}({\mathfrak {B}}({\textsf{a}}))\Big ). \end{aligned}$$

□

#### Corollary 5.9

Partition function ${\underline{Z}}$ is horizontal w.r.t. Grothendieck connection modulo a BV-exact term:

229 $$\begin{aligned} \nabla ^G {\underline{Z}}=-i\hbar \Delta _{\textsf{a}}({\underline{r}}\, {\underline{Z}}), \end{aligned}$$

where ${\underline{r}}$ is $r_{A,A;i[\delta A]}$ of Proposition 4.11 with trees removed. I.e., ${\underline{r}}$ is the sum of connected Feynman graphs with ≥1 loops, with one edge marked by $\Lambda _{i[\delta A]}$ or one leaf marked by ${\mathbb {I}}_{i[\delta A]}i({\textsf{a}})$.

#### Proof

Taking the derivative of (226) in α at α=0 one obtains the following:

230 $$\begin{aligned} \nabla ^{G,\textrm{triv}}{\underline{Z}}= -i\hbar \Delta _{\textsf{a}}( \pi _*I^*(r)\, {\underline{Z}}), \end{aligned}$$

where *r* is as in Proposition 4.11 and $\nabla ^{G,\textrm{triv}}:=\nabla ^{{\mathbb {H}}}_{\mathcal {M}}+\langle [\delta A],\frac{\partial }{\partial {\textsf{a}}}\rangle$. Adding $\Delta _{\textsf{a}}(\pi _* I^* \Xi _{\delta A'}\, {\underline{Z}})$ to both sides and using Proposition 5.5, we obtain (229). □

### Correcting ${\underline{Z}}$ to a global object. Definition of $Z^\textrm{glob}$.

#### Theorem 5.10

${\underline{Z}}$ can be modified by a BV-exact term (pointwise on the moduli space) to a global object. I.e., there exists a degree -1 element $\rho \in \Gamma ({\mathcal {M}}',\textrm{Dens}^{\frac{1}{2},\textrm{formal}}(H^\bullet _{A}[1]))$ such that^65^

231 $$\begin{aligned} Z^\textrm{mod}:= {\underline{Z}}+i\hbar \Delta _{\textsf{a}}\rho \end{aligned}$$

satisfies

232 $$\begin{aligned} \nabla ^G Z^\textrm{mod}=0. \end{aligned}$$

For the proof (and for the proof of Proposition 5.19 below) we will need the following lemma.

#### Lemma 5.11

- Let
  233 $$\begin{aligned} {\overline{Z}}:=\pi _*I^* (e^{-\frac{i}{\hbar }\Xi }{\check{Z}}) \quad \in \Omega ^\bullet (\textrm{Met}\times {\mathcal {M}}',\mathcal {D}) \end{aligned}$$
  and
  234 $$\begin{aligned} {\overline{Z}}^\textrm{ren}:= e^{\frac{i}{\hbar }c(\hbar ) \frac{S_\textrm{grav}(g,\phi )}{2\pi }} {\overline{Z}}. \end{aligned}$$
  Then $${\overline{Z}}^\textrm{ren}$$ satisfies
  235 $$\begin{aligned} (\delta _g+\nabla ^G+\{\mu ,-\}+\Delta _{\textsf{a}}\mu -i\hbar \Delta _{\textsf{a}}) {\overline{Z}}^\textrm{ren}=0. \end{aligned}$$
  Here $$\mu =\pi _*I^* \Xi _{\delta g}\in \Omega ^{1,0}(\textrm{Met}\times {\mathcal {M}}',\widehat{\textrm{Sym}}(H_A[1])^*)$$.
- Let
  236 $$\begin{aligned} \widetilde{Z}:= {\overline{Z}}|_{g\mathrm {\;fixed,\;}\delta g=0}\; \in \Omega ^\bullet ({\mathcal {M}}',\mathcal {D}) \end{aligned}$$
  – the restriction of ${\overline{Z}}$ to a *g*-fixed slice of $\textrm{Met}\times {\mathcal {M}}'$.^66^ Then $\widetilde{Z}$ satisfies
  237 $$\begin{aligned} (\nabla ^G-i\hbar \Delta _{\textsf{a}})\widetilde{Z}=0. \end{aligned}$$

#### Proof

Denote $\xi ^\textrm{tot}:= \pi _*I^* \Xi =\xi +\mu$. The dQME (192) implies

238 $$\begin{aligned} &  0=\pi _*I^* e^{-\frac{i}{\hbar }\Xi }\underbrace{(\nabla ^\mathcal {D}-i\hbar \Delta _{\textsf{a}}-\frac{i}{\hbar }\frac{1}{2} \langle {\textsf{a}}, F_{\nabla ^{\mathbb {H}}}{\textsf{a}}\rangle )}_{\nabla ^{G-M}} (e^{\frac{i}{\hbar }\Xi }(e^{-\frac{i}{\hbar }\Xi } {\check{Z}}^\textrm{ren}) ) \nonumber \\ &  \quad = \Big (\nabla ^{\mathbb {H}}_{\textrm{Met}\times {\mathcal {M}}'}+\{\xi ^\textrm{tot},-\}+\Delta _{\textsf{a}}\xi ^\textrm{tot}-i\hbar \Delta _{\textsf{a}}+ \nonumber \\ &  \qquad +\frac{i}{\hbar }(\nabla ^{\mathbb {H}}_{\textrm{Met}\times {\mathcal {M}}'}\xi ^\textrm{tot}+\frac{1}{2} \{\xi ^\textrm{tot},\xi ^\textrm{tot}\}-\frac{1}{2} \langle {\textsf{a}}, F_{\nabla ^{\mathbb {H}}}{\textsf{a}}\rangle +\underbrace{\frac{1}{2}\langle [{\textsf{a}},{\textsf{a}}],KHi[\delta A] \rangle }_Y) \Big ) {\overline{Z}}^\textrm{ren}.\nonumber \\ \end{aligned}$$

Here the differential operator in brackets in lines 2 and 3—except the term *Y*—arises as a conjugation of the Gauss-Manin superconnection by $$e^{\frac{i}{\hbar }\Xi }$$, restricted to $A^{\prime }=A$ and $$\delta A'=\delta A$$ harmonic and reduced modulo gauge transformations.

Next we comment on the *Y* term appearing in (238). First consider for simplicity the case of *g* fixed. In fact, $I^* (\nabla ^{G-M} {\check{Z}}^\textrm{ren})$ cannot be computed a priori as a differential operator (denote it $I^*\nabla ^{G-M}$) acting on $I^*{\check{Z}}^\textrm{ren}$. The reason is that when we move infinitesimally on $\textrm{FC}' {\mathop {\hookrightarrow }\limits ^{\textrm{Diag}}}\textrm{FC}'\times \textrm{FC}'$, the notion of harmonic forms changes, so, in order to evaluate $I^* (\nabla ^{G-M} {\check{Z}}^\textrm{ren})$ on a tuple of harmonic tangent vectors, it is insufficient to know $I^*({\check{Z}}^\textrm{ren})$—one needs also the value of ${\check{Z}}^\textrm{ren}$ on a collection of harmonic tangent vectors and one non-harmonic (arising as a variation of a harmonic tangent vector).

More precisely: given harmonic tangent vectors $\alpha _0,\ldots ,\alpha _p \in T_{(A,A)}\textrm{FC}'_\textrm{Diag}$, we extend them to harmonic vector fields $\widetilde{\alpha }_0,\ldots ,\widetilde{\alpha }_p$ on $\textrm{FC}'_\textrm{Diag}$. Then we have

239 $$\begin{aligned} &  (\nabla ^\mathcal {D}{\check{Z}}^\textrm{ren})(\widetilde{\alpha }_0,\ldots ,\widetilde{\alpha }_p)= \sum _{i=0}^p \nabla ^\mathcal {D}_{\widetilde{\alpha }_i}{\check{Z}}^\textrm{ren}(\widetilde{\alpha }_0,\ldots \widehat{\widetilde{\alpha }_i}\ldots , \widetilde{\alpha }_p) \nonumber \\ &  \quad - \sum _{i,j=0}^p \frac{1}{2} {\check{Z}}^\textrm{ren}([\widetilde{\alpha }_i,\widetilde{\alpha }_j],\widetilde{\alpha }_0,\ldots \widehat{\widetilde{\alpha }_i}\ldots \widehat{\widetilde{\alpha }_j}\ldots ,\widetilde{\alpha }_p). \end{aligned}$$

Then, choosing the extensions $\widetilde{\alpha }_i|_{A+\epsilon \beta }=\alpha _i-\epsilon (K \textrm{ad}_\beta \alpha _i+dG \textrm{ad}^*_{\beta }\alpha _i)+O(\epsilon ^2)$ (cf. (86), (87)), we have $[\widetilde{\alpha }_i,\widetilde{\alpha }_j]|_{(A,A)}=2dG*[\alpha _i,*\alpha _j]$. Hence, we have

240 $$\begin{aligned} I^* \nabla ^{G-M} {\check{Z}}^\textrm{ren}=(I^* \nabla ^{G-M}) (I^* {\check{Z}}^\textrm{ren})+I^*(\langle -d G \textrm{ad}^*_{\delta A}\delta A,\frac{\delta }{\delta (\delta A)} \rangle {\check{Z}}^\textrm{ren} ) \end{aligned}$$

Here the two terms on the right corresponds to the two terms on the right in (239). Allowing *g* to vary (and so allowing the tangent vectors to have component along $T_g\textrm{Met}$), the obvious extension of this computation yields

241 $$\begin{aligned} I^* \nabla ^{G-M} {\check{Z}}^\textrm{ren}=(I^* \nabla ^{G-M}) (I^* {\check{Z}}^\textrm{ren})+I^*(\langle d K\underbrace{H}_{H_{\delta A'}+H_{\delta g}}\delta A,\frac{\delta }{\delta (\delta A)} \rangle {\check{Z}}^\textrm{ren} ) \end{aligned}$$

In terms of the Feynman diagram expansion of ${\check{Z}}$ of Remark 4.22, the rightmost term in (241) has the following contributions:

(i) Hi[δA] replacing a gray vertex in a graph (from the derivation acting on δA in a gray vertex),
(ii) replacing a white vertex in a graph by $\textrm{ad}_{KHi[\delta A]}$ (from the derivation acting on $\delta A'=\delta A$ in a white vertex).

**Fig. 12:**
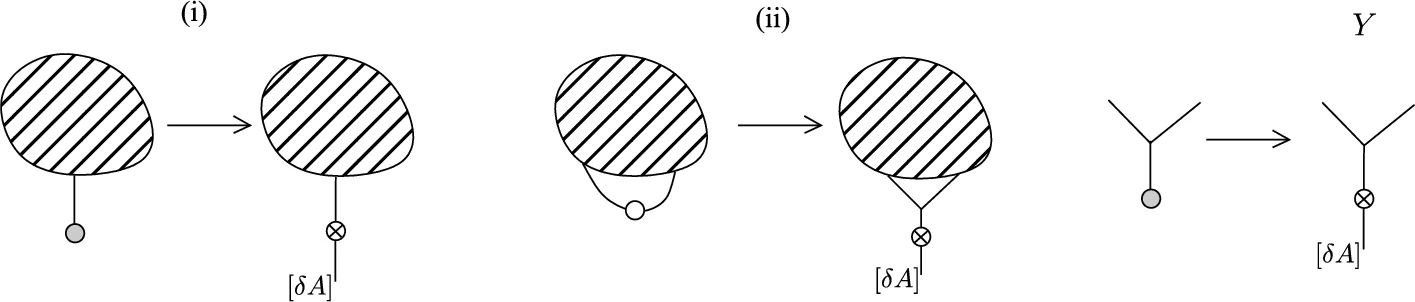
Cancellation mechanism in the rightmost term in (241). Circle vertex with a cross stands for either white or black vertex. Dashed region represents some graph

Contributions (i) and (ii) above mutually cancel, except for one special graph in (i)—a cubic corolla with one leaf attached to a gray vertex, replacing the latter with Hi[δA] as in (i) above, see Fig. 12. This yields $Y {\check{Z}}^\textrm{ren}$, resulting eventually in the *Y* term correction in (238).

Now that (238) is proven, we show that it can be simplified. Consider the dQME (192) on ${\check{Z}}^\textrm{ren}$, restrict it to lowest order in ħ (tree diagram contributions) and to 2-forms on $\underline{\mathcal {U}}$. Then, reducing to $\textrm{Met}\times {\mathcal {M}}'$ by applying $\pi _*I^*$, we obtain the equation

242 $$\begin{aligned} \nabla ^{\mathbb {H}}_{\textrm{Met}\times {\mathcal {M}}'}\xi ^\textrm{tot}+\frac{1}{2} \{\xi ^\textrm{tot},\xi ^\textrm{tot}\}-\frac{1}{2} \langle {\textsf{a}}, F_{\nabla ^{\mathbb {H}}}{\textsf{a}}\rangle +Y =0 \end{aligned}$$

where the *Y* term appears by the same mechanism as above. Hence, (238) simplifies to

243 $$\begin{aligned} (\delta _g+\underbrace{\nabla ^{\mathbb {H}}_{\mathcal {M}}+\{\xi ,-\}+\Delta _{\textsf{a}}\xi }_{\nabla ^G} +\{\mu ,-\}+\Delta _{\textsf{a}}\mu -i\hbar \Delta _{\textsf{a}}){\overline{Z}}^\textrm{ren}=0, \end{aligned}$$

which proves (235).

Finally, (237) is an immediate consequence of (235) by restricting to a *g*-fixed slice of $\textrm{Met}\times {\mathcal {M}}'$. □

#### Proof of Theorem 5.10

**Step 1.** (Building a chain contraction for $\nabla ^G-i\hbar \Delta _{\textsf{a}}$.) The cohomology of the complex $(\Omega ^\bullet ({\mathcal {M}}', \mathcal {D} ),\nabla ^G)$ is concentrated in form degree 0 and is isomorphic to global half-densities $\textrm{Dens}^{\frac{1}{2}}(T^*[-1]{\mathcal {M}}')$.^67^ More precisely, one has SDR data $({\textsf{i}},{\textsf{p}},{\textsf{K}})$ with inclusion ${\textsf{i}}=T{\underline{\varphi }}^*$ and projection ${\textsf{p}}$ given by evaluating a formal half-density at ${\textsf{a}}^1=0$,

244 $$\begin{aligned} {\textsf{p}}: \psi ([A],{\textsf{a}}^1,{\textsf{a}}^2)D^{\frac{1}{2}} {\textsf{a}}^1 D^{\frac{1}{2}}{\textsf{a}}^2\mapsto \psi ([A],0,{\textsf{a}}^2) D^{\frac{1}{2}} [A] D^{\frac{1}{2}}{\textsf{a}}^2 \end{aligned}$$

where ${\textsf{a}}^1,{\textsf{a}}^2$ are the components of ${\textsf{a}}$ in $H^1_A=T_{[A]}{\mathcal {M}}'$ and $H^2_A=T^*_{[A]}[-1] {\mathcal {M}}'$. It is also understood that ${\textsf{p}}$ sends forms of positive degree on ${\mathcal {M}}'$ to zero.

Next, deform the differential on $\Omega ^\bullet ({\mathcal {M}}', \mathcal {D} )$ from $\nabla ^G$ to $\nabla ^G-i\hbar \Delta _{\textsf{a}}$. By homological perturbation lemma (Lemma A.2), one has deformed SDR data

245 $$\begin{aligned} ({\textsf{i}}',{\textsf{p}}',{\textsf{K}}'):(\Omega ^\bullet ({\mathcal {M}}', \mathcal {D} ), \nabla ^G -i\hbar \Delta _{\textsf{a}}) \leadsto (\textrm{Dens}^{\frac{1}{2}}(T^*[-1]{\mathcal {M}}'),\delta ). \end{aligned}$$

Moreover, the fact that ${\textsf{K}}$ lowers form degree along ${\mathcal {M}}'$ by one, implies that

- the induced differential $\delta =-i\hbar \Delta$ is the BV Laplacian on (global) half-densities $T^*[-1]{\mathcal {M}}'$,
- $${\textsf{i}}'={\textsf{i}}$$.

**Step 2.** Using the fact that $K^{\prime }$ defined above satisfies the chain homotopy property $$\textrm{id}={\textsf{i}}{\textsf{p}}'+[\nabla ^G-i\hbar \Delta _{\textsf{a}},{\textsf{K}}']$$, we have

246 $$\begin{aligned} \widetilde{Z}={\textsf{i}}{\textsf{p}}'\widetilde{Z}+(\nabla ^G-i\hbar \Delta _{\textsf{a}}) {\textsf{K}}' \widetilde{Z}+ {\textsf{K}}'\underbrace{(\nabla ^G-i\hbar \Delta _{\textsf{a}})\widetilde{Z}}_{=0\mathrm {\; by\; (237)}} , \end{aligned}$$

where $\widetilde{Z}$ is as in (236). Denote the first term on the r.h.s. by $Z^\textrm{mod}:= {\textsf{i}}{\textsf{p}}'\widetilde{Z}$. Since it is in the image of ${\textsf{i}}$, it is $\nabla ^G$-closed, and hence a global object. Restricting (246) to form degree zero along ${\mathcal {M}}'$ and denoting

247 $$\begin{aligned} \rho =({\textsf{K}}' \widetilde{Z})|_{\Omega ^0({\mathcal {M}}',\mathcal {D})}, \end{aligned}$$

we obtain (231). □

#### Lemma 5.12

We have the following ansatz for ħ-dependence of ρ and $Z^\textrm{mod}$:

248 $$\begin{aligned} \rho= &  {\underline{Z}}\cdot f(\hbar ), \end{aligned}$$

249 $$\begin{aligned} Z^\textrm{mod}= &  {\underline{Z}}\cdot (1+\hbar \, g(\hbar )), \end{aligned}$$

where $f,g\in C^\infty ({\mathcal {M}}',\underbrace{\widehat{\textrm{Sym}}(H_A[1])^*}_{{\mathbb {F}}})[[\hbar ]]$ are some formal power series in ħ and ${\textsf{a}}$.

#### Proof

Both properties follow from the fact that

250 $$\begin{aligned} \widetilde{Z}=\underbrace{e^{\frac{i}{\hbar }S_{CS}(A)}\tau _A^{\frac{1}{2}}e^{\frac{\pi i}{4}\psi _A}}_{{\underline{Z}}_0}\,e^{\frac{i}{\hbar }\omega +h} \end{aligned}$$

with $\omega \in \Omega ^{\ge 2}({\mathcal {M}}',{\mathbb {F}})$ independent of ħ and with $h\in \Omega ^{\ge 0}({\mathcal {M}}',{\mathbb {F}})[[\hbar ]]$. Hence, the *p*-form component of $\widetilde{Z}$ along ${\mathcal {M}}'$ is

251 $$\begin{aligned} \widetilde{Z}^{(p)}={\underline{Z}}_0\cdot \hbar ^{-\left[ \frac{p}{2}\right] }F^{(p)}={\underline{Z}}\cdot \hbar ^{-\left[ \frac{p}{2}\right] }F^{'(p)} \end{aligned}$$

with $$F^{(p)},F^{'(p)}\in \Omega ^p({\mathcal {M}}',{\mathbb {F}})[[\hbar ]]$$. Therefore,

252 $$\begin{aligned} \rho ={\textsf{K}}'\widetilde{Z}\big |_{\Omega ^0({\mathcal {M}}',\mathcal {D})}=\sum _{k\ge 0}({\textsf{K}} i\hbar \Delta _{\textsf{a}})^k {\textsf{K}}\left( \widetilde{Z}|_{\Omega ^{k+1}({\mathcal {M}}',\mathcal {D})}\right) = {\underline{Z}}_0 \cdot \sum _{k\ge 0}\hbar ^{k-\left[ \frac{k+1}{2}\right] }G_k \end{aligned}$$

with $G_k\in \Omega ^0({\mathcal {M}}',{\mathbb {F}})[[\hbar ]]$. This implies (248) and—using (231)—implies also (249).

□

#### Definition 5.13

We define the global partition function as the degree zero half-density on $T^*[-1]{\mathcal {M}}'$ (or, equivalently, a volume form on ${\mathcal {M}}'$) given by restriction $Z^\textrm{mod}(A,{\textsf{a}})$ to ${\textsf{a}}=0$:

253 $$\begin{aligned} Z^\textrm{glob}:= Z^\textrm{mod}\Big |_{{\textsf{a}}=0} \in \textrm{Dens}^{\frac{1}{2}}(T^*[-1]{\mathcal {M}}'). \end{aligned}$$

In the notations of the proof of Theorem 5.10, we have

254 $$\begin{aligned} Z^\textrm{glob}={\textsf{p}}'\widetilde{Z}. \end{aligned}$$

Lemma 5.12 above implies

255 $$\begin{aligned} Z^\textrm{glob}={\underline{Z}}\big |_{{\textsf{a}}=0}\,(1+O(\hbar )). \end{aligned}$$

#### Proposition 5.14

One has

256 $$\begin{aligned} \begin{aligned} Z^\textrm{glob}&=\sum _{k=0}^{\dim {\mathcal {M}}'} \frac{(-i\hbar )^k}{k!} \left\langle \frac{\partial }{\partial {\textsf{a}}^2},\frac{\partial }{\partial [\delta A]}\right\rangle ^k \widetilde{Z}^{(k)}\Big |_{{\textsf{a}}^1=0}\\&=\left( e^{-i\hbar \left\langle \frac{\partial }{\partial {\textsf{a}}^2},\frac{\partial }{\partial [\delta A]}\right\rangle } \widetilde{Z} \right) \Big |_{{\textsf{a}}^1=[\delta A]=0} . \end{aligned} \end{aligned}$$

Here $\dim {\mathcal {M}}'=\dim H^1_A$ is the dimension of the connected component of ${\mathcal {M}}'$ containing [*A*]; $\widetilde{Z}^{(k)}$ is the *k*-form component of $\widetilde{Z}$, as a form on ${\mathcal {M}}'$.

#### Proof

In the notations of the proof of Theorem 5.10, we have

257 $$\begin{aligned} Z^\textrm{glob}={\textsf{p}}'\widetilde{Z}=\sum _{k\ge 0} {\textsf{p}}(i\hbar \Delta _{\textsf{a}}{\textsf{K}})^k \widetilde{Z}. \end{aligned}$$

The chain homotopy ${\textsf{K}}$ increases the polynomial degree in ${\textsf{a}}^1$, and in the lowest degree in ${\textsf{a}}^1$ is given by

258 $$\begin{aligned} {\textsf{K}}\omega |_{{\textsf{a}}^1\rightarrow 0} \sim -\frac{1}{\deg \omega } \left\langle {\textsf{a}}^1,\frac{\partial }{\partial [\delta A]}\right\rangle \omega |_{{\textsf{a}}^1=0}, \end{aligned}$$

cf. the homotopy $\delta ^*$ in [BCM12, Section 2]. On the other hand $\Delta _{\textsf{a}}$ lowers the degree in ${\textsf{a}}^1$ by one and *p* sets ${\textsf{a}}^1$ to zero. So, in the r.h.s. of (257), only the constant term in ${\textsf{a}}^1$ contributes, and for the purpose of evaluating the r.h.s., ${\textsf{K}}$ can be replaced by its asymptotics (258). Formula (256) follows. □

#### Corollary 5.15

Global partition function $Z^\textrm{glob}$ is related to the perturbative partition function ${\underline{Z}}$ by

259 $$\begin{aligned} (T{\underline{\varphi }}^* Z^\textrm{glob})(A,{\textsf{a}})={\underline{Z}}_A({\textsf{a}})+i\hbar \Delta _{\textsf{a}}\rho (A,{\textsf{a}}). \end{aligned}$$

#### Proof

This is an immediate consequence of (246) restricted to form degree zero along ${\mathcal {M}}'$. □

#### Remark 5.16

*(A path integral formula for* $Z^\textrm{glob}$*)* Formula (256) can be seen as the perturbative evaluation of the following path integral:^68^

260 $$\begin{aligned} &  Z^\textrm{glob}(A)= \int _{H^2_A[-1]\oplus H^1_A[1]} \mathcal {D} {\textsf{a}}^2\, \mathcal {D} \zeta \int _{\mathcal {L}=\Omega _{d_A^*\mathrm {-ex}}[1]} \mathcal {D} \alpha _\textrm{fl} \nonumber \\ &  \quad \exp \frac{i}{\hbar }\Bigg (S_{CS}(A+i({\textsf{a}}^2)+\alpha _\textrm{fl})+ \langle \zeta ,{\textsf{a}}^2\rangle \nonumber \\ &  \quad + \int _M \frac{1}{2} \langle \alpha _\textrm{fl},d_A G \textrm{ad}^*_{i(\zeta )} \alpha _\textrm{fl} \rangle +\langle \alpha _\textrm{fl},d_A G \textrm{ad}^*_{i(\zeta )} i({\textsf{a}}^2) \rangle \Bigg ). \end{aligned}$$

The last two terms can also be written as

261 $$\begin{aligned} \int _M \frac{1}{2} \langle i({\textsf{a}}^2)+\alpha _\textrm{fl}, H_{\delta A'=i(\zeta )}(i({\textsf{a}}^2)+\alpha _\textrm{fl}) \rangle , \end{aligned}$$

with $H_{\delta A'}$ as in (179b). Note that in the integral formula (260), ${\textsf{a}}^2$ and $\zeta =[\delta A]$ become dynamical variables (integrated over).

**Fig. 13:**
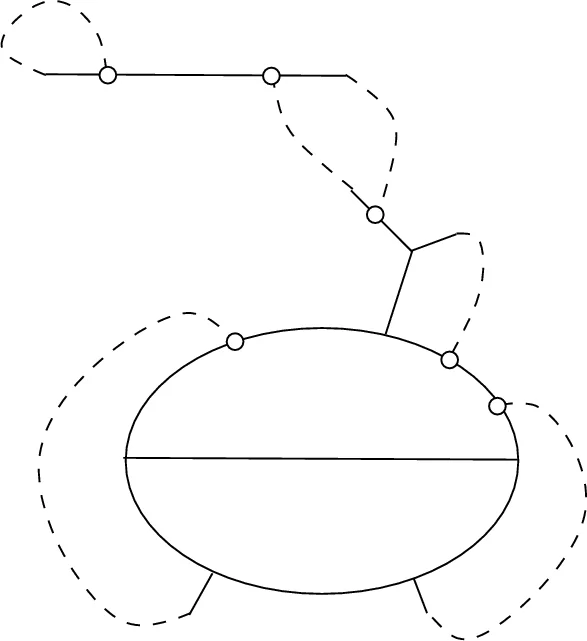
Example of a Feynman graph for $Z^\textrm{glob}$ (260). Dashed edges correspond to ${\textsf{a}}^2$–ζ propagators; white vertices correspond to (261). Selection rules: ≤2 white vertices on a solid edge, $\le \dim {\mathcal {M}}'$ dashed edges in total. A solid edge not incident to Chern–Simons cubic vertices should have exactly two white vertices (as in the top part of the picture)

The Feynman graph expansion of $Z^\textrm{glob}$ has the form262The graphs shown contribute in the order O(ħ) and ⋯ is of order ≥2 in ħ, with graphical conventions as in Fig. 13.

### Metric dependence of the global partition function

To define $Z^\textrm{glob}$ we needed to choose a metric *g* on *M*. In this section we analyze the dependence of $Z^\textrm{glob}$ on this metric. We have shown previously that $Z^\textrm{glob}$ can be interpreted either as a top form on ${\mathcal {M}}'$ or, equivalently, a half-density on $T^*[-1]{\mathcal {M}}'$ that does not depend on the fiber coordinates (has degree zero). As such it is trivially closed (w.r.t. de Rham differential or BV Laplacian).

Let

263 $$\begin{aligned} Z^\textrm{glob,ren}_{g,\phi }(A):= e^{\frac{i}{\hbar }c(\hbar )\frac{S_\textrm{grav}(g,\phi )}{2\pi }} Z^\textrm{glob}_g(A) \end{aligned}$$

be the renormalized global partition function, with c(ħ) as in (163) and ϕ a framing of *M*.

The main result of this section is the following theorem.

#### Theorem 5.17

The cohomology class of $Z_{g,\phi }^\textrm{glob,ren}$ is independent of the choice of metric *g*.

#### Definition 5.18

We call the cohomology class $[Z^\textrm{glob,ren}_{g,\phi }] \in H^\textrm{top}({\mathcal {M}}')$ the *Chern–Simons volume class* on ${\mathcal {M}}'$.

Consider ${\overline{Z}}^\textrm{ren}$ defined by (234) and let

264 $$\begin{aligned} {\check{Z}}^\textrm{glob,ren}:={\textsf{p}}' {\overline{Z}}^\textrm{ren} \quad \in \Omega ^\bullet (\textrm{Met})\otimes \textrm{Dens}^{\frac{1}{2}}(T^*[-1]{\mathcal {M}}') \end{aligned}$$

with ${\textsf{p}}'$ as in (245). Note that ${\check{Z}}^\textrm{glob,ren}$ is an extension of $Z^\textrm{glob,ren}$ to a nonhomogeneous form on $\textrm{Met}$; we denote its *k*-form component by ${\check{Z}}^{\textrm{glob,ren}(k)}$.

#### Proposition 5.19

${\check{Z}}^\textrm{glob,ren}$ satisfies the following.

265 $$\begin{aligned} (\delta _g-i\hbar \Delta ){\check{Z}}^\textrm{glob,ren}=0, \end{aligned}$$

266 $$\begin{aligned} \delta _g Z^\textrm{glob,ren} = i\hbar \Delta {\check{Z}}^{\textrm{glob,ren}(1)}, \end{aligned}$$

267 $$\begin{aligned} \begin{aligned} {\check{Z}}^\textrm{glob,ren}&= \sum _{k=0}^{\dim {\mathcal {M}}'}\frac{(-i\hbar )^k}{k!} \left\langle \frac{\partial }{\partial {\textsf{a}}^2},\frac{\partial }{\partial [\delta A]} \right\rangle ^k {\overline{Z}}^{\textrm{ren}(\bullet ,k)}\Big |_{{\textsf{a}}^1=0} \\&= \left( e^{-i\hbar \left\langle \frac{\partial }{\partial {\textsf{a}}^2},\frac{\partial }{\partial [\delta A]} \right\rangle }{\overline{Z}}^\textrm{ren}\right) \Big |_{{\textsf{a}}^1=[\delta A]=0}. \end{aligned} \end{aligned}$$

Here Δ is the BV Laplacian on half-densities on $T^*[-1]{\mathcal {M}}'$. Superscript (∙,k) means the component of de Rham degree *k* along ${\mathcal {M}}'$ (and arbitrary degree along $\textrm{Met}$).

Note that (266) immediately implies Theorem 5.17.

#### Proof

First, recall from Lemma 5.11 that ${\overline{Z}}^\textrm{ren}$ satisfies

268 $$\begin{aligned} (\delta _g+\nabla ^G+\{\mu ,-\}+\Delta _{\textsf{a}}\mu -i\hbar \Delta _{\textsf{a}}) {\overline{Z}}^\textrm{ren}=0. \end{aligned}$$

Next, consider the differential perturbation of the contraction

269 $$\begin{aligned} &  ({\textsf{i}},{\textsf{p}},{\textsf{K}}):(\Omega ^\bullet (\textrm{Met}\times {\mathcal {M}}',\mathcal {D},\nabla ^G) \nonumber \\ &  \quad \leadsto (\Omega ^\bullet (\textrm{Met})\otimes \textrm{Dens}^{\frac{1}{2}}(T^*[-1]{\mathcal {M}}'),\mathrm {zero\; differential}), \end{aligned}$$

deforming $\nabla ^G\rightarrow \nabla ^G+\underbrace{\delta _g+\{\mu ,-\}+\Delta _{\textsf{a}}\mu -i\hbar \Delta _{\textsf{a}}}_\varkappa$. By homological perturbation lemma, we obtain the deformed contraction

270 $$\begin{aligned} &  ({\textsf{i}}'',{\textsf{p}}''={\textsf{p}}',{\textsf{K}}''):(\Omega ^\bullet (\textrm{Met}\times {\mathcal {M}}',\mathcal {D}), \nabla ^G+\varkappa ) \nonumber \\ &  \quad \leadsto (\Omega ^\bullet (\textrm{Met})\otimes \textrm{Dens}^{\frac{1}{2}}(T^*[-1]{\mathcal {M}}'),\delta _g-i\hbar \Delta ). \end{aligned}$$

We remark that:

(i) The induced differential in (270) is $\sum _{k\ge 0}{\textsf{p}}\varkappa (-{\textsf{K}}\varkappa )^k {\textsf{i}}$. Since the image of ${\textsf{i}}$ is in zero-forms on ${\mathcal {M}}'$, ${\textsf{K}}$ reduces the form degree on ${\mathcal {M}}'$ by one and ϰ does not change the form degree on ${\mathcal {M}}'$, all terms with k>0 vanish and the induced differential is ${\textsf{p}}\varkappa {\textsf{i}}= \delta _g-i\hbar \Delta$.
(ii) One has ${\textsf{p}}''=\mathsf {p'}$—the chain projection in (245) extended trivially along $\textrm{Met}$. Indeed, one has
  271 $$\begin{aligned} {\textsf{p}}''=\sum _{k\ge 0}{\textsf{p}} (-\varkappa {\textsf{K}})^k. \end{aligned}$$
  Since ${\textsf{p}}$ evaluates the constant term in ${\textsf{a}}^1$, ${\textsf{K}}$ increases the degree in ${\textsf{a}}^1$ and $\varkappa =-i\hbar \Delta _{\textsf{a}}$ plus terms that do not decrease the degree in ${\textsf{a}}^1$, in (271) one can replace ϰ with $-i\hbar \Delta _{\textsf{a}}$, leading to ${\textsf{p}}''={\textsf{p}}'$.

Since ${\textsf{p}}'$ is a chain map, it sends the cocycle ${\overline{Z}}^\textrm{ren}$ of the complex upstairs in (270) to a cocycle ${\check{Z}}^\textrm{glob,ren}$ of the complex downstairs. This proves (265).

Equation (266) is the restriction of (265) to form degree 1 on $\textrm{Met}$.

Formula (267) for ${\check{Z}}^\textrm{glob,ren}$ is proven similarly to Proposition 5.14. □

#### Remark 5.20

The path integral formula for $Z^\textrm{glob}$ from Remark 5.16 extends—via (267)—to the extended global partition function ${\check{Z}}^\textrm{glob,ren}$ as follows:

272 $$\begin{aligned} &  {\check{Z}}^\textrm{glob,ren}(A,{\textsf{a}}^2_0)= e^{\frac{i}{\hbar }c(\hbar )\frac{S_\textrm{grav}(g,\phi )}{2\pi }} \int _{H^2_A[-1]\oplus H^1_A[1]} \mathcal {D} {\textsf{a}}^2_{\textrm{fl}}\, \mathcal {D} \zeta \nonumber \\ &  \quad \int _{\mathcal {L}=\Omega _{d_A^*\mathrm {-ex}}[1]} \mathcal {D} \alpha _\textrm{fl} \exp \frac{i}{\hbar }\Big (S_{CS}(A+i({\textsf{a}}^2)+\alpha _\textrm{fl})+\langle \zeta ,{\textsf{a}}^2_{\textrm{fl}}\rangle \nonumber \\ &  \quad + \int _M \underbrace{\frac{1}{2} \langle \alpha _\textrm{fl},d_A G \textrm{ad}^*_{i(\zeta )} \alpha _\textrm{fl} \rangle +\langle \alpha _\textrm{fl},d_A G \textrm{ad}^*_{i(\zeta )} i({\textsf{a}}^2) \rangle }_{-\frac{1}{2}\langle i({\textsf{a}}^2)+\alpha _\textrm{fl},H_{\delta A'=i(\zeta )}(i({\textsf{a}}^2)+\alpha _\textrm{fl}) \rangle } \nonumber \\ &  \quad \underbrace{-\frac{1}{2} \langle \alpha _\textrm{fl},\lambda _{\delta g} \alpha _\textrm{fl}\rangle - \langle \alpha _\textrm{fl},\lambda _{\delta g} i({\textsf{a}}^2)\rangle }_{-\frac{1}{2}\langle i({\textsf{a}}^2)+\alpha _\textrm{fl},H_{\delta g}(i({\textsf{a}}^2)+\alpha _\textrm{fl}) \rangle } \Big )\Bigg |_{{\textsf{a}}^2={\textsf{a}}^2_0+{\textsf{a}}^2_\textrm{fl}} \end{aligned}$$

with $H_{\delta A'}$ and $H_{\delta g}$ as in (179b), (179c); ${\textsf{a}}^2_0$ is interpreted as a vector in $T^*_A[-1]{\mathcal {M}}'$.

In particular, the Feynman diagram expansion of the generator ${\check{Z}}^{\textrm{glob,ren}(1)}$ in the r.h.s. of (266) is273Here the graphical conventions are as in Fig. 13; loose half-edges are decorated by $i({\textsf{a}}^2_0)$; black circle vertex is decorated by $H_{\delta g}$. The graphs shown contribute in zeroth order in ħ and ⋯ is of order ≥1 in ħ.

#### Relation to the asymptotic expansion conjecture

Now fix ${\mathfrak {g}}= {{\mathfrak {s}}}{{\mathfrak {u}}}(N)$ and denote $\tau _{k,N}$ the (*SU*(*N*)-Reshetikhin-Turaev invariants [RT91]. We recall the statement of the asymptotic expansion conjecture, which we cite from [And02, Conjecture 7.7]

##### Conjecture 5.21

Let $\{c_0,\ldots ,c_m\}$ be the Chern–Simons invariants of *M*. Then there exist $d_j \in {\mathbb {Q}}, {\tilde{I}}_j \in {\mathbb {Q}}/{\mathbb {Z}}, v_j \in {\mathbb {R}}_+$ and $a_j^e$ for j=0,…,m and $e \in {\mathbb {N}}$ such that for $r = k + h^\vee$:

274 $$\begin{aligned} \tau _{k,N} \underset{k\rightarrow \infty }{\sim }\sum _{j=0}^m e^{\frac{i r c_j}{ 2\pi }}r^{d_j}e^{\frac{i \pi }{4} {\tilde{I}}_j}v_j \exp \sum _{e=1}^\infty a_j^e \left( \frac{r}{2\pi }\right) ^{-e}. \end{aligned}$$

Other forms of this conjecture have appeared in the literature, for instance in [Res10, Section 6]. We conjecture that if $c_j$ comes from a union of smooth, irreducible components of the moduli space, then the *j*-th summand above coincides with the integral of Chern–Simons volume class over that preimage. More precisely, fix ϕ to be the canonical 2-framing of *M* and denote ${\mathcal {M}}_j = S_{CS}^{-1}(c_j)$. If ${\mathcal {M}}_j \subset {\mathcal {M}}'$, then we conjecture that $a_j^e$ is given by the coefficient of $\hbar ^e$ (contribution of connected (e+1)-loop graphs) in $\log \int _{{\mathcal {M}}_j}Z^\textrm{glob,ren}_{g,\phi }$. This assumes that at higher loop orders one has to identify $\hbar = \frac{2\pi }{k + h^\vee }$ (as discussed by Axelrod-Singer [AS91, Section 6]).

An interesting class of examples where this conjecture could potentially be checked are Seifert fibered homology spheres. For these, the only reducible connection is the trivial one, all other components of the moduli space are closed manifolds ( [FS90]). For this class, the asymptotic expansion conjecture has recently been proven in [And+25], where the authors show that the asymptotic expansion is given in terms of integrals over the smooth components of the moduli space. Comparison with this and other results will be addressed in future work.

## Figures and Tables

**Fig. 14:**
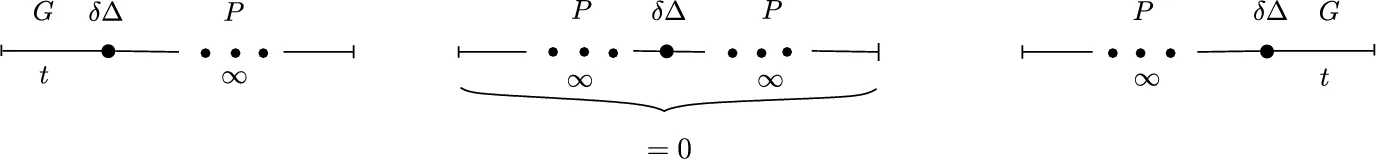
Terms in the formula (309) for δP correspond to splitting the interval by a point (a) close to the left endpoint, (c) far from both endpoints (the respective contribution is zero), (b) close to the right endpoint

**Fig. 15:**
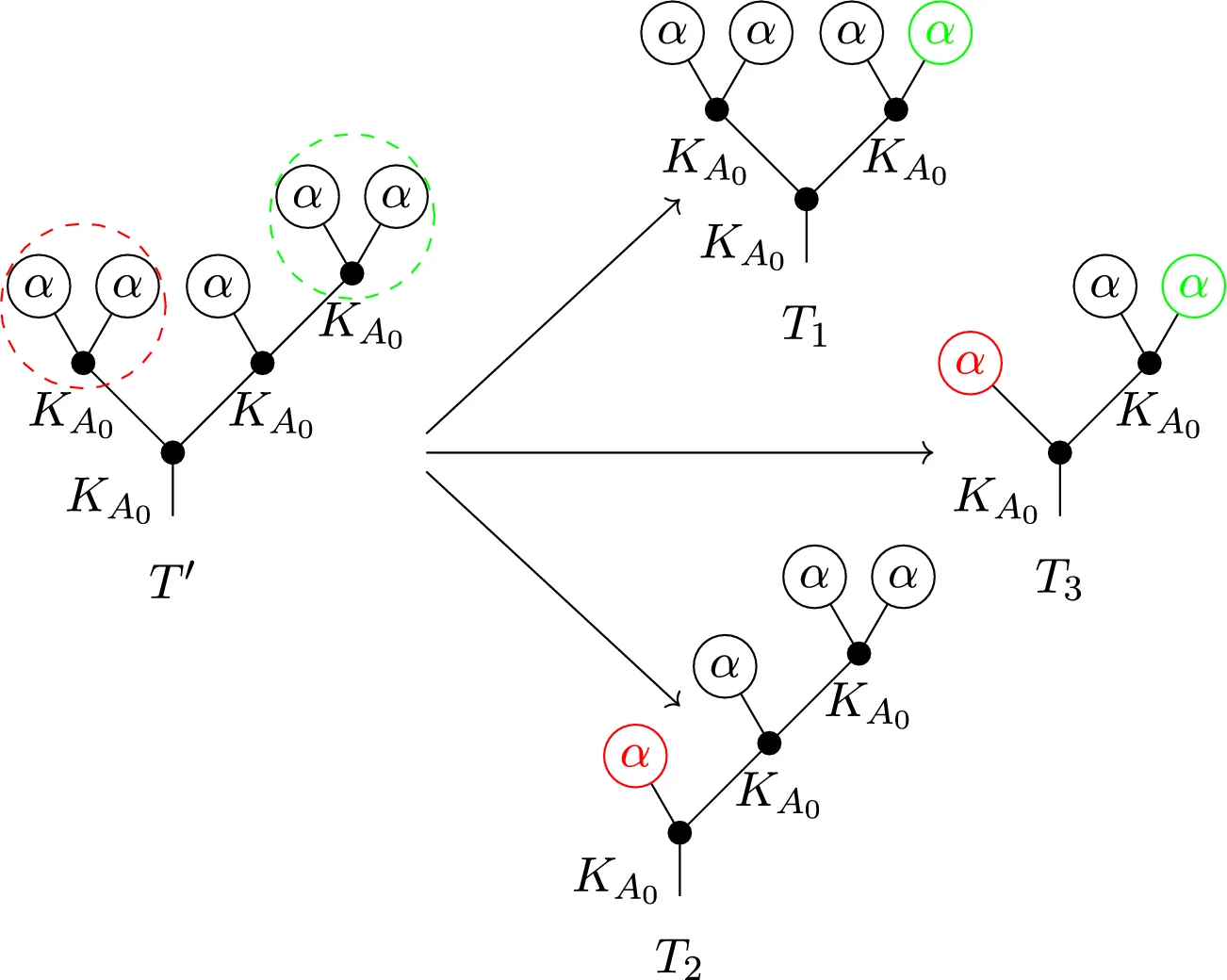
The tree $T^{\prime }$ has $$n_{T'} = 2 $$ corollas, collapsing the green one yields $$T_1$$, collapsing the red one yields $$T_2$$, collapsing both yields $$T_3$$. Thus it will appear in $$\widetilde{\delta }_{A_0}(\widetilde{\kappa }_{A_0}(\alpha ))$$ four times, with total combinatorial coefficient 1+(-1)+(-1)+1=0

## Data Availability

No data have been used or created for this work.

## SDR Data and Homological Perturbation Lemma

Here for reader’s convenience we review the definition of SDR (strong deformation retraction) data and the homological perturbation lemma, both well-known in the literature—see e.g. [GL89, Cra04].

### Definition A.1

Let $(V^\bullet ,d_V)$ and $(W^\bullet ,d_W)$ be a pair of cochain complexes. *SDR data* (or an *(i, p, K) triple*) is a triple of maps

275 $$\begin{aligned} i:W^\bullet \rightarrow V^\bullet ,\quad p:V^\bullet \rightarrow W^\bullet ,\quad K:V^\bullet \rightarrow V^{\bullet -1} \end{aligned}$$

such that:

- *i* and *p* are chain maps: $d_V i=i d_W$, $d_W p=p d_V$.
- *i* is an inclusion and *p* a projection, satisfying $pi=\textrm{id}_W$.
- *K* is a chain homotopy between *ip* and $\textrm{id}_V$: $d_V K+K d_V=\textrm{id}-ip$.
- The following side conditions hold: $K^2=Ki=pK=0$.

In particular, existence of SDR data implies that complexes $(V^\bullet ,d_V)$ and $(W^\bullet ,d_W)$ are quasi-isomorphic (with *i* and *p* quasi-isomorphisms); one calls $W^\bullet$ a *deformation retract* of $V^\bullet$.

An important special case is when $(W^\bullet ,d_W)=(H^\bullet (V),0)$ is the cohomology of *V*.

A choice of SDR data induces a Hodge-like decomposition

276 $$\begin{aligned} V=i(W)\oplus \Big (\textrm{im}(d_V)\cap \textrm{ker}(p)\Big ) \oplus \textrm{im}(K). \end{aligned}$$

Here $d_V$ acts on the first term and maps the third term to the second isomorphically, with *K* the inverse.

### Lemma A.2

(Homological perturbation lemma). Let $(V^\bullet ,d_V)$ and $(W^\bullet ,d_W)$ be a pair of complexes with SDR data (*i*, *p*, *K*). Consider a perturbation of the differential on *V*, $d_V \rightarrow \widetilde{d}_V=d_V+\delta$, for some $\delta :V^\bullet \rightarrow V^{\bullet +1}$ such that $(d_W+\delta )^2=0$. Then the perturbed complex $(V^\bullet ,d_V+\delta )$ is quasi-isomorphic to $(W^\bullet ,\widetilde{d}_W )$ with SDR data $(\widetilde{i},\widetilde{p},\widetilde{K})$, where

277 $$\begin{aligned} \widetilde{d}_W= &  d_W+p\delta i-p\delta K \delta i + p\delta K\delta K \delta i-\cdots , \end{aligned}$$

278 $$\begin{aligned} \widetilde{i}= &  i-K\delta i+K\delta K\delta i-\cdots , \end{aligned}$$

279 $$\begin{aligned} \widetilde{p}= &  p-p\delta K + p\delta K\delta K - \cdots , \end{aligned}$$

280 $$\begin{aligned} \widetilde{K}= &  K-K\delta K+K\delta K\delta K-\cdots , \end{aligned}$$

under the assumption that the geometric progressions above converge.

### First-order deformations of SDR data

Consider a deformation retraction of a cochain complex $(V^\bullet ,d_V)$ onto its cohomology $(W^\bullet =H^\bullet (V),0)$ and fix SDR data (*i*, *p*, *K*). The Hodge-like decomposition (276) in this case is $V=i(W)\oplus V_{d\mathrm {-exact}}\oplus V_{K\mathrm {-exact}}$.

#### Lemma A.3

69 A general infinitesimal deformation of (*i*, *p*, *K*), in the class of SDR data where $p|_{V_{d\mathrm {-closed}}}$ is the standard projection of closed elements to cohomology classes, has the form

281 $$\begin{aligned} i\rightarrow &  i-\varepsilon d_V {\textsf{I}}, \end{aligned}$$

282 $$\begin{aligned} p\rightarrow &  p- \varepsilon {\textsf{P}} d_V, \end{aligned}$$

283 $$\begin{aligned} K\rightarrow &  K+\varepsilon ([d_V,\Lambda ] + i{\textsf{P}} +{\textsf{I}} p), \end{aligned}$$

with ${\textsf{I}},{\textsf{P}},\Lambda$ arbitrary maps

284 $$\begin{aligned} {\textsf{I}}:W^\bullet\rightarrow &  V^{\bullet -1}_{K\mathrm {-exact}}, \end{aligned}$$

285 $$\begin{aligned} {\textsf{P}}:V^\bullet _{d\mathrm {-exact}}\rightarrow &  W^{\bullet -1}, \end{aligned}$$

286 $$\begin{aligned} \Lambda :V^\bullet _{d\mathrm {-exact}}\rightarrow &  V^{\bullet -2}_{K\mathrm {-exact}} \end{aligned}$$

and ε the deformation parameter.

For the applications of this paper, we parametrize the maps ${\textsf{I}},{\textsf{P}}$ above as

287 $$\begin{aligned} {\textsf{I}}={\mathbb {I}}i,\quad {\textsf{P}}=p{\mathbb {P}}, \end{aligned}$$

with

288 $$\begin{aligned} {\mathbb {I}}:V^\bullet \rightarrow V^{\bullet -1}_{K\mathrm {-exact}}, \quad {\mathbb {P}}:V^\bullet _{d\mathrm {-exact}}\rightarrow V^{\bullet -1} \end{aligned}$$

arbitrary maps.

## Variation of Desynchronized Hodge SDR Data

In this section we consider the variation of the SDR data $$(i_{A,A'},p_{A,A'},K_{A,A'})$$ given by the Hodge decomposition associated to a pair of close flat connections $\left(A,A^{\prime }\right)$ and the metric *g*, in the direction of the three parameters $\left(A,A^{\prime },g\right)$.

Recall that the the metric induces on the complex of ${\mathfrak {g}}$-valued differential forms the pairing

289 $$\begin{aligned} \langle \alpha , \beta \rangle _{\Omega ^\bullet (M,{\mathfrak {g}})} = \int _M \langle \alpha , *\beta \rangle _{\mathfrak {g}}\end{aligned}$$

and associated with it the operator $d^*_{A'}$, the formal adjoint of $d_{A'}$, the twisted, desynchronized Hodge-de Rham Laplacian $\Delta _{A,A'}:= (d_{A} + d^*_{A'})^2$, the projection $P_{A,A'}$ to $\ker \Delta _{A,A'}$ along $\operatorname {im} \Delta _{A,A'}$, and the Green’s operator of the Hodge-de Rham Laplacian, $G_{A,A'} = (\Delta _{A,A'} + P_{A,A'})^{-1}$, satisfying $\Delta _{A,A}G_{A,A'} = G_{A,A'}\Delta _{A,A'} = \textrm{id} - P_{A,A'}$. Recall that the SDR data $(i_{A,A'},p_{A,A'},K_{A,A'})$ specified by the Hodge decomposition of the twisted de Rham complex is given by

290 $$\begin{aligned}&i_{A,A'} :H^\bullet _A \rightarrow \Omega ^\bullet ,&i_{A,A'}[\alpha ] = P_{A,A'}\alpha \end{aligned}$$

291 $$\begin{aligned}&p_{A,A'}:\Omega ^\bullet \rightarrow H^\bullet _{A},&p_{A,A'}\beta = \left[ P_{A,A'}\beta \right] \end{aligned}$$

292 $$\begin{aligned}&K_{A,A'} :\Omega ^\bullet \rightarrow \Omega ^{\bullet -1},&K_{A,A'}= d^*_{A,A'} G_{A,A'} \end{aligned}$$

for α a $d_{A}$-closed form and β any ${\mathfrak {g}}$-valued form.

### Changing the kinetic operator

#### Lemma B.1

(Changing the kinetic operator). Let $A_t:(-\epsilon ,\epsilon ) \rightarrow \Omega ^1(M,{\mathfrak {g}})$ a path of smooth flat connections such that $(A_t,A')$ is close for all *t* and $A_0 = A$. Denote $\dot{A_0} = \alpha \in \Omega ^1_\textrm{cl}(M,{\mathfrak {g}})$. Then we have

293

294

295

#### Proof

Since $d_{A_t} = d + \textrm{ad}_{A_t}$ , we have . Equation (293) then follows directly from rewriting the Laplacian as $\Delta _{A,A'} = \left\{ d^*_{A'},d_{A}\right\}$.

To prove Eq. (294), we differentiate the equations $\Delta _{A_t,A'}P_{A_t,A'} = P_{A_t,A'}\Delta _{A_t,A'} = P^2_{A_t,A'}-P_{A_t,A'} =0$. Differentiating the first one we obtain

$$\begin{aligned} \Delta _{A,A'}{\dot{P}}_{A,A'} + {\dot{\Delta }}_{A,A'}P_{A,A'} = 0, \end{aligned}$$

which yields, after composing with $G_{A,A'}$,

296 $$\begin{aligned} (\textrm{id} - P_{A,A'}){\dot{P}}_{A,A'}&= - G_{A,A'} {\dot{\Delta }}_{A,A'}P_{A,A'}&\nonumber \\&= -G_{A,A'}\left( \left\{ d^*_{A'},\textrm{ad}_\alpha \right\} \right) P_{A,A'}&\text {(using 293)} \nonumber \\&= -G_{A,A'}d^*_{A,A'}\textrm{ad}_{\alpha }P_{A,A'}&\text {(since } d^*_{A'}P_{A,A'} = 0) \nonumber \\&= - K_{A,A'}\textrm{ad}_{\alpha }P_{A,A'}&\text {(since } d^*_{A'} \text { commutes with } G_{A,A'}). \end{aligned}$$

Similarly, differentiating $P_{A_t,A'}\Delta _{A_t,A'} = 0$ we obtain

297 $$\begin{aligned} {\dot{P}}_{A,A'}(\textrm{id} - P_{A,A'}) = - P_{A,A'}\textrm{ad}_{\alpha }K_{A,A'}. \end{aligned}$$

Differentiating $P^2_{A_t,A'} - P_{A_t,A'} = 0$ we obtain

$$\begin{aligned} P_{A,A'}{\dot{P}}_{A,A'} + {\dot{P}}_{A,A'}P_{A,A'} - {\dot{P}}_{A,A'} = 0 \end{aligned}$$

or

298 $$\begin{aligned} P_{A,A'}{\dot{P}}_{A,A'} = {\dot{P}}_{A,A'}(\textrm{id} - P_{A,A'}) . \end{aligned}$$

Finally, we can compute, using (296),(297), (298)

$${\dot{P}}_{A,A'} = (\textrm{id} - P_{A,A'}){\dot{P}}_{A,A'} + P_{A,A'}{\dot{P}}_{A,A'} = -\left( K_{A,A'}\textrm{ad}_\alpha \right) P_{A,A'} - P_{A,A'}\left( \textrm{ad}_\alpha K_{A,A'} \right) $$

which proves equation (294).

Finally, let us prove (295). Remember that we have $K_{A_t,A'} = d^*_{A'} G_{A_t,A'}$ and hence

On the other hand, using that $G_{A_t,A'} = (\Delta _{A_t,A'} + P_{A_t,A'})^{-1}$, we have

Using (293) and (294) we obtain

299 $$\begin{aligned} {\dot{G}}_{A,A'} = - G_{A,A'}\big ( \left\{ d^*_{A'},\textrm{ad}_\alpha \right\} - K_{A,A'} \textrm{ad}_\alpha P_{A,A'} - P_{A ,A'} \textrm{ad}_\alpha K_{A,A'} \big )G_{A,A'}. \end{aligned}$$

After applying $d^*_{A'}$, only the first term survives and yields

$$\begin{aligned} {\dot{K}}_{A,A'} = d^*_{A'}{\dot{G}}_{A,A'}-d^*_{A'}G_{A,A'} \left\{ d^*_{A,A'},\textrm{ad}_\alpha \right\} G_{A,A'} = - K_{A,A'}\textrm{ad}_\alpha K_{A,A'} \end{aligned}$$

since $\left( d^*_{A'}\right) ^2 = d^*_{A'}P_{A,A'} = 0$ and $d^*_{A'}$ and $G_{A,A'}$ commute. □

### Changing the gauge-fixing operator

#### Proposition B.2

(Changing the gauge-fixing operator). Let $A_t':(-\epsilon ,\epsilon ) \rightarrow \Omega ^1(M,{\mathfrak {g}})$ a path of smooth flat connections with $A'_0 = A'$ such that $(A,A'_t)$ is flat for all *t*. Denote $\dot{A_0} = \alpha \in \Omega ^1_\textrm{cl}(M,{\mathfrak {g}})$. We denote by $\textrm{ad}_\alpha ^*$ the formal adjoint of $\textrm{ad}_\alpha$ and $K^*_{A,A'}= d_{A} G_{A,A'}$. Then we have

300

301

302

#### Proof

Again, (300) follows directly from writing the Laplacian as $\Delta _{A_t} =\left\{ d_A,d^*_{A'_t}\right\}$. In exactly the same way as above, we then obtain

$$\begin{aligned} {\dot{P}}_{A,A'} = (\textrm{id} - P_{A,A'}){\dot{P}}_{A,A'} + {\dot{P}}_{A,A'}(\textrm{id}-P_{A,A'}) = - K^*_{A,A'} \textrm{ad}^*_\alpha P_{A,A'} - P_{A,A'} \textrm{ad}^*_\alpha K^*_{A,A'}, \end{aligned}$$

proving (301). Using (300), (301), we obtain

303 $$\begin{aligned} {\dot{G}}_{A,A'} = -G_{A,A'}\big ( \left\{ d_{A'},\textrm{ad}^*_\alpha \right\} - K^*_{A,A'} \textrm{ad}^*_\alpha P_{A,A'} - P_{A ,A'} \textrm{ad}^*_\alpha K^*_{A,A'} \big )G_{A,A'}. \end{aligned}$$

After applying $d^*_{A'}$, the third term vanishes. The first one is

$$\begin{aligned}&d^*_{A'}G_{A,A'}\left\{ d_{A},\textrm{ad}^*_\alpha \right\} G_{A,A'} = d^*_{A'}G_{A,A'}d_{A}\textrm{ad}^*_\alpha G_{A,A'} + K_{A,A'}\textrm{ad}^*_\alpha G_{A,A'} d_{A} \\&\quad = (\textrm{id} - P_{A,A'} - d_{A,A'}d^*_{A,A'}G_{A,A'})\textrm{ad}^*_\alpha G_{A,A'} + K_{A,A'}\textrm{ad}^*_\alpha G_{A,A'}d_{A,A'}\\&\quad = \textrm{ad}^*_\alpha G_{A,A'} - P_{A,A'}\textrm{ad}^*_\alpha G_{A,A'} - \left[ d_{A,A'},K_{A,A'}\textrm{ad}^*_\alpha G_{A,A'}\right] . \end{aligned}$$

The second one is

$$\begin{aligned}&d_{A'}^*G_{A,A'} K^*_{A,A'} \textrm{ad}^*_\alpha P_{A,A'} G_{A,A'} = (\textrm{id} - P_{A,A'} - d_{A}d_{A'}^*) G_{A,A'}\textrm{ad}^*_\alpha P_{A,A'}G_{A,A'} \\&\quad = G_{A,A'}\textrm{ad}^*_\alpha P_{A,A'} - P_{A,A'} \textrm{ad}^*_\alpha P_{A,A'} - d_{A}d^*_{A'}G_{A,A'}\textrm{ad}^*_\alpha P_{A,A'} \end{aligned}$$

where we have used that $P_{A,A'}G_{A,A'} = P_{A,A'}$. Using Lemma B.3 below, the compositions $d^*_{A'}\textrm{ad}^*_\alpha P_{A,A'} = P_{A,A'}\textrm{ad}^*_\alpha P_{A,A'} 0$.

The variation of $K_{A,A'}$ is finally given by

$$\begin{aligned} {\dot{K}}_{A,A'}&= \textrm{ad}^*_\alpha G_{A,A'} + d^*_{A'}{\dot{G}}_{A,A'} \\&\quad = P_{A,A'} \textrm{ad}^*_\alpha G_{A,A'} + \left[ d_{A,A'},K_{A,A'}\textrm{ad}^*_\alpha G_{A,A'}\right] + G_{A,A'}\textrm{ad}^*_\alpha P_{A,A'} \end{aligned}$$

proving (302). □

#### Lemma B.3

Let $A_0$ be a flat connection and α $d_{A_0}$-closed 1-form. Then

- The map $\textrm{ad}_\alpha$ maps $d_{A_0}$-closed forms to $d_{A_0}$-closed forms.
- If $[A_0]$ defines a smooth point in the moduli space, $\textrm{ad}_\alpha$ maps all $d_{A_0}$-closed forms to $d_{A_0}$-*exact* forms.
- Dually, $$\textrm{ad}^*_\alpha $$ maps $$d^*_{A_0}$$-closed form to $$d^*_{A_0}$$-closed forms, and $$d^*_{A_0}$$-exact forms if $$[A_0] $$ is a smooth point.

#### Proof

The first point is obvious since the bracket is compatible with the differential. For the second point, notice that $$\textrm{ad}_\alpha $$ always maps exact forms to exact forms. The smoothness assumption implies that for any close $A^{\prime }$, $$l_2([\alpha ],\bullet ) = p_{A_0,A'}\alpha $$ vanishes on harmonic forms, hence $$\textrm{ad}_\alpha $$ maps harmonic forms into exact forms. To prove the last point, let β be a coclosed form and γ an exact form. Then

$$\begin{aligned} \langle \textrm{ad}^*_\alpha \beta , \gamma \rangle = \langle \beta ,\textrm{ad}_\alpha \gamma \rangle = 0 \end{aligned}$$

since coclosed forms are orthogonal to exact forms. Hence $\textrm{ad}^*_\alpha \beta$ is also coclosed. If $[A_0]$ is smooth we can let γ be any closed form, hence $\langle \textrm{ad}_\alpha ^*\beta \rangle$ is coexact in this case. □

We are also interested in the variations of $i_{A_t,A'}$ and $p_{A_t,A'}$. However, remember that $i_{A_t,A'} :H_{A_t}(M,{\mathfrak {g}}) \rightarrow \Omega ^\bullet (M,{\mathfrak {g}})$, so all the $i_{A_t,A'}$ are defined a priori on different spaces. Notice that we have the maps

304

Using these maps to compare the different $i_{A_t,A'}$ and $p_{A_t,A'}$, we have the following result:

#### Lemma B.4

With notation as in Lemma B.1, we have

305

306

#### Proof

Notice that $i_{A_t,A'}p_{A_t,A'} = P_{A_t,A'}$. Then the formulae follow immediately from equation (294). □

In general, the maps (304) are neither injective nor surjective. However, at smooth points, the following is true.

#### Proposition B.5

Suppose $[A_0]$ is smooth. Then for small *t*, the maps (304) are isomorphisms.

#### Proof

It is sufficient to show that for small *t* the restriction of $P_{A_t}$ to $A_0$-harmonic forms is an isomorphism. This follows from Proposition 2.20. □

We remark that the maps (304) coincide with the cohomology comparison maps ${\mathfrak {B}}_{A_t\leftarrow A_0,A'}$, ${\mathfrak {B}}_{A_0\leftarrow A_t,A'}$, cf. Sect. 2.5.4. This follows from comparing (305) with the connection $\nabla ^\textrm{Harm}$ (86).

#### Remark B.6

The formulae (294), (301) for $\frac{d}{dt}\Big |_{t=0}P_{A_t,A'_t}$ can also be obtained as follows: One has

307 $$\begin{aligned} P=\lim _{T\rightarrow \infty } e^{-T\Delta _{A_t,A'_t}}, \end{aligned}$$

hence

308 $$\begin{aligned} {\dot{P}}= \lim _{T\rightarrow \infty } \int _0^T dt\, e^{-t \Delta } (-{{\dot{\Delta }}}) e^{-(T-t)\Delta } . \end{aligned}$$

The $T\rightarrow \infty$ asymptotics of the integral in the r.h.s. comes from two regions (Fig. 14) (a) t≪T, (b) T-t≪T—neighborhoods of the endpoints of the integration interval [0, *T*] (the bulk of the interval does not contribute since $P{{\dot{\Delta }}} P=0$):

309 $$\begin{aligned} \dot{P} =\left( \int _0^\infty dt\, e^{-t\Delta }\right) (-{{\dot{\Delta }}}) e^{-\infty \cdot \Delta }+e^{-\infty \cdot \Delta }(-{{\dot{\Delta }}})\left( \int _0^\infty dt\, e^{-t\Delta }\right) =-G {{\dot{\Delta }}} P- P {{\dot{\Delta }}} G . \end{aligned}$$

Here we are suppressing the subscripts $A,A^{\prime }$ for *P*, Δ, *G*; $$e^{-\infty \cdot \Delta }$$ is a shorthand for the r.h.s. of (307).

### Metric dependence

Let $g_t, t \in (-\epsilon ,\epsilon )$ be a smooth 1-parameter family of Riemannian metrics and denote ${\dot{g}} = \frac{d}{dt}\big |_{t=0}g_t \in \Gamma (\operatorname {Sym}^2(T^*M))$. By a partial contraction with $g^{-1} \in \operatorname {Sym}^2(TM)$ we obtain a endomorphism of the tangent bundle, i.e. a vector-field valued 1-form

310 $$\begin{aligned} \mu = g^{-1}{\dot{g}}\in \Gamma (\textrm{End}(TM)) \cong \Gamma (TM \otimes T^*M) = \Omega ^1(M,TM). \end{aligned}$$

#### Lemma B.7

Denote $$\lambda = \star ^{-1} {\dot{\star }} :\Omega ^p(M) \rightarrow \Omega ^p(M)$$, then

311 $$\begin{aligned} \lambda = \frac{1}{2}\operatorname {tr}\mu - \iota _\mu . \end{aligned}$$

#### Proof

Straightforward computation in local coordinates. □

It is well known (e.g. [RS71]) that we have

312 $$\begin{aligned} {\dot{d}}^*_{A'} = [d^*_{A'},\lambda ].\end{aligned}$$

Analogously to Proposition B.2, we then have the following statements:

#### Proposition B.8

Let $$g_t$$ be a smooth family of Riemannian metrics on *M*, and $$\lambda = \star ^{-1} {\dot{\star }}$$ as above, extended to act on Lie-algebra valued differential forms by tensoring with the identity on $${\mathfrak {g}}$$. Also, let $\left(A,A^{\prime }\right)$ be a pair of close flat connections on *M*. Then, we have

313 $$\begin{aligned} {\dot{\Delta }}_{A,A'}&= [d^*_{A'},\lambda ]d_A + d_A[d^*_{A'},\lambda ], \end{aligned}$$

314 $$\begin{aligned} {\dot{P}}_{A,A'}&= - [d_A, K_{A,A'}\lambda P_{A,A'} - P_{A,A'}\lambda K_{A,A'}], \end{aligned}$$

315 $$\begin{aligned} {\dot{K}}_{A,A'}&= -[d_A,K_{A,A'}\lambda K_{A,A'}] - P_{A,A'}\lambda K_{A,A'} + K_{A,A'} \lambda P_{A,A'}. \end{aligned}$$

#### Proof

Equation (313) follows immediately from (312) To prove (314), we proceed as above in noticing that

316 $$\begin{aligned} {\dot{P}}_{A,A'} = (\textrm{id}- P_{A,A'}){\dot{P}}_{A,A'} + P_{A,A'}{\dot{P}}_{A,A'} = - G_{A,A'}{\dot{\Delta }}_{A,A'} P_{A,A'} - P_{A,A'}{\dot{\Delta }}_{A,A'}G_{A,A'}. \end{aligned}$$

By using (313), we obtain

317 $$\begin{aligned} {\dot{P}}_{A,A'} = - G_{A,A'} d_Ad^*_{A'}\lambda P_{A,A'} + P_{A,A'} \lambda d^*_{A'}d_AG_{A,A'} = - [d_A, K_{A,A'}\lambda P_{A,A'} - P_{A,A'}\lambda K_{A,A'}] \end{aligned}$$

where have also used that $d_A$ and $d^*_{A'}$ commute with $G_{A,A'}$ and annihilate $P_{A,A'}$. Finally, for the variation of $K_{A,A'}$ (315) we obtain

318 $$\begin{aligned} {\dot{K}}_{A,A'} = \underbrace{{\dot{d}}^*_{A,A'}G_{A,A'}}_{=:I} - \underbrace{d^*_{A'}G_{A,A'}{\dot{\Delta }}_{A,A'}G_{A,A'}}_{=:II} -\underbrace{d^*_{A'}G_{A,A'}{\dot{P}}_{A,A'}G_{A,A'}}_{=:III} = I - II - III. \end{aligned}$$

Let us look at the three terms separately. From (312), we get

319 $$\begin{aligned} I = [d^*_{A'},\lambda ] G_{A_{0}}. \end{aligned}$$

For the second term, we obtain

320 $$\begin{aligned}&II = d^*_{A'}G_{A,A'} ([d^*_{A'},\lambda ]d_A + d_A[d^*_{A'},\lambda ]) G_{A,A'} \nonumber \\&\quad = -K_{A,A'}\lambda K_{A,A'}d_A + d^*_{A'}d_AG_{A,A'}[d^*_{A'},\lambda ]G_{A,A'} . \end{aligned}$$

Notice that $\Delta _{A,A'}G_{A,A'} = \textrm{id} - P_{A,A'}$ implies $d^*_{A'}d_AG_{A,A'} = - d_Ad^*_{A'}G_{A,A'} + \textrm{id} - P_{A,A'}$ and therefore

$$\begin{aligned} d^*_{A'}d_AG_{A,A'}[d^*_{A'},\lambda ]G_{A,A'} = d_AK_{A,A'}\lambda K_{A,A'} + [d^*_{A'},\lambda ]G_{A,A'} + P_{A,A'}\lambda K_{A,A'}, \end{aligned}$$

so that

321 $$\begin{aligned} II= [d_A,K_{A,A'}\lambda K_{A,A'}] + [d^*_{A'},\lambda ]G_{A,A'} + P_{A,A'}\lambda K_{A,A'}. \end{aligned}$$

Finally, by using (317) we can rewrite *III* as

322 $$\begin{aligned} III = - K_{A,A'}d_AK_{A,A'}\lambda P_{A,A'} = -K_{A,A'}\lambda P_{A,A'} \end{aligned}$$

since, suppressing indices, $KdK = K (Kd + \textrm{id} - P) = K$ by $K^2 = KP = 0$. Now (315) follows from (318) by using (319),(320),(322). □

## Construction of Extended (*i*, *p*, *K*) Triples From Families

One can obtain formulae (154) and Lemma 4.14 from homological perturbation theory, as follows. Suppose $Q_q$ is a good gauge fixing operator for $d_A$ for $q \in \mathbb{G}\mathbb{F}$, a smooth (but possibly infinite-dimensional) manifold. For fixed $q \in \mathbb{G}\mathbb{F}$, one has the SDR data $(i_q,p_q,K_q)$ from (80). These assemble into SDR data $({\overline{i}},{\overline{p}},{\overline{K}})$ for $d_A$, considered as a differential on $\Omega ^\bullet (M\times \mathbb{G}\mathbb{F},{\mathfrak {g}})$:

323 $$\begin{aligned} \begin{aligned} {\overline{K}}:\Omega ^\bullet (M \times \mathbb{G}\mathbb{F},{\mathfrak {g}};d_A)&\rightarrow \Omega ^\bullet (M \times \mathbb{G}\mathbb{F},{\mathfrak {g}};d_A), \\ {\overline{i}}:\Omega ^\bullet (\mathbb{G}\mathbb{F},H_A(M,{\mathfrak {g}}))&\rightarrow \Omega ^\bullet (M \times \mathbb{G}\mathbb{F},{\mathfrak {g}};d_A), \\ {\overline{p}}:\Omega ^\bullet (M \times \mathbb{G}\mathbb{F},{\mathfrak {g}};d_A)&\rightarrow \Omega ^\bullet (\mathbb{G}\mathbb{F},H_A(M,{\mathfrak {g}})) . \end{aligned} \end{aligned}$$

Similarly, $Q_q$ and the Green’s function $G_q$ assemble into operators ${\overline{Q}},{\overline{G}}$ on $\Omega ^\bullet (M\times \mathbb{G}\mathbb{F},{\mathfrak {g}})$.

We can now deform the differential $d_A$ to the (twisted) de Rham differential on $M \times \mathbb{G}\mathbb{F}$, by the de Rham differential $\delta _q$ in the direction of $\mathbb{G}\mathbb{F}$. Note that since $\delta _q$ increases the de Rham degree in $\mathbb{G}\mathbb{F}$ by 1, the map $1 + \delta _q {\overline{K}}$ is invertible, we denote

324 $$\begin{aligned} X := (1 + \delta _q {\overline{K}})^{-1} \delta _q = \sum _{k\ge 0}(-\delta _q {\overline{K}})^k \delta _q = \delta _q - \delta _q{\overline{K}}\delta _q + \cdots \end{aligned}$$

(this sum is finite since ${\overline{K}}$ decreases the form degree along *M* by 1). We then obtain perturbed SDR data (see Appendix Appendix A)

325 $$\begin{aligned} \begin{aligned} {\widetilde{i}}&= {\overline{i}}- {\overline{K}}X {\overline{i}}= {\overline{i}}- {\widetilde{K}} \delta _q {\overline{i}}, \\ {\widetilde{p}}&= {\overline{p}}- {\overline{p}}X {\overline{K}}= {\overline{p}}- {\overline{p}}\delta _q {\widetilde{K}},\\ {\widetilde{K}}&= {\overline{K}}- {\overline{K}}X {\overline{K}},\\ \widetilde{\delta _q}&= {\overline{p}}X {\overline{i}}= {\overline{p}}\delta _q {\overline{i}}- {\overline{p}}\delta _q {\widetilde{K}} \delta _q {\overline{i}}. \end{aligned} \end{aligned}$$

By Lemma A.2, $({\widetilde{i}},{\widetilde{p}},{\widetilde{K}})$ form SDR data between the complexes $\Omega ^\bullet (M\times \mathbb{G}\mathbb{F},{\mathfrak {g}};d_A + \delta _q)$ and $\Omega ^\bullet (\mathbb{G}\mathbb{F}, H_A(M,{\mathfrak {g}});{\widetilde{\delta }}_q).$

### Proposition C.1

One can rewrite formulae (325) as follows:

326 $$\begin{aligned} \begin{aligned} {\widetilde{i}}&= \sum _{k \ge 0} (-{\overline{G}}(\delta _q{\overline{Q}}))^k {\overline{i}}, \\ {\widetilde{p}}&= \sum _{k \ge 0}{\overline{p}}(-(\delta _q{\overline{Q}}) {\overline{G}})^k, \\ {\widetilde{K}}&= \sum _{k \ge 0} {\overline{K}}(-(\delta _q {\overline{Q}}) {\overline{G}})^k, \\ \widetilde{\delta _q}&= \delta _q + \sum _{k \ge 1} {\overline{p}}(\delta _q{\overline{Q}}) d_A {\overline{G}}(-{\overline{G}}(\delta _q{\overline{Q}}))^k {\overline{i}}. \end{aligned} \end{aligned}$$

Note that, for degree reasons, only k=0,1,2 terms survive in $\widetilde{i},\widetilde{p},\widetilde{K}$ and only k=1 term survives in $\widetilde{\delta }_q$.

In particular, for

327 $$\begin{aligned} \mathbb{G}\mathbb{F} = \{A' \in \textrm{FC}'| (A,A') \text { close}\}, \end{aligned}$$

we have ${\widetilde{i}} = \widehat{i}, {\widetilde{p}} = \widehat{p}, {\widetilde{K}} =\widehat{K}, \widetilde{\delta _q} =\delta _{A'} + \widehat{\Theta }$, with $(\widehat{i},\widehat{p},\widehat{K},\widehat{\Theta })$ given by (154). Lemma 4.14 then follows from the fact that the deformed (*i*, *p*, *K*) triple is again an (*i*, *p*, *K*) triple.

### Proof

One can simplify formulae (325) by noticing that

328 $$\begin{aligned} [\delta _q, {\overline{K}}] = \delta _q{\overline{K}}\in \Omega ^1(\mathbb{G}\mathbb{F},\operatorname {End}(\Omega ^\bullet (M,{\mathfrak {g}}))) \end{aligned}$$

where the right hand side acts as a multiplication operator on differential forms in the $\mathbb{G}\mathbb{F}$ direction. We are using the notations where for an operator $x\in \{{\overline{K}},{\overline{i}},{\overline{p}},{\overline{Q}}\}$, $(\delta _q x)$ stands for $[\delta _q,x]$. By induction, one proves that

329 $$\begin{aligned} {\widetilde{K}} = \sum _{k \ge 0} (-{\overline{K}}\delta _q)^k {\overline{K}}= \sum _{k \ge 0}{\overline{K}}(-\delta _q {\overline{K}})^k = \sum _{k \ge 0} (-\delta _q {\overline{K}})^k {\overline{K}}\end{aligned}$$

where in the second equality we have used that $\delta _q {\overline{K}}$ and ${\overline{K}}$ commute as a consequence of ${\overline{K}}^2=0$. Using further that $({\overline{K}})_q = Q_q G_q$, we have $(\delta _q {\overline{K}})_q = (\delta _q{\overline{Q}})_q G_q - Q_q (\delta _q {\overline{G}})_q$, but using that $K_q Q_q = 0$ we then obtain

330 $$\begin{aligned} {\widetilde{K}}=\sum _{k \ge 0} {\overline{K}}( -(\delta _q {\overline{Q}}) {\overline{G}})^k = \sum _{k \ge 0} {\overline{G}}(- (\delta _q {\overline{Q}}) {\overline{G}})^k {\overline{Q}}. \end{aligned}$$

This proves the third equation in (326) We can then also rewrite the first two equations in (325) by realizing that

331 $$\begin{aligned} {\overline{Q}}\delta _q {\overline{i}}= {\overline{Q}}(\delta _q{\overline{i}}) = (\delta _q {\overline{Q}}) {\overline{i}}\end{aligned}$$

and

332 $$\begin{aligned} {\overline{p}}\delta _q {\overline{Q}}= -(\delta _q{\overline{p}}) {\overline{Q}}= {\overline{p}}(\delta _q{\overline{Q}}), \end{aligned}$$

combining (325), (330),(331) we get

$$\begin{aligned}&{\widetilde{i}} = {\overline{i}}- \sum _{k \ge 0} {\overline{G}}(-(\delta _q{\overline{Q}}) {\overline{G}})^k {\overline{Q}}\delta _q {\overline{i}}\\&\quad = {\overline{i}}+ \sum _{k \ge 0} {\overline{G}}(-(\delta _q{\overline{Q}}) {\overline{G}})^k (-\delta _q{\overline{Q}}) {\overline{i}}= \sum _{k \ge 0} (-{\overline{G}}(\delta _q{\overline{Q}}))^k {\overline{i}}\end{aligned}$$

which proves the first equation in (326). Combining (325), (330) and (332), we obtain

$$\begin{aligned}&{\widetilde{p}} = {\overline{p}}- {\overline{p}}(\delta _q {\overline{Q}}) {\overline{G}}\sum _{k \ge 0} (-(\delta _q{\overline{Q}}) {\overline{G}})^k\\&\quad = {\overline{p}}- {\overline{p}}(\delta _q{\overline{Q}}) {\overline{G}}\sum _{k \ge 0}(-(\delta _q{\overline{Q}}) {\overline{G}})^k = {\overline{p}}\sum _{k \ge 0}(-(\delta _q{\overline{Q}}) {\overline{G}})^k, \end{aligned}$$

which proves the second equation in (326). Finally, we focus on the last equation. The first term is simply

$$\begin{aligned} {\overline{p}}\delta _q {\overline{i}}= \underbrace{{\overline{p}}{\overline{i}}}_{=1_{H^\bullet (M,{\mathfrak {g}})}} \delta _q + \underbrace{{\overline{p}}(\delta _q{\overline{i}})}_{=0} = \delta _q. \end{aligned}$$

For the second term, notice that we have ${\overline{p}}{\widetilde{K}} = 0$ and therefore

$$\begin{aligned} {\overline{p}}\delta _q {\widetilde{K}} \delta _q {\overline{i}}= -(\delta _q {\overline{p}}) {\widetilde{K}} \delta _q {\overline{i}}= (\delta _q{\overline{p}}) \sum _{k\ge 1} (-{\overline{G}}(\delta _q {\widehat{Q}}_0))^k {\overline{i}}. \end{aligned}$$

The proof of the last equation in (326) now follows from

$$\begin{aligned} \delta _q{\overline{p}}= -{\overline{p}}(\delta _q{\overline{Q}}) d_A {\overline{G}}, \end{aligned}$$

which in turn can be proved by deriving the identity ${\overline{p}}{\overline{Q}}= 0$:

$$\begin{aligned} (\delta _q{\overline{p}}){\overline{Q}}= -{\overline{p}}(\delta _q{\overline{Q}}) \end{aligned}$$

and then composing both sides with $d_A{\overline{G}}$ on the left:

$$\begin{aligned} -{\overline{p}}(\delta _q{\overline{Q}}) d_A {\overline{G}}= (\delta _q{\overline{p}}) {\overline{Q}}d_A {\overline{G}}= - (\delta _q{\overline{p}}) (d_A {\overline{Q}}\, {\overline{G}}- \textrm{id} + {\overline{i}}{\overline{p}}) = \delta _q{\overline{p}}, \end{aligned}$$

because $(\delta _q{\overline{p}}) d_A = \delta _q({\overline{p}}d_A) = 0$ and $(\delta _q{\overline{p}}) {\overline{i}}= - {\overline{p}}( \delta _q{\overline{i}}) = 0$, because changing *q* shifts representatives of cohomology by $d_A$-exact terms and ${\overline{p}}d_A=0$. □

## Some Technical Proofs

### Proof of Propositions 2.12 and 2.13

#### Proof of Proposition 2.12

##### Proof

For point (i), notice that because the assumption of boundedness of $K_{A_0}$ in a Banach norm, by the Banach inverse function theorem the inverse exists in a neighborhood of every point δ where the differential of $\widetilde{\kappa }_{A_0}$,

333 $$\begin{aligned} (d \widetilde{\kappa }_{A_0})_\delta = \textrm{Id} + K_{A_0}\textrm{ad}_{\mathfrak {d}}:\Omega ^1 \rightarrow \Omega ^1 \end{aligned}$$

is invertible. In particular, by the triangle inequality this happens when the operator norm of $K_{A_0}{\mathfrak {d}}$ is less than one.

For point ii), we have to show $$\widetilde{\delta }_{A_0}(\widetilde{\kappa }_{A_0}(\alpha )) = \widetilde{\kappa }_{A_0}(\widetilde{\delta }_{A_0}(\alpha ) = \alpha .$$ To see that $$\widetilde{\delta }_{A_0}(\widetilde{\kappa }_{A_0}(\alpha )) = \alpha , $$ recall that the coefficients $$\alpha ^{(j)}$$ of $$\widetilde{\delta }_{A_0}$$ are given by summing over binary trees with *j* leaves, with prefactor $$1/2^{j+1}$$ and sign $$(-1)^{j+1}$$. When evaluating $$\widetilde{\delta }_{A_0}(\widetilde{\kappa }_{A_0}(\alpha )$$ we are placing $$\widetilde{\kappa }_{A_0}(\alpha )$$ instead on every leaf. But since $$\widetilde{\kappa }_{A_0}(\alpha ) = \alpha + \frac{1}{2}K_{A_0}[\alpha ,\alpha ]$$, we can express $$\widetilde{\delta }_{A_0}(\widetilde{\kappa }_{A_0}(\alpha ))$$ again as a sum over binary trees $T^{\prime }$ evaluated according to the same rules, but with a different combinatorial factor $$c_{T'}$$, allowing for the fact that the same tree $T^{\prime }$ could arise from several different trees *T*. See Fig. 15 for an example.

We claim that $$c_{T'} = 0$$ for all trees with at least two leaves. Indeed, for a tree $T^{\prime }$ let $$n_{T'}$$ denote the number of internal vertices connected to exactly two leaves. Note that $$n_{T'} = 0$$ if and only if $T^{\prime }$ is the tree with a single leaf at the root and no internal vertex. If *v* is such an internal vertex, then we call *v* together with the two adjacent leaves a corolla. Then $T^{\prime }$ could be obtained from the tree *T* where we collapse the corolla of *v* into a leaf α. Note that this operation changes the sign. In total, we will obtain the tree $T^{\prime }$ exactly $$2^{n_{T'}}$$ times, but with different signs: If we collapse *k* corollas then there is a sign $$(-1)^k$$. Therefore the combinatorial coefficient of $T^{\prime }$ is $$c_{T'} = \sum _{k \ge 0}^{n_{T'}}(-1)^k{k \atopwithdelims (){n_{T'}}} = 0$$. The other direction $$\widetilde{\kappa }_{A_0}(\widetilde{\delta }_{A_0}(\alpha )) = \alpha $$ is proven similarly.

As for point iii), we simply compute

$$\begin{aligned} d_{A_0}{\widetilde{\kappa }_{A_0}}(\alpha ) = d_{A_0}\alpha + \frac{1}{2} d_{A_0}K_{A_0}[\alpha ,\alpha ] = d_{A_0}\alpha + \frac{1}{2}[\alpha ,\alpha ] - K_{A_0}d_{A_0}[\alpha ,\alpha ] - p_{A_0}[\alpha ,\alpha ]. \end{aligned}$$

If $d_{A_0}\alpha = -\frac{1}{2}[\alpha ,\alpha ]$, then the first two terms cancel and the latter two terms vanish because $[\alpha ,\alpha ] = -2d_{A_0}\alpha$ is exact. Point iv) follows immediately from the definition of $\widetilde{\varphi }_{A_0} = A_0 + \widetilde{\delta }_{A_0}$ and points i) and iii). □

#### Proof of Proposition 2.13

Let $A_1$ be a different flat connection, and $m = A_1 - A_0$. Then $m \in MC_{A_0}$, and therefore $d_{A_0}\widetilde{\kappa }_{A_0}(m)=0$, so it defines a tangent vector to the space of flat connections at $A_0$ (and moreover, if $K_{A_0}m = 0$, then $K_{A_0}^2 = 0$ implies $K_{A_0}\widetilde{\kappa }_{A_0} = 0$). In this case, $A(t) = A_0+\widetilde{\delta }_{A_0}(t \widetilde{\kappa }_{A_0}(m))$ defines a curve of flat connections with $A(1) = A_1$.

##### Lemma D.1

Suppose $A_0$ is a smooth point. Then there are neighborhoods $0 \in U \subset \textrm{Im}\, i_{A_0}$ and $0 \in V \subset MC_{A_0} \cap \ker K_{A_0}$ such that $\widetilde{\delta }_{A_0}:U \rightarrow V$ is bijective with inverse $\widetilde{\kappa }_{A_0}$.

##### Proof

We know that $\widetilde{\delta }_{A_0}$ converges in a neighborhood $U_1$ of $0 \in \Omega ^1_l$, therefore $\widetilde{\delta }_{A_0}$ is defined on $U = U_1 \cap \operatorname {Im}i_{A_0}$. On $\operatorname {Im} i_{A_0}$, we know that $\widetilde{\delta }_{A_0} \in MC_{A_0}$ by Proposition 2.7. Since $\widetilde{\delta }_{A_0}(\alpha ) = \alpha + K_{A_0}(\ldots )$, we have $K_{A_0}\widetilde{\delta }_{A_0}(\alpha ) = 0$. Therefore $\widetilde{\delta }_{A_0}(U) \subset MC_{A_0} \cap \ker K_{A_0}$. On the other hand, if $m \in MC_{A_0} \cap \ker K_{A_0}$, then $d_{A_0}\widetilde{\kappa }(m) = K_{A_0}\widetilde{\kappa }(m) = 0$, i.e $\widetilde{\kappa }_{A_0}(m) \in \operatorname {Im}i_{A_0}$. For *m* small enough, we therefore have $\widetilde{\kappa }_{A_0}m \in U$, and since we already know that $\widetilde{\delta }_{A_0}$ and $\widetilde{\kappa }_{A_0}$ are inverse to each other, we conclude the statement. □

##### Corollary D.2

The restriction of the map $\kappa _{A_0}:\Omega ^1 \rightarrow H^1_{A_0}$ given by

334 $$\begin{aligned} \kappa _{A_0}({\mathfrak {d}}) = p_{A_0}({\mathfrak {d}}+ \frac{1}{2}K_{A_0}[{\mathfrak {d}},{\mathfrak {d}}]) = p_{A_0}({\mathfrak {d}})\in H^1_{A_0} \end{aligned}$$

to the image of $\delta _{A_0}:H^1_{A_0}\supset U \rightarrow {\Omega ^1(M,{\mathfrak {g}})}$, is a compositional inverse to $\delta _{A_0}$.

Finally, let us prove Proposition 2.13.

##### Proof of Proposition 2.13

We begin with point i). To show surjectivity onto a small neighborhood of

$A_0 \in \textrm{FC}_l$, in the first step, we construct a gauge transformation that takes an arbitrary connection $A_1$ close enough to $A_0$ to a connection $A_1'$ satisfying $K_{A_0}(A_1'-A_0)=0$. To this end, consider the map $F :\Omega ^0_{K-\textrm{ex}, l} \times \Omega ^1_l \rightarrow \Omega ^0_{K-\textrm{ex},l}$ given by

$$\begin{aligned} (\beta , {\mathfrak {d}}) \rightarrow K_{A_0}(\, ^{\exp (-\beta )}(A_0 + {\mathfrak {d}}) - A_0 ). \end{aligned}$$

We want to solve for $\beta = \beta ({\mathfrak {d}})$ such that $F(\beta ({\mathfrak {d}}),{\mathfrak {d}}) = 0$, this is the desired gauge transformation. Existence of β, for small enough ${\mathfrak {d}}$, is then guaranteed by the implicit function theorem for Banach spaces, since the derivative of *F* at (0, 0) in direction of β is $(dF/d\beta )(0,0) = K_{A_0}d_{A_0} = \textrm{id}_{\Omega ^0_{K-\textrm{ex}}}$. This means that for $A_1$ close enough to $A_0$, there is β such that $K_{A_0}(\,{}^{\exp (-\beta )}A_1-A_0) = 0$. For such connections, we know that $\widetilde{\kappa }_{A_0}(\,{}^{\exp (-\beta )}A_1-A_0)$ is a $d_{A_0}$- and $K_{A_0}$-closed 1-form. Therefore, if $A_1$ is a flat connection close to $A_0$, then $\alpha = \widetilde{\kappa }_{A_0}(\,{}^{\exp (-\beta )}A_1-A_0) - d_{A_0}\beta$ is a $d_{A_0}$-closed form such that $\widetilde{\psi }_{A_0}(\alpha ) = A_1$.

As for point ii), we notice that the implicit function theorem also guarantees smoothness of the map ${\mathfrak {d}}\mapsto \beta ({\mathfrak {d}})$. It follows that $\widetilde{\psi }_{A_1}^{-1} \circ \widetilde{\psi }_{A_0}$ is a composition of smooth maps between differential forms (projections, gauge transformations, β, and the maps $\widetilde{\varphi }$ and $\widetilde{\kappa }$) and hence smooth. □

### Proof of Proposition 4.4: “horizontality” of Ray-Singer torsion

We first need some auxiliary results.

#### Lemma D.3

Given a path of flat connections $A_t$, one has the following formula for the infinitesimal change of the Ray-Singer torsion $\tau _{A_t}$:

335 $$\begin{aligned} \left. \frac{d}{dt}\right| _{t=0}\det ({\mathfrak {B}}^\textrm{diag}_{A_0\leftarrow A_t})\tau _{A_t}=\tau _{A_0}\,\textrm{Str}_{\Omega ^\bullet }(K_{A_0}\textrm{ad}_{{\dot{A}}_0}). \end{aligned}$$

Here ${\mathfrak {B}}^\textrm{diag}_{A_0\leftarrow A_t}:H_{A_t}\rightarrow H_{A_0}$ is the projection to cohomology of the parallel transport of the connection $\nabla ^\textrm{Harm}$ along the path $(A_{t-\tau },A_{t-\tau })$, $0\le \tau \le t$ in $\textrm{FC}'\times \textrm{FC}'$.

#### Proof

By definition of Ray-Singer torsion,

336 $$\begin{aligned} \tau _{A_t}=\mu _{A_t} \prod _{p=0}^3 \left( {\det }'_{\Omega ^p} \Delta _{A_t}\right) ^{\frac{-(-1)^pp}{2}} \end{aligned}$$

with $\mu _{A_t}$ the volume element in $\textrm{Det}H^\bullet _{A_t}$ corresponding to Hodge inner product. Note that in this lemma we are using the synchronized $(A_t,A_t)$ Hodge decompositon. Since ${\mathfrak {B}}_{A_t\leftarrow A_0}^\textrm{diag}$ is an isometry (Proposition 2.38 (b)), we have $\det ({\mathfrak {B}}_{A_t\leftarrow A_0}) \mu _{A_t}=\mu _{A_0}$. Hence,

337 $$\begin{aligned} &  \tau _{A_0}^{-1}\left. \frac{d}{dt}\right| _{t=0}\det ({\mathfrak {B}}_{A_0\leftarrow A_t})\tau _{A_t}= \left. \frac{d}{dt}\right| _{t=0} \log \prod _{p=0}^3 \left( {\det }'_{\Omega ^p} \Delta _{A_t}\right) ^{\frac{-(-1)^pp}{2}} \nonumber \\ &  \quad = \sum _{p=0}^3\frac{-(-1)^p p}{2} \textrm{tr}_{\Omega ^p} ({\dot{\Delta }} G), \end{aligned}$$

where

$${\dot{\Delta }}:=\frac{d}{dt}\Big |_{t=0}\Delta _{A_t}=[\textrm{ad}_{{\dot{A}}_0},d^*]_+ + [d,\textrm{ad}^*_{{\dot{A}}_0}]_+ .$$

Here we suppress the subscript $A_0$ in $G,d,d^*$. Continuing the computation (337) we have

$$\begin{aligned} &  \cdots = \sum _{p=0}^3 \frac{-(-1)^p p}{2}\Big (\operatorname {tr}_{\Omega ^{p-1}} \underbrace{d^*G}_K \textrm{ad}_{{\dot{A}}_0} +\operatorname {tr}_{\Omega ^{p}} \underbrace{d^*G}_K \textrm{ad}_{{\dot{A}}_0} +\operatorname {tr}_{\Omega ^p} \underbrace{dG}_{K^*} \textrm{ad}^*_{{\dot{A}}_0} + \operatorname {tr}_{\Omega ^{p+1}} \underbrace{dG}_{K^*} \textrm{ad}^*_{{\dot{A}}_0} \Big )\\ &  \quad = \sum _{p=0}^3 \underbrace{\frac{-(-1)^p p-(-1)^{p+1}(p+1)}{2}}_{\frac{(-1)^p}{2}} \operatorname {tr}_{\Omega ^p} K \textrm{ad}_{{\dot{A}}_0} + \underbrace{\frac{-(-1)^p p-(-1)^{p-1}(p-1)}{2}}_{\frac{-(-1)^p}{2}} \operatorname {tr}_{\Omega ^p} K^* \textrm{ad}^*_{{\dot{A}}_0} \\ &  \quad =\frac{1}{2} \textrm{Str}_{\Omega ^\bullet } K\textrm{ad}_{{\dot{A}}_0}- \frac{1}{2} \textrm{Str}_{\Omega ^\bullet } \underbrace{K^*\textrm{ad}^*_{{\dot{A}}_0}}_{*K\textrm{ad}_{{\dot{A}}_0}*} = \textrm{Str}_{\Omega ^\bullet } K\textrm{ad}_{{\dot{A}}_0}. \end{aligned}$$

This proves (335). □

#### Remark D.4

Traces in the proof above should be understood as zeta-regularized traces. For instance, $\textrm{Str}_{\Omega ^\bullet }K\textrm{ad}_{{\dot{A}}_0}$ should be understood as

338 $$\begin{aligned} \textrm{Str}_{\Omega ^\bullet }K\textrm{ad}_{{\dot{A}}_0}:=\lim _{s\rightarrow 0} \int _0^\infty du\, u^s\, \textrm{Str}_{\Omega ^\bullet } d^* e^{-u \Delta _{A_0}} \textrm{ad}_{{\dot{A}}_0}. \end{aligned}$$

However, by the results of Axelrod-Singer [AS91], the singular terms of the heat kernel expansion are proportional to $\textrm{id}\in \textrm{End}({\mathfrak {g}})$ and hence vanish under the trace with $\textrm{ad}_{{\dot{A}}_0}$ by unimodularity of ${\mathfrak {g}}$. Therefore, the zeta-regularized supertrace coincides with the point-splitting regularized supertrace that we use to define tadpoles in Feynman diagrams, cf. footnote 45.

#### Lemma D.5

Given a path of flat connections $$A_t$$ and $A^{\prime }$ close $$A_0$$, we have

339 $$\begin{aligned} \left. \frac{d}{dt}\right| _{t=0} \det ({\mathfrak {B}}_{A_0\leftarrow A_t;A'})\tau _{A_t} = \tau _{A_0} \,\textrm{Str}_{\Omega ^\bullet } (K_{A_0,A'} \textrm{ad}_{{\dot{A}}_0}). \end{aligned}$$

#### Proof

Consider a path $A'_s$ in $\textrm{FC}'$ starting at $A'_0=A_0$ and ending at $A'_1=A'$ (and staying close to $A_0$). Denote

$$ f_s:= \tau _{A_0}^{-1}\left. \frac{d}{dt}\right| _{t=0} \det ({\mathfrak {B}}_{A_0\leftarrow A_t;A'_s})\tau _{A_t},\quad h_s:= \textrm{Str}_{\Omega ^\bullet } (K_{A_0,A'_s} \textrm{ad}_{{\dot{A}}_0}). $$

Note that Lemma D.3 implies that $f_0=h_0$. To prove the result it suffices to show that $\frac{d}{ds} f_s=\frac{d}{ds} h_s$. For the derivative of $h_s$ we find

340

For $f_s$ we have

341 $$\begin{aligned} &  f_s=\tau _{A_0}^{-1}\left. \frac{d}{dt}\right| _{t=0} \det (\textrm{Hol}_{\nabla ^\textrm{Harm}}(R_{s,t}))\cdot \det ({\mathfrak {B}}_{A_0\leftarrow A_t;A_0}) \tau _{A_t} \nonumber \\ &  \quad = f_0+\left. \frac{d}{dt}\right| _{t=0}\det \textrm{Hol}_{\nabla ^\textrm{Harm}}(R_{s,t}). \end{aligned}$$

Here $R_{s,t}$ is the (curved) rectangle in $\mathcal {U}$ with sides (i) $(A_\tau ,A_0)$ with 0<τ<t, (ii) $(A_t,A'_\sigma )$ with 0<σ<s, (iii) $(A_{t-\tau },A'_s)$ with 0<τ<t, (iv) $(A_0,A'_{s-\sigma })$ with 0<σ<s; $\textrm{Hol}_{\nabla ^\textrm{Harm}}(R_{s,t})\in \textrm{End}(\textrm{Harm}_{A_0,A_0})$ stands for the holonomy of $\nabla ^\textrm{Harm}$ around the rectangle.

Denote $\rho _{s,t,\epsilon }=R_{s+\epsilon ,t}-R_{s,t}$ (here difference is an operation on singular 1-chains)—a small rectangle with vertices at $(A_0,A'_s)$, $(A_t,A'_s)$, $(A_t,A'_{s+\epsilon })$, $(A_0,A'_{s+\epsilon })$. Next, (341) implies

342 $$\begin{aligned} &  \frac{d}{ds}f_s= \left. \frac{\partial ^2}{\partial \epsilon \, \partial t}\right| _{\epsilon =t=0} \det \textrm{Hol}_{\nabla ^\textrm{Harm}}(\rho _{s,t,\epsilon }) \nonumber \\ &  \quad =-\textrm{Str}\, \iota _{\partial _s A'_s}\iota _{{\dot{A}}_0}F_{\nabla ^\textrm{Harm}}\Big |_{(A_0,A'_s)} =\textrm{Str}\,P (\textrm{ad}^*_{\partial _s A'_s} G \textrm{ad}_{{\dot{A}}_0}-\textrm{ad}_{{\dot{A}}_0} G \textrm{ad}^*_{\partial _s A'_s}). \end{aligned}$$

Here in the last step we used the result (89) for the curvature of $\nabla ^\textrm{Harm}$. Comparing with (340), we see that we have $\partial _s f_s=\partial _s h_s$ which, together with the initial condition $f_0=h_0$ implies the desired result $f_1=h_1$. □

#### Proof of Proposition 4.4

Let $A_t=\varphi (A,A',t\alpha )$ —a path of flat connections from *A* at t=0 to $\widetilde{A}$ at t=1. We want to show that

343 $$\begin{aligned} \det ({\mathfrak {B}}_{A\leftarrow A_t;A'})\circ \tau _{A_t} {\mathop {=}\limits ^{!}} \tau _A \exp \sum _\gamma \frac{2}{|\textrm{Aut}(\gamma )|}\Phi _{\gamma ,A,A'}(t\alpha ). \end{aligned}$$

For t=1, this is the desired relation (115). Denote the l.h.s. of (343) by $\lambda _t$ and the r.h.s. by $\mu _t$. We have $\lambda _0=\mu _0$, so it suffices to prove $\lambda _t^{-1}\partial _t \lambda _t=\mu _t^{-1}\partial _t\mu _t$.

We have

344 $$\begin{aligned} &  \frac{d}{dt}\lambda _t=\left. \frac{d}{d\epsilon }\right| _{\epsilon =0} \det {\mathfrak {B}}_{A\leftarrow A_t;A'}\circ (\det {\mathfrak {B}}_{A_t\leftarrow A_{t+\epsilon };A'}\circ \tau _{A_t+\epsilon }) \nonumber \\ &  \quad \underset{\textrm{Lemma}\; {\mathrm{D.5}}}{=} \det {\mathfrak {B}}_{A\leftarrow A_t;A'}\circ \tau _{A_t}\,\textrm{Str}\, K_{A_t,A'}\textrm{ad}_{{\dot{A}}_t}= \lambda _t \,\textrm{Str}\, K_{A_t,A'}\textrm{ad}_{{\dot{A}}_t}. \end{aligned}$$

To analyze $\mu _t$, we first remark that

345 $$\begin{aligned} \exp \sum _\gamma \frac{2}{|\textrm{Aut}(\gamma )|}\Phi _{\gamma ,A,A'}(t\alpha )= \textrm{Sdet}_{\Omega ^\bullet }(1+K_{A,A'}\textrm{ad}_{A_t-A}). \end{aligned}$$

Indeed, log of the r.h.s. here is

$$\begin{aligned} \textrm{Str}\,\log (1+K_{A,A'}\textrm{ad}_{A_t-A})=\sum _{n\ge 1}\frac{-1}{n}\textrm{Str}(-K_{A,A'}\textrm{ad}_{A_t-A})^n \end{aligned}$$

– twice the sum of one-loop graphs, with n≥1 trees plugged into the cycle.

From (345) we find

346 $$\begin{aligned} \frac{d}{dt} \mu _t=\mu _t \textrm{Str} \underbrace{(1+K_{A,A'}\textrm{ad}_{A_t-A})^{-1}K_{A,A'}}_{K_{A_t,A'}}\textrm{ad}_{{\dot{A}}_t}. \end{aligned}$$

Comparing with (344), we see that $\lambda _t^{-1}{{\dot{\lambda }}}_t=\mu _t^{-1}{{\dot{\mu }}}_t$.^70^ This finishes the proof. □

### Vanishing of contributions of hidden boundary strata to $\delta _{A'}Z$ in Proposition 4.11

Let Γ be a trivalent graph, possibly with leaves. Let $$\Gamma ^\textrm{m}$$ be Γ with one edge marked by Λ (instead of *K*) or one leaf marked by $${\mathbb {I}} i({\textsf{a}})$$ (instead of $$i({\textsf{a}})$$). Let $$\omega _{\Gamma ^\textrm{m}}$$ be the corresponding form on the configuration space $$\overline{\textrm{Conf}}_V(M)$$—the integrand of (99) in the $\left(A,A^{\prime }\right)$-gauge, with the factor for the marked edge or leaf replaced by Λ or $${\mathbb {I}}i({\textsf{a}})$$.

Let $\Gamma^{\prime }$ be a full subgraph of $$\Gamma ^\textrm{m}$$ on a subset $V^{\prime }$ of vertices with $|V^{\prime }|\geq 3$ and let $$\partial _{V'} \overline{\textrm{Conf}}_V(M)$$ be the codimension 1 boundary stratum of the compactified configuration space corresponding to vertices $V^{\prime }$ collapsing at a point of *M*, see [AS94].

#### Proposition D.6

The contribution of the hidden boundary stratum $\partial _{V'} \overline{\textrm{Conf}}_V(M)$ to the variation of the partition function $\delta _{A'}Z_{A,A'}({\textsf{a}})$ vanishes:

347 $$\begin{aligned} \int _{\partial _{V'} \overline{\textrm{Conf}}_V(M)} \omega _{\Gamma ^\textrm{m}} =0 \end{aligned}$$

Before the proving the proposition, we need the following lemma.

Let $\eta _\Lambda \in \Omega ^1(\overline{\textrm{Conf}}_2(M),{\mathfrak {g}}\otimes {\mathfrak {g}})$ be the integral kernel of the operator Λ (129).

We will call a form $\alpha \in \Omega ^\bullet (\overline{\textrm{Conf}}_2(M))$ *regular*^71^ if its restriction to the boundary of the configuration space^72^ is the pullback of a smooth form on *M*:

$$\begin{aligned} \alpha |_{\partial \overline{\textrm{Conf}}_2(M)} = (\rho |_\partial )^* \beta \end{aligned}$$

for some $\beta \in \Omega ^1(M,{\mathfrak {g}}\otimes {\mathfrak {g}})$. Here $\rho :\overline{\textrm{Conf}}_2(M)\rightarrow M\times M$ is the blowdown map and $\rho |_\partial :\partial \overline{\textrm{Conf}}_2(M)\rightarrow \textrm{Diag}\cong M$ is its restriction to the boundary ($\textrm{Diag}\subset M\times M$ is the diagonal).

#### Lemma D.7

The form $\eta _\Lambda$ is regular.

#### Proof

The leading singularity of $\eta _\Lambda$ on the diagonal is

348 $$\begin{aligned} \eta _\Lambda (x,y) \sim _{x\rightarrow y} \frac{1}{4\pi }(\det g)^{\frac{1}{2}} g^{\alpha \zeta } f^{abc} \epsilon _{\alpha \beta \gamma } \delta A'^{b}_\zeta \frac{u^\beta }{|| u||} du^\gamma + \cdots \end{aligned}$$

where ⋯ stands for less singular terms; u=x-y (in a local chart at *y*); *g* and $\delta A^{\prime }$ are evaluated at *y*. This result is obtained by Hadamard parametrix method, by performing a model computation of the integral kernel of $$\Lambda =K \textrm{ad}^*_{\delta A'}G$$ on $${\mathbb {R}}^3$$ with $A=A^{\prime }=0$ and constant $\delta A^{\prime }$.^73^ The ansatz (348) immediately implies the statement of the lemma.

□

#### Corollary D.8

The integral kernel $$\eta _{A,A'}$$ of the chain homotopy $$K_{A,A'}$$ considered modulo regular forms on $$\overline{\textrm{Conf}}_2(M)$$ does not depend on $A^{\prime }$. In particular, it coincides with the result of Axelrod-Singer [AS91, PL5] at $A^{\prime }=A$.

#### Proof

By (130), we have $\delta _{A'}\eta _{A,A'}=d_A \eta _\Lambda + \rho ^*(\cdots )$ with ⋯ a smooth form on M×M. Here $d_A$ acts on both arguments of $\eta _\Lambda$. Restricting to $\partial \overline{\textrm{Conf}}_2(M)$ and using Lemma D.7, we get the statement of the corollary. □

Thus, according to the result of [AS91, PL5], the propagator near the diagonal in M×M splits into singular, bounded and regular pieces: $$\eta =\eta ^\textrm{sing}+\eta ^\textrm{bd}+\eta ^\textrm{reg}$$. Note that the singular and bounded pieces do not depend on $A,A^{\prime }$ and are of the form $$\delta ^{ab}$$ times a form on $$\overline{\textrm{Conf}}_2(M)$$ with real coefficients.

#### Proof of Proposition D.6

Consider the integral in the l.h.s. of (347). Consider a decoration of edges of $\Gamma^{\prime }$ by pieces $$\eta ^\textrm{sing}$$, $$\eta ^\textrm{bd}$$, $$\eta ^\textrm{reg}$$. Let $$\overline{\Gamma }'$$ be the subgraph of $\Gamma^{\prime }$ where we only retain edges decorated by $$\eta ^\textrm{sing}$$ or $$\eta ^\textrm{bd}$$ (edges decorated by $$\eta ^\textrm{reg}$$, the possible edge decorated by $$\eta _\Lambda =\eta _\Lambda ^\textrm{reg}$$, and the leaves are all removed in $${\overline{\Gamma }}'$$). We have the following:

- If ${\bar{\Gamma }}'$ contains a univalent vertex *v*, the integral (347) vanishes by degree reason (one has an integral over 3-dimensional space of positions of *v*, of a form of degree ≤2 in the position of *v*).^74^ Also, if ${\bar{\Gamma }}'$ contains a 0-valent vertex, the integral (347) vanishes by a similar degree reason.
- If ${\bar{\Gamma }}'$ contains a bivalent vertex, the integral vanishes by Kontsevich’s vanishing lemma [Kon94, Lemma 2.2].^75^
- If ${\overline{\Gamma }}'={\overline{\Gamma }}_1\sqcup {\overline{\Gamma }}_2$ is disconnected, the integral (347) vanishes: fixing a point x∈M, the integral over the fiber of $\partial _{V'}\overline{\textrm{Conf}}_V(M)\rightarrow M$ over *x* vanishes due to invariance of the integrand under translation of vertices of ${\overline{\Gamma }}_1$ by any vector $w\in T_xM$.

Starting with a trivalent graph Γ, possibly with leaves, with at least one marked edge or one marked leaf, select a full subgraph $\Gamma^{\prime }$ and construct the associated graph $${\overline{\Gamma }}'$$—in particular the marked edge/leaf is removed in $${\overline{\Gamma }}'$$. The integral (347) vanishes by (a), (b), (c) above unless $${\overline{\Gamma }}'$$ is a connected trivalent subgraph of Γ with all edges decorated by $$\eta ^\textrm{sing}$$ or $$\eta ^\textrm{bd}$$. But the latter integral also vanishes by degree reason: the form degree of the integrand is 1 higher than the dimension of the domain of integration.

□

#### Remark D.9

The proof of vanishing above also works in the case when $\Gamma^{\prime }$ contains two vertices *u*, *v*, with $${\overline{\Gamma }}'$$ containing 2 or 3 edges connecting *u* and *v*.

#### Remark D.10

The proof above is an application of technology of [AS94, CM08, Appendix A]. These references consider the contribution of hidden strata of the configuration space on the variation of Chern–Simons partition function with respect to a change of metric. In that case one actually does get an anomaly (the framing anomaly) from the strata corresponding to the collapse of all vertices of a connected component (with no leaves) of a graph. In the case of variation of $A^{\prime }$ such an anomaly does not arise, ultimately because the integral kernel of $$\Lambda _{\delta A'}$$ has a milder singularity on the diagonal (Lemma D.7) than $$\Lambda _{\delta g}$$ (see [AS91, PL6]):

$$\begin{aligned} \eta _{\Lambda _{\delta g}}(x,y)\sim _{x\rightarrow y} \frac{1}{8\pi } (\det g)^{\frac{1}{2}} \epsilon _{\mu \nu \rho } \frac{u^\mu }{|| u||^3} du^\nu (g^{-1}\delta g)^\rho _{\tau } u^\tau \delta ^{ab}+\cdots \end{aligned}$$

This difference of singular behavior can be traced to the fact that $\Lambda _{\delta g}$ is a pseudodifferential operator of order -2 while $\Lambda _{\delta A'}$ is a pseudodifferential operator of order -3.

## Footnotes

1 Here $d^*_{A_0}$-exactness is the $A_0$-twisted Lorenz gauge condition and the fact that $\alpha _\textrm{fl}$ is allowed to be a nonhomogeneous differential form is the AKSZ-BV gauge-fixing mechanism. In terms of Faddeev-Popov ghosts $c,{\bar{c}}$, the degree zero component of $\alpha _\textrm{fl}$ is the ghost *c* and the degree two component is $d^*_{A_0}{\bar{c}}$.

2 See, e.g., [Mne19].

3 E.g. in the quantum mechanics path integral for a particle in ${\mathbb {R}}^d$, the “correct” measure on paths is $\prod _t \frac{dp(t)dq(t)}{\sqrt{2\pi \hbar }^d}$, rather than the Lebesgue measure that we are implictly using here.

4 2-framings are trivializations of TM⊕TM, introduced by Atiyah [Ati90]. The canonical 2-framing α is the one for which the Hirzebruch defect $\textrm{sign}(Y) - \frac{1}{6}p_1(2TM,\alpha ) =0$, where *Y* is any 4-manifold with boundary *M* and $p_1(2TM,\alpha )$ is the relative Pontryagin number of the bundle 2*TM* over M×I, trivialized by α over the endpoints of the cylinder.

5 A point $[A_0]\in {\mathcal {M}}$ is “smooth” if the minimal model of the dg Lie algebra $(\Omega ^\bullet (M,{\mathfrak {g}}),d_{A_0},[-,-])$ is the cohomology $H^\bullet _{A_0}$ with *vanishing* $L_\infty$ operations (which implies that ${\mathcal {M}}$ is locally a manifold around $[A_0]$). A flat connection is irreducible if $H^0_{A_0}=0$.

6 For comparisons with nonperturbative answers in Chern–Simons theory one may want to assume that the pair *M*, *G* is such that ${\mathcal {M}}'\subset {\mathcal {M}}$ is an open dense subset. However, results of this paper don’t need this assumption.

7 In fact, $S_{CS}(A_0)$ and $\psi (A_0)$ are locally constant on the entire moduli space ${\mathcal {M}}$, including singular/reducible strata.

8 One has a natural orientation on ${\mathcal {M}}'$, see [JTU20, Theorem 4.5].

9 The extended propagator $\widehat{K}$ and the extended zero-mode inclusion $\widehat{i}({\textsf{a}})$, cf. (154) with $A'=A=A_0$. One also needs to include a special graph consisting of a single edge with the weight $\frac{i}{2\hbar } \langle {\textsf{a}}, \widehat{\Theta }({\textsf{a}})\rangle$, with $\widehat{\Theta }$ as in (154).

10 More precisely: the formal exponential map is the vertical ∞-jet of ϕ. See e.g. [BCM12].

11 This is Proposition 5.8, Corollary 5.9 and Corollary 5.15 put together.

12 To lighten the notations we write $A_0$ instead of $[A_0]$ for a point in ${\mathcal {M}}'$.

13 This is Proposition 4.1, Theorem 4.3, Corollary 4.9, Theorem 4.10, Proposition 4.11 put together.

14 See Theorem 4.17.

15 For a 2-framing α one defines $S_\textrm{grav}({g,\alpha }) =\frac{1}{2} S_{CS}(A_g \oplus A_g)$ by evaluating $\frac{1}{2}$ the Chern–Simons action on the direct sum of the Levi-Civita connection with itself.

16 For $A_0$ an irreducible flat connection, this statement is trivial by Poincaré lemma, cf. Remark 1.5. Otherwise, for $[A_0]\in {\mathcal {M}}\setminus {\mathcal {M}}'$, this statement is not a triviality.

17 This is Theorem 5.17/Proposition 5.19 (266).

18 Connection $\nabla ^{\mathbb {H}}$ arises as the projection—using the desynchronized Hodge decomposition—of the trivial connection in the trivial bundle over $\underline{\mathcal {U}}$ with fiber $\Omega ^\bullet (M,{\mathfrak {g}})$ onto the subbundle of harmonic forms, cf. Remark 2.37.

19 See Theorem 4.23.

20 In [CMW19, CMW20] a similar operator was called “Grothendieck operator”. Here, we use the term Gauss-Manin connection since it is a connection on the (half-density bundle of the) cohomology bundle ${\mathbb {H}}$.

21 We remark that the degree (0, 0, 0) component of the dQME is the ordinary QME $\Delta _{\textsf{a}}Z_{A,A'}({\textsf{a}})=0$ (which is trivial for degree reasons at irreducible connections *A*).

22 Recall that irreducible flat connections satisfy $H^0_{A_0} =0$, in particular in dimension 2 this implies $H^2_{A_0} =0$ and smoothness. For $\gamma \ge 2$, ${\mathcal {M}}' \subset {\mathcal {M}}$ is an open dense subset.

23 For connections of this form we have $S_{CS}(A_0) = \int _\Sigma \langle \phi , F_\alpha \rangle$ which of course vanishes for flat connections α.

24 The Atiyah-Patodi-Singer theorem implies $\frac{\pi i}{4}\psi (A_0,g) = \frac{\pi i}{4}\dim G\, \psi _0(g) - \frac{c_2(G)}{2\pi i}S_{CS}(A_0)$. The second term vanishes by the previous argument, the first term —because the eta invariant of a product manifold satisfies $\psi _0(g_{M\times N}) = \psi _0(g_M)\tau (g_N) + \psi _0(g_N)\tau (g_M)$ where τ denotes the signature, however we have $\psi _0(g_M) = 0$ unless dimM=4k-1 and $\tau (g_N) = 0$ unless dimN=4k.

25 In fact, we only learned about its existence shortly before completion of this paper.

26 Here one should mention the recent paper [CS21] filling a gap in the construction of Bott and Cattaneo.

27 It is known that the Aarhus integral and the LMO invariant are equivalent [BGRT04]. It is conjectured that the KKTL invariant and the LMO invariant are equal, but this is known only up to degree 2 for integral homology spheres (accredited to C. Lescop in private communication of K.W. with G. Massuyeau).

28 “Strong Deformation Retraction data” [GL89], also known in the literature under the names “contraction” [EL53], “homotopy equivalence data” [Cra04], “induction data,” “(*i*, *p*, *K*) triple.”

29 When going to the moduli space, we will only prove it for the subset of smooth irreducible flat connections ${\mathcal {M}}' = \pi (\textrm{FC}')$, i.e. flat connections $A_0$ for which $H^0_{A_0}=0.$

30 This norm is defined by the $L^p$-norms of ω and covariant derivatives up to order *l*, with respect to a given Riemannian metric *g*.

31 The completions in the Sobolev topologies can contain connections with singularities. However, when we say a connection is smooth, we mean that it is a smooth point in $\textrm{FC}$.

32 By “infinitesimal” we everywhere mean “first-order,” as opposed to formal deformations (of infinite order) discussed below.

33 This is because for $k \cdot p \ge n$, one has, for $f,g \in W_{k,p}(M)$ the estimate $||f \cdot g || \le \widetilde{C} ||f||_{k,p} ||g||_{k,p}$ which in turns follows from the Sobolev embedding $W_{k,p}(M) \hookrightarrow C^{0}(M)$, which one has for kp>n on a compact Riemannian manifold. See e.g. [Aub98, Theorem 2.10, Theorem 2.20].

34 It appeared in the context of deformations of complex structures in [Kur65].

35 In the Banach space setting this means smoothness w.r.t. the Fréchet derivative.

36 In Fréchet spaces derivatives are defined using directional (Gateaux) derivatives for which the chain rule holds.

37 For Hodge SDR data, this is the space of harmonic forms in the usual sense of the word.

38 See e.g. [BCM12].

39 Here we are using the graded commutator. Since $d_{A_0}$ and ${\textsf{h}}$ have degree +1 and -1 respectively, this means $[d_{A_0},{\textsf{h}}] = d_{A_0}{\textsf{h}}+ {\textsf{h}}d_{A_0}$.

40 See e.g. [RS71, Section 3], [MW11] for details on Hodge–de Rham decomposition theorem for forms on a Riemannian manifold with coefficients in a flat bundle.

41 Since the inclusion $\Omega ^{\bullet -1}_{k+1,p} \rightarrow \Omega ^{\bullet -1}_{k,p}$ is compact on a compact manifold by the Rellick-Kondranov theorem, we have actually proven that $K_{A_0}$ is compact.

42 The vector bundle with fiber being the space of states of Chern–Simons theory on Σ, a.k.a. the space of WZW conformal blocks on Σ, a.k.a. the Verlinde space. See [APW91].

43 Our assumptions are such that trivializations are guaranteed to exist. See for instance [Fre95].

44 Note that there is no sign ambiguity in the square root line bundle $\textrm{Det}^{\frac{1}{2}}(H^\bullet _{A_0})$, since by Poincaré duality it can be expressed as $\textrm{Det}(H^0)\otimes (\textrm{Det}(H^1))^*$.

45 It is the term $L^{cont}$ in [AS91], formula (PL5). It is the limit $\lim _{y\rightarrow x} (\eta _{A_0}(x,y)-(\cdots ))$ with (⋯) the singular part of η at the diagonal.

46 In principle there could be a multiplicative anomaly when combining the regularized superdeterminants, but here it is absent because the equality is trivially true for β=0 and we are restricting to small β.

47 Here

$$\begin{aligned} \{f,g\} = f\left\langle \frac{\overleftarrow{\partial }}{\partial {\textsf{a}}^{1}}, \frac{\overrightarrow{\partial }}{\partial {\textsf{a}}^{2}}\right\rangle g - f\left\langle \frac{\overleftarrow{\partial }}{\partial {\textsf{a}}^{2}}, \frac{\overrightarrow{\partial }}{\partial {\textsf{a}}^{1}}\right\rangle g \end{aligned}$$

is the BV bracket of *f* and *g*, see e.g. [Mne19, Section 4.4.3].

48 The technology of proving the properties of perturbative partition functions via Stokes’ theorem on the configuration space was developed in particular in [AS94, §6], [Kon94, Kon03].

49 As a distributional form on M×M, the integral kernel of the operator [d,K]=1-P contains the integral kernel of the identity—the delta-distribution on the diagonal in M×M. On the configuration space of pairs of *distinct* points on *M*, ${M\times M\setminus \textrm{Diag}}$, this delta-distribution disappears. However, in the formalism of configuration space integrals, it effectively reappears as the contribution of the principal boundary stratum of the configuration space, see (140), (141) and the discussion below it.

50 For the definition of a superconnection, see e.g. [Igu09, Definition 1.2]. The superconnection (153) can be thought of as a correction of the trivial superconnection $$\delta _{A'}$$ by $$\Delta _{\textsf{a}}$$—a 0-form in $A^{\prime }$.

51 Or: flat (partial) superconnection.

52 Cf. cancellation of principal boundary strata and footnote 49 in the proof of Proposition 4.11.

53 Ray-Singer torsion, as an element of the determinant line $\textrm{Det}\,H_A^\bullet$, is invariant under changes of metric (this result does not require the flat connection to be acyclic).

54 Working with 2-framings has the advantage that 3-manifolds have a canonical 2-framing [Ati90]. Choosing this canonical 2-framing both simplifies the 1-loop part and conjecturally agrees with asymptotics of WRT invariants, see [FG91].

55 By Corollary D.8, the restriction of the propagator to the boundary of configuration space in the desynchronized theory agrees modulo regular terms to the propagator in the case $A=A^{\prime }$, so the analysis of the hidden boundary strata of [AS94] carries over without change.

56 Axelrod and Singer always work under the assumption that $d_A$ is acyclic.

57 This is the construction used by Axelrod and Singer [AS94].

58 Put another way: the parallel transport of the connection $\nabla ^\textrm{Hodge}$ along a path $(A_t,A'_t,g_t)$, t∈[0,1] maps the desynchronized $(A_0,A'_0)$-Hodge decomposition $\Omega =\textrm{Harm}_{A_0,A'_0}\oplus \textrm{im}(d_{A_0})\oplus \textrm{im}(d^*_{A'_0})$ (with metric $g_0$) to the desynchronized $(A_1,A'_1)$-Hodge decomposition $\Omega =\textrm{Harm}_{A_1,A'_1}\oplus \textrm{im}(d_{A_1})\oplus \textrm{im}(d^*_{A'_1})$ (with metric $g_1$) term-to-term.

59 We thank S. Stolz for this remark.

60 These objects are a natural extension of the corresponding objects of Sect. 2.5.4 by allowing variation of metric. By an abuse of notations, we use ${\mathbb {H}}$, $\nabla ^{\mathbb {H}}$ for the extension.

61 This term is completely analogous to the term in the extended action denoted $S_R$ in [BCM12] and is the Hamiltonian (in an appropriate sense) for the Grothendieck connection, hence the superscript *G*.

62 This is a consequence of the following observations: (i) an edge with a KδA leaf plugged in contributes to ${\check{\Phi }}$ as $K\textrm{ad}_{K\delta A} K=K[d^*_{A'},\textrm{ad}^*_{K\delta A}]G= K \textrm{ad}^*_{d_{A'}K\delta A}G = \Lambda _{\widetilde{\delta A'}}$; (ii) a KδA leaf joining an $i({\textsf{a}})$ leaf and continuing with an edge contributes $K\textrm{ad}_{K\delta A}i({\textsf{a}})=G[d^*_{A'},\textrm{ad}^*_{K\delta A}]i({\textsf{a}})=G\textrm{ad}^*_{d_{A'}K\delta A}i({\textsf{a}})={\mathbb {I}}_{\widetilde{\delta A'}}i({\textsf{a}})$. Thus, allowing one KδA leaf in a graph results in graphs of *rZ*. Here Λ and ${\mathbb {I}}$ are as in (129).

63 This discrepancy arises from the fact that the connection $$\nabla ^\textrm{Harm}$$, for an infinitesimal exact shift $$\delta A= d\beta $$ (and $\delta A^{\prime }=0$), shifts a harmonic form χ to $$\chi -K\textrm{ad}_{d\beta }\chi = \chi -\textrm{ad}_\beta \chi +dK\textrm{ad}_\beta \chi + P\textrm{ad}_\beta \chi $$. Here $$\textrm{ad}_\beta \chi $$ corresponds to $$\nabla ^\textrm{gauge}+\nabla _{A'}$$ (Sect. 4.3.1), the term d(⋯) is irrelevant in cohomology and $$P\textrm{ad}_\beta \chi =P\textrm{ad}_{K\delta A}\chi $$ is the discrepancy.

64 Note that in the first order in ϵ it does not matter whether we choose $A^{\prime }=A$ or $$A'=\widetilde{A}$$ in the cohomology comparison map $${\mathfrak {B}}_{\widetilde{A}\leftarrow A;A'}$$.

65 Main statements of this section are a Chern–Simons counterpart of Theorem 6.1 in [BCM12].

66 Note that $\widetilde{Z}={\underline{Z}}+{\underline{r}}\,{\underline{Z}}+\cdots$ with ${\underline{r}}\,{\underline{Z}}$ the generator in the r.h.s. of (229) and ⋯ being a sum of forms of degree ≥2 on ${\mathcal {M}}'$.

67 See [BCM12, Section 2].

68 The sign convention for the Berezinian in the outer integral is: $\mathcal {D}{\textsf{a}}^2\,\mathcal {D}\zeta =\prod _a \mathcal {D}{\textsf{a}}^2_a\,\mathcal {D}\zeta ^a$, for $({\textsf{a}}^2_a,\zeta ^a)$ coordinates on $H^2_A[-1]\oplus H^1_A[1]$ associated to some basis $\chi _a$ in $H^1_A$ and the dual basis $\chi ^a$ in $H^2_A$.
